# Influence of the substitution position on spin communication in photoexcited perylene–nitroxide dyads†

**DOI:** 10.1039/d4sc00328d

**Published:** 2024-03-26

**Authors:** Philipp Thielert, Mélissa El Bitar Nehme, Maximilian Mayländer, Michael Franz, Simon L. Zimmermann, Fabienne Fisch, Peter Gilch, Andreas Vargas Jentzsch, Michel Rickhaus, Sabine Richert

**Affiliations:** a Institute of Physical Chemistry, University of Freiburg Albertstraße 21 79104 Freiburg Germany sabine.richert@physchem.uni-freiburg.de; b Department of Chemistry, University of Zurich Winterthurerstrasse 190 8057 Zurich Switzerland; c Institute of Physical Chemistry, Heinrich Heine University Düsseldorf Universitätsstraße 1 40225 Düsseldorf Germany; d SAMS Research Group, Université de Strasbourg, CNRS, Institut Charles Sadron UPR 22 67000 Strasbourg France; e Department of Organic Chemistry, University of Geneva 30 Quai Ernest-Ansermet 1211 Geneva 4 Switzerland michel.rickhaus@unige.ch

## Abstract

By virtue of the modularity of their structures, their tunable optical and magnetic properties, and versatile applications, photogenerated triplet–radical systems provide an ideal platform for the study of the factors controlling spin communication in molecular frameworks. Typically, these compounds consist of an organic chromophore covalently attached to a stable radical. After formation of the chromophore triplet state by photoexcitation, two spin centres are present in the molecule that will interact. The nature of their interaction is governed by the magnitude of the exchange interaction between them and can be studied by making use of transient electron paramagnetic resonance (EPR) techniques. Here, we investigate three perylene–nitroxide dyads that only differ with respect to the position where the nitroxide radical is attached to the perylene core. The comparison of the results from transient UV-vis and EPR experiments reveals major differences in the excited state properties of the three dyads, notably their triplet state formation yield, excited state deactivation kinetics, and spin coherence times. Spectral simulations and quantum chemical calculations are used to rationalise these findings and demonstrate the importance of considering the structural flexibility and the contribution of rotational conformers for an accurate interpretation of the data.

## Abstract 2

Photoexcited chromophore–radical dyads are suitable for applications in molecular spintronics. We show that the position of attachment of the radical to the chromophore can be used as a means to tune their optical and magnetic properties.

## Graphical abstract

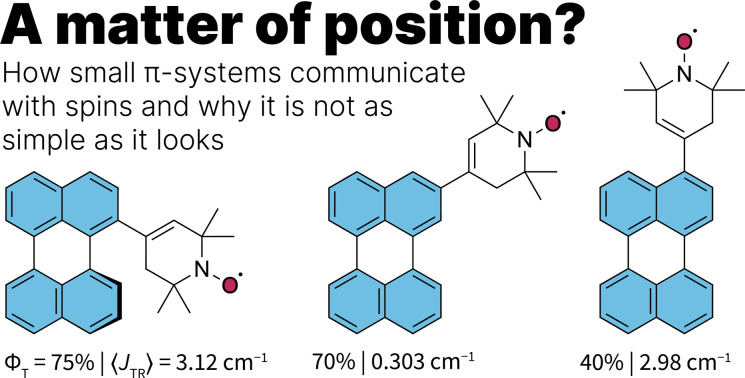

## 1 Introduction

Photogenerated triplet–radical systems have been receiving an increasing amount of attention in recent years due to their great potential for applications in the areas of molecular spintronics and hyperpolarisation.^1–8^ Their structures are highly modular, allowing for the creation of materials with tailored properties and providing an ideal playground to explore the fundamental mechanisms underlying spin communication.

**Fig. 1:**
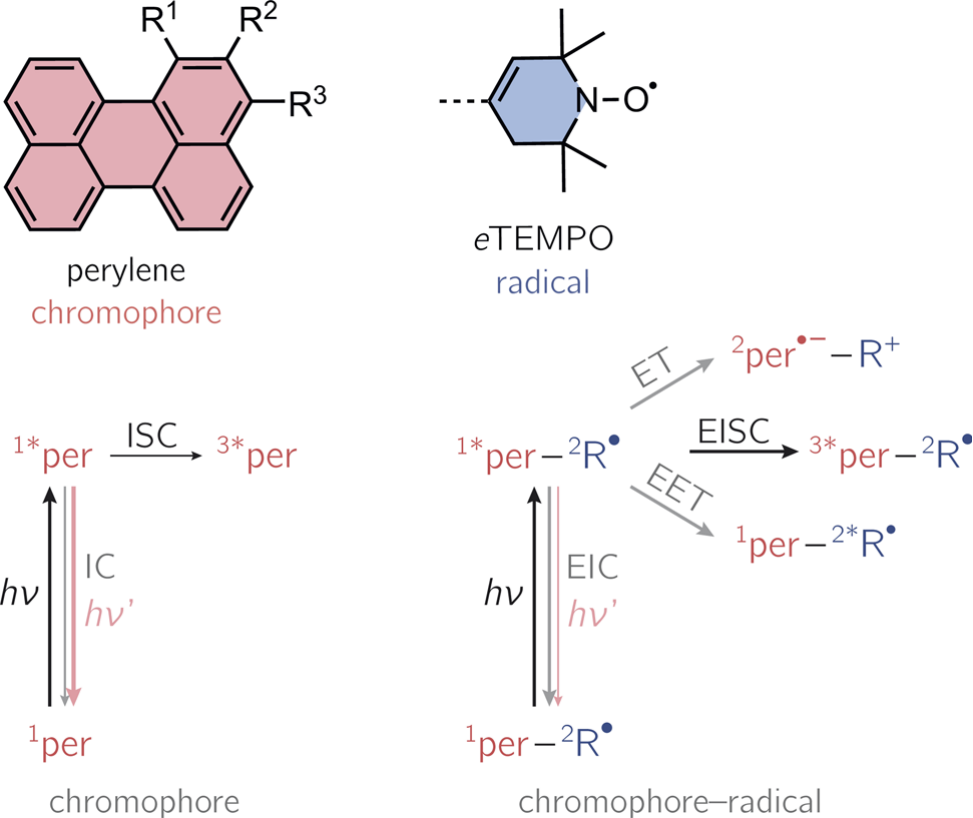
Structural building blocks of the three investigated perylene-based dyads and photoscheme of a chromophore in the absence and presence of an interacting stable radical. Nomenclature: per–1-*e*TEMPO: R^1^ = *e*TEMPO, R^2^ = R^3^ = H; per–2-*e*TEMPO: R^2^ = *e*TEMPO, R^1^ = R^3^ = H; per–3-*e*TEMPO: R^3^ = *e*TEMPO, R^1^ = R^2^ = H. The positions 1, 2, and 3 of the perylene core are referred to as the *bay*, *ortho*, and *peri*-positions, respectively. The numeric superscripts in the photoschemes indicate the spin multiplicity. Abbreviations: R-radical, EISC-enhanced intersystem crossing, EIC-enhanced internal conversion, EET-excitation energy transfer, ET-electron transfer.

Compared to the situation of an isolated chromophore, additional excited singlet state deactivation processes need to be considered in chromophore–radical systems (see Fig. 1). After photoexcitation of the chromophore in proximity of a radical, excitation energy transfer (EET) or electron transfer (ET) may be feasible and compete with chromophore triplet state formation by a process referred to as enhanced intersystem crossing (EISC). While normal, spin–orbit coupling induced, intersystem crossing in these chromophores typically occurs on the nanosecond time scale, EISC can be much faster as the overall spin multiplicity of the coupled chromophore–radical system is conserved.^9–11^

Once the chromophore triplet state is formed, triplet state (T) and radical (R) spin centres will interact, whereby the nature of their interaction is mainly determined by the magnitude of the exchange interaction, *J*_TR_, between them. If *J*_TR_ is larger than any other magnetic interactions in the system, quartet states may be formed by spin mixing, which have been shown to exhibit favourable properties for applications in the field of quantum sensing.^3,7,8^ The spin-mixing process underlying quartet state formation may either be mediated by dipolar interactions or spin–orbit coupling,^2,12–16^ but mechanistic details still remain to be disclosed.

Systematic studies of the properties of chromophore–radical dyads, carried out in recent years, are starting to reveal important design guidelines and provide further mechanistic insight that will prove to be crucial for the applicability of these systems as building blocks of practical spintronic devices. For instance, it was shown that the coherence properties of the radical have a direct influence on the spin coherence times of the quartet states formed by spin mixing in strongly-coupled triplet–radical systems.^7,17^ The use of trityl radicals instead of nitroxides was found to lead to much improved quartet state coherence times, but typically entails a poor EISC yield due to EET taking place (loss channel).^17,18^

On the other hand, the feasibility of ET does not necessarily impede triplet state formation, as it has been shown, on the example of a PDI–TEMPO system, that ET followed by fast charge recombination to the chromophore triplet state (inverted kinetics) can lead to a sizeable triplet state formation yield of more than 50% for chromophores with low-lying triplet states.^19^ In the absence of processes like ET and EET, competing with chromophore triplet state formation, it was suggested that the yield of EISC correlates with the dipolar and exchange interactions between the chromophore and radical electrons (and therefore depends on the chromophore–radical distance).^20^

All of these observations are highly valuable. Yet, the amount and variety of structures investigated to date is still too small in order to draw truly general and irrefutable conclusions, emphasising the need for further systematic studies. To date, several different chromophore, radical, and linker combinations have been explored, but a pertinent question, which has not been addressed so far is the dependence of the optical and magnetic properties of the system on the substitution position at which the radical is attached to the chromophore.

Here, we present the synthesis and transient spectroscopic characterisation of three perylene–nitroxide dyads and their respective bromoperylene counterparts. Since previous studies suggested that the yield of EISC depends on the chromophore–radical distance,^20^ the nitroxide radical is linked directly to the core of the perylene chromophore with the aim to maximise triplet state formation. To achieve a direct linkage, we use a derivative of the well-known 2,2,6,6-tetramethyl-piperidin-1-oxyl (TEMPO) radical with a double bond in the piperidine ring (see Fig. 1).^20–22^ This radical, referred to as *e*TEMPO in the following, can be tethered to the perylene core by well-established Suzuki–Miyaura coupling. The three investigated perylene–*e*TEMPO dyads are shown in Fig. 1. They are structural isomers of each other, only differing with respect to the core position chosen for the attachment of the radical substituent. In the following, these structures will be referred to as per–1-*e*TEMPO, per–2-*e*TEMPO, and per–3-*e*TEMPO, while the abbreviations 1-Br, 2-Br, and 3-Br will be used for the respective triplet state precursors 1-bromoperylene, 2-bromoperylene, and 3-bromoperylene.

A detailed spectroscopic study of the perylene–*e*TEMPO dyads using transient optical and electron paramagnetic resonance (EPR) techniques reveals major differences in their excited state properties. Between the three structural isomers, the excited singlet state lifetime of the perylene chromophore varies by roughly an order of magnitude. In addition, substantial variations in the triplet state formation yield from ∼40% to 75% are observed. Transient EPR experiments demonstrate quartet state formation for all three compounds, although differences in the excited state exchange coupling interactions (*J*_TR_) up to an order of magnitude are suggested by quantum chemical calculations. Since variable-temperature NMR experiments revealed the contribution of several rotational conformers, the possible influence of the latter on data interpretation was characterised using quantum chemical calculations. We find that the magnitude of the exchange interaction can vary substantially between conformers, implying (i) the need to account for geometric flexibility and rotational conformers in the analysis of spectroscopic data and (ii) a temperature-dependence of *J*_TR_. Given the strong influence of the substitution position on the optical and magnetic properties, our results demonstrate that the position of attachment of the radical to the chromophore can be used as a means to tune the photophysical properties of the system.

## 2 Results and discussion

### Synthesis

The first step in preparing per–3-*e*TEMPO involved the bromination of perylene as previously described in the literature,^23^ yielding 3-Br with small amounts of unreacted perylene. An analytically pure sample of 3-Br could be obtained by recrystallisation and was used for the photophysical characterisation of 3-Br. Since the presence of perylene does not adversely affect the Suzuki–Miyaura coupling with Bpin–*e*TEMPO, we were able to prepare per–3-*e*TEMPO in 39% yield using the crude 3-Br. As an alternative procedure, per–3-*e*TEMPO was also prepared using a minor side product of the iridium-catalysed borylation of perylene:^24^ 3-Bpin (0.4%). The Suzuki–Miyaura coupling of 3-Bpin with I–*e*TEMPO resulted in the formation of per–3-*e*TEMPO with an improved yield of 47%. For the preparation of per–2-*e*TEMPO, the mentioned iridium-catalysed borylation of perylene^24^ gave 2-Bpin–perylene in 20% yield. The subsequent coupling between 2-Bpin and I–*e*TEMPO resulted in the formation of per–2-*e*TEMPO with a yield of 67%. 2-Br was obtained starting from 2-Bpin by halodeboronation using CuBr_2_.^24^

In comparison to its isomers, the synthesis of per–1-*e*TEMPO was more challenging. To orient the selectivity towards the *bay* position, we carried out the nitration of perylene^25^ which gave a mixture of the *bay* and *peri* isomers (1-NO_2_ and 3-NO_2_). The latter were readily separated by column chromatography. The reduction of the nitro group was successfully achieved using standard Pd/C conditions,^25^ resulting in the target compound 1-NH_2_ with an 83% yield. The latter was used in the next step without additional purification. While the classical Sandmeyer reaction failed in our hands, 1-Br was obtained in 29% yield following a similar procedure for the conversion of anilines to aryl bromides reported by Stack and co-workers.^26^ The reported procedure represents a convenient alternative due to the short reaction time, insensitivity to moisture and air, and the fact that no prior isolation of the diazonium salts is necessary. The per–1-*e*TEMPO compound was synthesised through Suzuki–Miyaura coupling of 1-Br with Bpin–*e*TEMPO, resulting in the formation of the targeted product with a 54% yield. However, in this case, purification by preparative thin-layer chromatography (prepTLC) was necessary since degradation was observed during column chromatography. Additional details on the synthesis and characterisation, by NMR spectroscopy and mass spectrometry, are presented in the ESI.†

To further confirm the structure and purity of the prepared compounds, we performed the *in situ* reduction of both per–1-*e*TEMPO and per–2-*e*TEMPO following a procedure reported by Tidwell (see the ESI†).^27^ Both per–1-*e*TEMPOH and per–2-*e*TEMPOH were extensively characterised by NMR spectroscopy and their structures could be confirmed (see the ESI, Section 1.3†).

### UV-vis spectroscopy

**Fig. 2:**
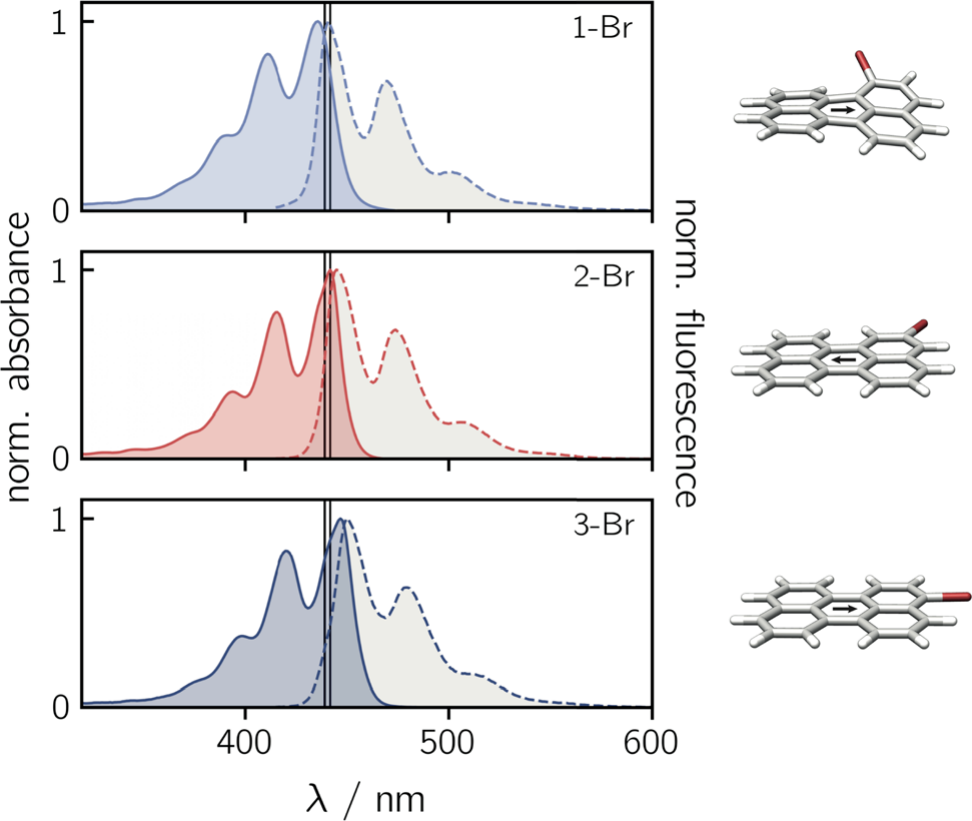
Normalised UV-vis absorption (solid lines) and fluorescence (dashed lines) spectra of the three bromoperylene precursors in toluene. The black vertical lines indicate the positions of the absorption and fluorescence intensity maxima of perylene. To the right, the optimised molecular structures are shown. The black arrow indicates the orientation of the transition dipole moment calculated at the CAM-B3LYP/def2-TZVP level of theory.

Before focusing on the perylene–nitroxide dyads, the bromoperylene precursor molecules were studied as model systems for the investigation of substitutional effects on the photophysical properties of the perylene chromophore. Fig. 2 shows the UV-vis absorption and fluorescence spectra of the three bromoperylenes. It can be seen that, relative to bare perylene, the absorption maximum of 1-Br is slightly blue-shifted, while the absorption maxima of 2-Br and 3-Br are both red-shifted. For 1-Br, we also observe an increase in the Stokes shift by about 100 cm^−1^, relative to the other isomers and perylene which show similar Stokes shifts. An overview of the photophysical properties is provided in Table 1.

**Table 1:** Overview of the photophysical properties of the bromoperylene precursors in toluene at 295 K. *λ*_abs_: absorption maximum; *λ*_flu_: fluorescence maximum; *S*_0,0_: excited singlet state energy; *Φ*_F_: fluorescence quantum yield; *τ*_F_: fluorescence lifetime

Compound	*λ*_abs_/nm	*λ*_flu_/nm	Stokes shift/cm^−1^	*S*_0,0_/eV	*Φ*_F_	*τ*_F_/ns
Perylene	439	442	140	2.82	0.87	3.66
1-Bromoperylene	436	441	260	2.82	0.064	(<0.27)^a^
2-Bromoperylene	442	445.5	180	2.80	0.91	3.85
3-Bromoperylene	447	450.5	170	2.76	0.81	3.43

a Estimated value.

Geometry optimisations of the bromoperylene structures at the B3LYP/def2-TZVP level of theory, as visualised in Fig. 2 and described in the ESI (Section 4.1†), reveal a significant twist of the perylene core for 1-Br due to steric constraints (dihedral angle of ∼24° between the two naphthalene units). As suggested also in previous studies,^28^ we observe that substitution in this sterically demanding position 1 causes a reduction in the compound's stability and its fluorescence quantum yield. While the fluorescence quantum yield of perylene, 2-Br, and 3-Br is well above 80% in toluene at room temperature, that of 1-Br was measured to amount to only ∼6% (see Table 1). A similar trend is also reflected in the fluorescence lifetimes. For perylene, 2-Br, and 3-Br, lifetimes between 3.4 ns and 3.9 ns were measured, whereas 1-Br is characterised by a sub-nanosecond lifetime which is too short to be quantified accurately by single photon timing. Further details on the measurements and data analysis are given in the ESI (see Section 2.1, Fig. S20†).

Contrary to what might be expected, the triplet yields of all bromoperylenes are only of the order of 5%.^28^ It has been discussed that the lack of a pronounced internal heavy atom effect in bromoperylenes is due to the absence of a triplet state that is energetically close to the fluorescing state.^29^ It was argued that, for an internal heavy atom effect to occur, the energy difference between the chromophore S_1_ state and the accepting triplet state has to be small enough for intersystem crossing to compete effectively with fluorescence.^29^

The low triplet yield implies that internal conversion is remarkably and unusually efficient in 1-Br with *Φ*_IC_ ∼ 0.88. It has been suggested that this might be due to additional promoting vibrational modes arising from the non-planarity of the structure.^28^

Having analysed the pertinent effect of core substitution on the photophysical properties of the perylene chromophore, we now turn to the photophysical properties of the perylene–*e*TEMPO dyads. The UV-vis absorption and fluorescence spectra of the dyads are shown in the ESI (Fig. S18†). Relative to perylene (see Fig. S17†), we observe a slight red-shift of the UV-vis absorption and fluorescence maxima for all three compounds, with the largest shifts measured for per–3-*e*TEMPO. The Stokes shift increases in the series per–1-*e*TEMPO, per–2-*e*TEMPO, per–3-*e*TEMPO, from about 100 cm^−1^ to ∼540 cm^−1^, while the energy of the chromophore S_1_ state is only slightly affected by the *e*TEMPO substituent (see Table S3†). The latter decreases within the series from 2.80 eV for per–1-*e*TEMPO to 2.74 eV for per–3-*e*TEMPO.

**Fig. 3:**
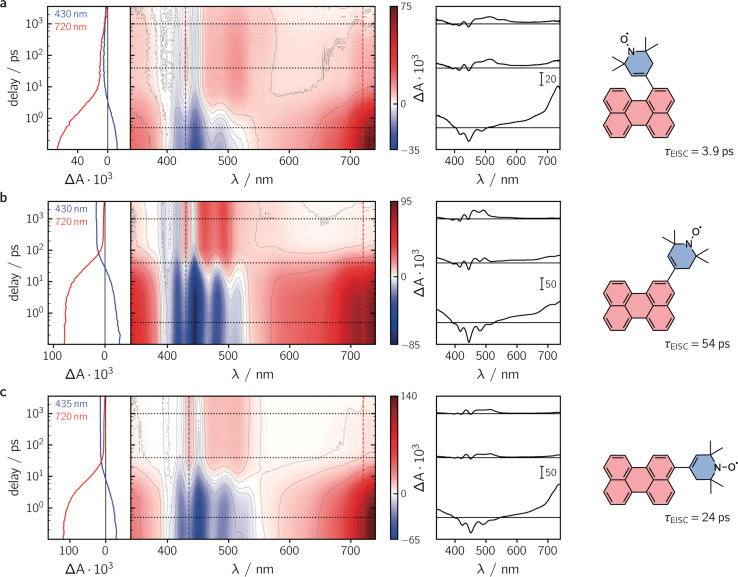
Contour plots of the fsTA data recorded for the three perylene–*e*TEMPO dyads dissolved in toluene solution at room temperature after photoexcitation at 400 nm. (a) per–1-*e*TEMPO, (b) per–2-*e*TEMPO, (c) per–3-*e*TEMPO. The red and blue colour coding in the contour plot represents positive and negative signals, respectively. The vertical coloured lines indicate the positions corresponding to the kinetic traces shown in the left panel, while the dotted horizontal lines indicate the time delays associated with the spectra shown on the right.

To characterise the kinetic processes occurring after photoexcitation of the dyads, femtosecond transient UV-vis absorption (fsTA) measurements were carried out in toluene solution at room temperature. Contour plots of the data obtained for the three compounds as well as exemplary kinetics are shown in Fig. 3. For reference, the data measured for perylene, as well as a description of the experimental setup and parameters, can be found in the ESI (see Sections 2.4 and 2.6, Fig. S22†).

Immediately after photoexcitation, we observe the typical excited state absorption (ESA) signals of the excited singlet state of the perylene chromophore in all three cases, dominating almost the entire visible range of the spectrum. Superimposed on these positive Δ*A* signals are the negative contributions from the ground state bleach in the range ∼350–460 nm and stimulated emission from about 430 to 550 nm. These initial signals are then found to decay with different time constants, depending on the substitution position. The signal decay is accompanied by a simultaneous rise of new ESA signals that can be attributed to the excited triplet state of perylene.^30^

Although the qualitative behaviour is the same for all three dyads, the time constants *τ*_S1_ of the chromophore excited singlet state deactivation are found to depend strongly on the substitution position. A global kinetic analysis of the fsTA data, as shown and described in detail in the ESI (see Section 2.6, Fig. S23–S26†), reveals time constants *τ*_EISC_ of 3.9 ps, 54 ps, and 24 ps, for per–1-*e*TEMPO, per–2-*e*TEMPO, and per–3-*e*TEMPO, respectively. Triplet state formation is thus found to be faster in per–1-*e*TEMPO by a factor of about six compared to per–3-*e*TEMPO, and about an order of magnitude with respect to per–2-*e*TEMPO.

By careful analysis of the time behaviour of the ground state bleach, approximate triplet state formation yields can be calculated from the fsTA data as explained previously^19^ and summarised in the ESI (Section 2.5†). Surprisingly, no correlation between the time constant of triplet state formation and the triplet yield was observed. The relatively fast excited state deactivation observed for per–3-*e*TEMPO results in the lowest triplet state formation yield of about 40%, while a very high value of about 70% was obtained for per–2-*e*TEMPO, which showed the slowest excited state deactivation. For per–1-*e*TEMPO, a triplet yield of about 75% could be determined although the uncertainty on this value might be as high as ± 5% due to photodegradation taking place during the fsTA measurements.

Given the relatively high triplet state formation yields for all three dyads and that no perylene ion signatures are observed in the fsTA data, it can be assumed that other chromophore excited singlet state deactivation pathways, such as ET and EET, competing with EISC, can be excluded for these dyads. This statement is also supported by calculations of the Förster-type excitation energy transfer rate constants as shown in the ESI (Section 2.3†). Although there is an almost perfect overlap between the emission spectrum of perylene and the absorption spectrum of *e*TEMPO, the excitation energy transfer rate constants are found to be non-competitive due to the small molar absorption coefficient of *e*TEMPO and an unfavourable relative orientation of the transition dipole moments of chromophore and radical (*i.e.* small *κ*^2^ values).

The fact that the triplet yields of all dyads are significantly less than one, despite a fast (picosecond) deactivation of the chromophore excited singlet state, suggests a non-negligible contribution of enhanced internal conversion.

### EPR characterisation of the dyads

To characterise the EPR properties of the three dyads in the absence of photoexcitation, continuous wave (cw) EPR measurements were performed in toluene solution at the X-band (9.75 GHz) at room temperature. The data are shown in Fig. S27 in the ESI.† All three spectra exhibit the characteristics of a typical nitroxide spectrum, showing three lines with a peak separation of ∼1.5 mT that result from the coupling of the unpaired electron spin to the ^14^N nucleus (*I* = 1) of the nitroxide group. Interestingly, we observe small differences in the isotropic *g* value, ranging from 2.0051 for per–1-*e*TEMPO to 2.0057 for per–3-*e*TEMPO. For per–2-*e*TEMPO, an intermediate value of 2.0055 is obtained.

**Fig. 4:**
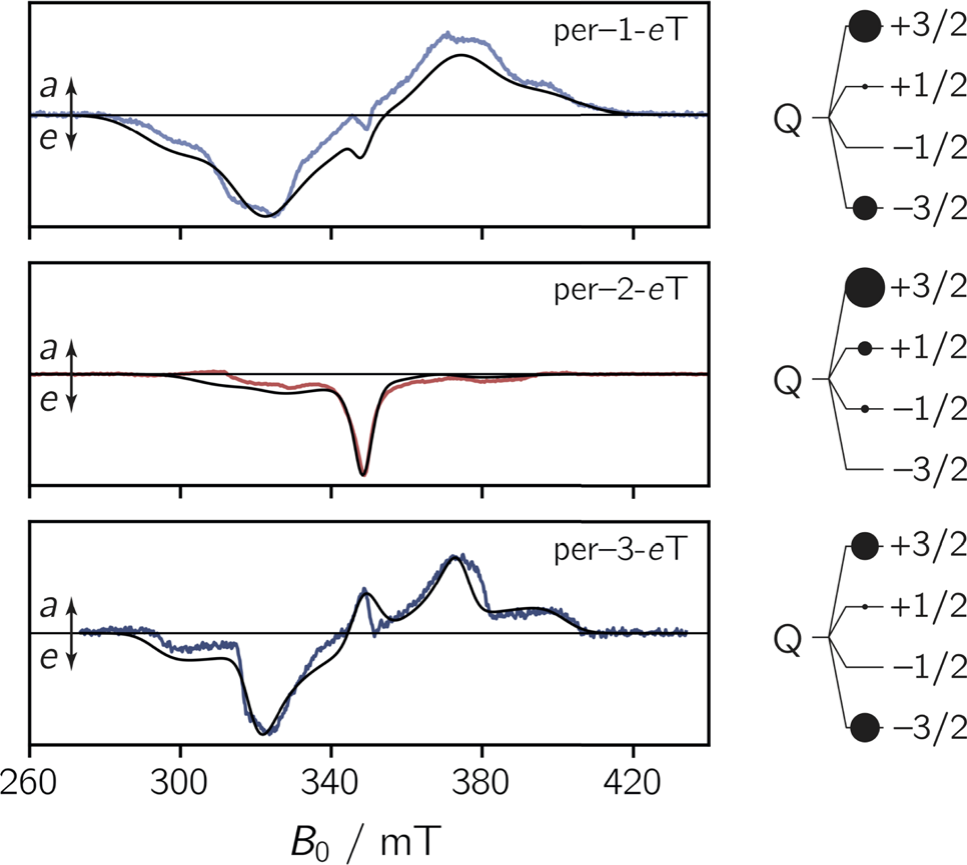
Transient continuous wave EPR (trEPR) spectra of per–1-*e*TEMPO, per–2-*e*TEMPO, and per–3-*e*TEMPO at 0.8 μs after laser excitation recorded at the X-band in frozen toluene solution at 80 K together with quartet state simulations using a spin Hamiltonian approach. The simulation is shown as a black solid line, the relative quartet state populations are shown next to the data and the complete set of simulation parameters can be found in the ESI.†

To explore the magnetic properties of the species generated after photoexcitation of the perylene chromophore, transient continuous wave EPR (trEPR) measurements were performed at the X-band in isotropic frozen toluene solution at 80 K. The spectra obtained at 0.8 μs after laser excitation of the three dyads are shown in Fig. 4.

The shape and width of the trEPR spectra of per–1-*e*TEMPO and per–3-*e*TEMPO are similar to that of the respective bromoperylene precursors, as shown in the ESI (see Fig. S29†). The spectra are characterised by an *eeeaaa* multiplet polarisation (*a*: enhanced microwave absorption, *e*: microwave emission). In addition, an *a*/*e* polarisation pattern is observed in the centre of the spectra. In the case of per–2-*e*TEMPO, this additional central feature (net polarisation) is entirely in emission and dominates the spectral shape. The multiplet polarisation is comparatively low in intensity and also entirely in emission.

The observation of net polarisation in the centre of the spectrum is typical for a quartet state. To further confirm the quartet nature of the signal, numerical simulations of the data were carried out using the EasySpin software package.^31,32^

For the simulation of the quartet state trEPR spectra of the dyads, the characteristic magnetic parameters of their triplet state and radical precursors (*D*_T_, *E*_T_, *g*_T_, **g**_R_) were kept fixed, so that only the coupling parameters (*D*_TR_, *J*_TR_), the relative quartet and doublet state populations, and linewidths were left to vary. The **g**-tensor of the radical was obtained from a simultaneous fit of a room temperature cw EPR spectrum and a pulse Q-band EPR spectrum recorded at 80 K (see Fig. S28†), while the triplet state parameters were obtained from a fit of the trEPR spectra of the corresponding bromoperylenes recorded in frozen toluene solution at 80 K (see the ESI, Section 3.3,† for details and parameters).

The simulations of the experimental quartet state trEPR spectra are shown in Fig. 4 and a complete list of all simulation parameters is given in the ESI (Table S7†). Considering the asymmetric nature of the radical (with respect to the chromophore–radical bonding axis), the slight mismatch between experiment and simulation could be explained by the contribution of several rotational conformers with different magnetic parameters.

To verify this anticipated presence of rotational conformers, variable-temperature NMR measurements were carried out on the reduced forms of per–1-*e*TEMPO and per–2-*e*TEMPO, namely per–1-*e*TEMPOH and per–2-*e*TEMPOH (see the ESI Section 1.3†). The measurements clearly showed the contribution of several species, demonstrating the existence of conformers with approximately equal energies. In the case of per–1-*e*TEMPOH, it was further possible to determine the rotational barrier height experimentally. Finally, DFT calculations confirmed the assignment of the experimentally determined barrier to the rotation around the C–C single bond connecting chromophore and radical as the measured (∼63 kJ mol^−1^) and calculated (*ω*B97XD/6-311G(d,p), 70 kJ mol^−1^) values are in excellent agreement. Details on the DFT calculations are given in the ESI (Section 4.3†).

### Coherence properties

**Fig. 5:**
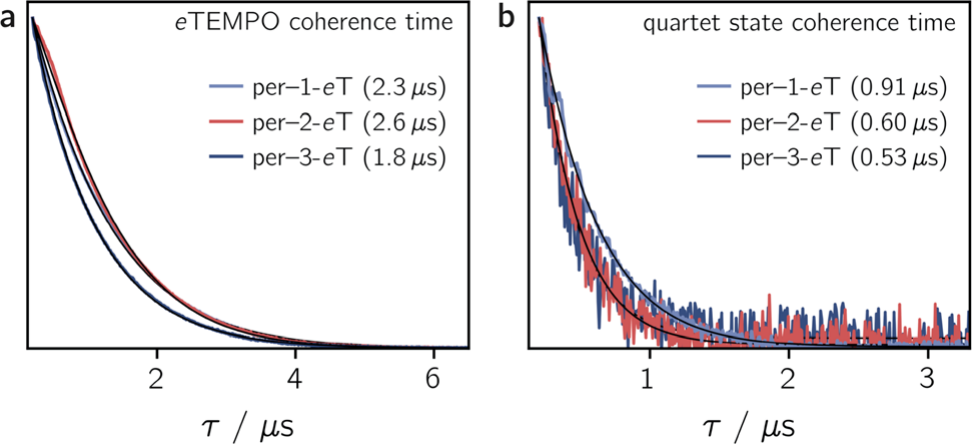
Spin echo decay for per–1-*e*TEMPO, per–2-*e*TEMPO and per–3-*e*TEMPO measured at the Q-band in frozen toluene solution at 80 K in the dark (a) and after photoexcitation at 435 nm (b). The spin coherence times (*T*_m_), obtained from a stretched exponential fit, are indicated. Any residual dark state signal was suppressed during the quartet state *T*_m_ measurements by application of a pre-saturation pulse.

To determine the influence of the substitution position on the spin relaxation times of both the nitroxide radical and the photogenerated quartet state, pulse EPR measurements were carried out in frozen toluene solution at the Q-band at 80 K. The spin echo decay of the radical, measured for the three dyads in the dark, is shown in Fig. 5a. The data were recorded at a field position corresponding to the intensity maximum of the field-swept echo-detected EPR spectrum (see Fig. S30†). The fit of a stretched exponential function of the form *V*_SE_ = exp(−(2*τ*/*T*_m_)^*β*^) to the data yielded the spin coherence times, *T*_m_, indicated in Fig. 5. The complete set of fitting parameters is listed in Table S8 in the ESI.†

It is surprising to see that even the dark state coherence times of the *e*TEMPO substituent are affected markedly depending on the position of attachment to the perylene core. This might be due to different steric strains compromising the mobility of the radical spin centre. The longest coherence time of 2.6 μs is observed for per–2-*e*TEMPO, while per–1-*e*TEMPO and per–3-*e*TEMPO are characterised by dark state *T*_m_ values of 2.3 μs and 1.8 μs, respectively (with *β* values close to one in all cases, see Table S8†). A similar trend is also observed for the longitudinal relaxation time *T*_1_ of the radical, which amounts to 0.32 ms, 0.46 ms and 0.28 ms, for per–1-*e*TEMPO, per–2-*e*TEMPO, and per–3-*e*TEMPO, respectively, in frozen toluene at 80 K. The data and fits are shown in the ESI (see Fig. S32–S34†).

In contrast to expectations based on the high triplet state formation yields, the light-induced pulse EPR signals of all three perylene–*e*TEMPO dyads are found to be very weak (see Fig. S30 in the ESI†). Nevertheless, the quartet nature of these light-induced signals could be unambiguously confirmed by making use of transient nutation measurements as shown and described in the ESI (see Fig. S31†). Interestingly, only the net polarisation of the quartet state, associated with the quartet |*m*_S_ = +1/2〉 ↔ |*m*_S_ = −1/2〉 transition, could be detected by pulse EPR at 80 K. The absence of a quartet multiplet polarisation was also observed previously for similar systems^8^ and might be due to the fact that spin relaxation is typically found to be significantly faster for any signals corresponding to the quartet |*m*_S_ = ±1/2〉 ↔ |*m*_S_ = ±3/2〉 transitions.^3^

Fig. 5b shows the spin echo decay measured at the maximum of the light-induced pulse EPR signal corresponding to the quartet |*m*_S_ = +1/2〉 ↔ |*m*_S_ = −1/2〉 transition. The respective magnetic field positions are marked in Fig. S30 in the ESI.† The quartet state coherence times *T*_m_, as indicated in Fig. 5, were again obtained by fitting a stretched exponential function to the data. The longest *T*_m_ of 0.91 μs is obtained for per–1-*e*TEMPO, while the spin coherence times of the quartet states of per–2-*e*TEMPO and per–3-*e*TEMPO are similar and markedly shorter (0.60 and 0.53 μs, respectively).

These results show that a high EISC yield does not necessarily imply an intense light-induced pulse EPR signal, as might be assumed. A likely reason for this is that the low pulse EPR signal is essentially due to fast spin relaxation in combination with the rather poor (*i.e.* hundreds of nanoseconds) time resolution of the pulse EPR technique.

The fact that, compared to previously studied systems,^3,7,8,17^ we observe relatively short coherence times for both the radical and the quartet state of the perylene–*e*TEMPO systems studied here, suggests that fast spin relaxation might indeed be the main reason for the weak pulse EPR signal. This is also in line with the observation that the light-induced pulse EPR signal of per–1-*e*TEMPO, which shows the longest quartet state coherence time, is most intense. Since previous studies demonstrated that the spin coherence time of the quartet state is correlated with that of the radical, we anticipate that both the spin relaxation times and the quartet state pulse EPR signal intensity could be improved considerably by the choice of a different radical.^17^

Regarding the influence of the substitution position on the spin coherence time of the quartet state, we note that per–1-*e*TEMPO shows the slowest spin coherence decay among the three dyads. Substitution in position 1 is synthetically most challenging and leads to the structure with the largest steric strain. The resulting lower structural flexibility is likely to be the cause for the slower spin relaxation observed here.

### Quantum chemical calculations of rotational barriers and excited state exchange couplings

Since variable temperature NMR measurements of per–1-*e*TEMPOH revealed the contribution of several conformers to the experimental spectra of the dyads, their structures and properties were explored with the help of quantum chemical calculations.

To locate local minima (*i.e.* conformers) on the potential energy surface of the electronic ground state, a relaxed surface scan was carried out using the ORCA program package.^33^ A full rotation around the C–C single bond connecting the perylene chromophore and the *e*TEMPO radical was performed in steps of 5°. At every step, the structure was optimised using DFT at the BP86/def2-TZVP level of theory while keeping the desired dihedral angle fixed.

**Fig. 6:**
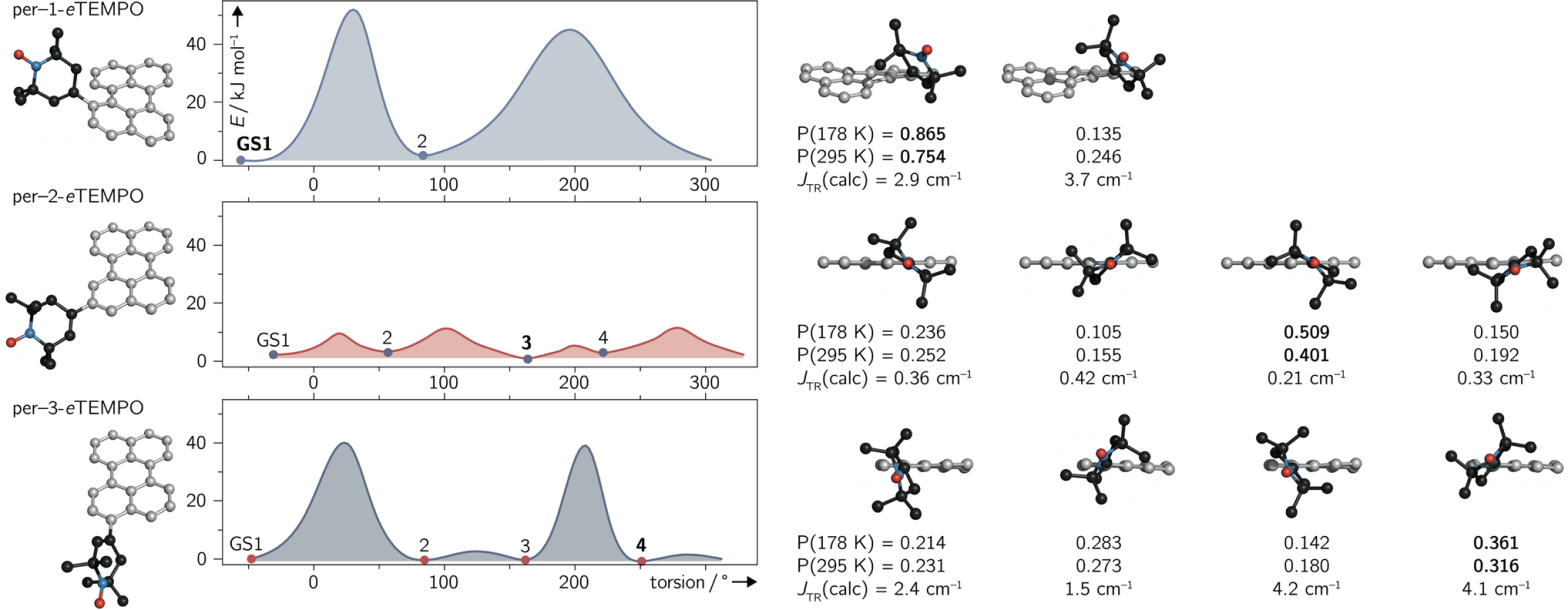
Ground state conformations and rotational barriers calculated for per–1-*e*TEMPO, per–2-*e*TEMPO, and per–3-*e*TEMPO, employing a relaxed surface scan (BP86/def2-TZVP). The calculated excited state exchange interactions *J*_TR_ and relative occupation probabilities P (at two different temperatures) are indicated underneath the respective ground state structures.

The structures corresponding to the located minima were then reoptimised without any constraints to obtain accurate ground state energies. The results of the surface scans and relative energies of the local ground states are visualised for all three dyads in Fig. 6.

For per–1-*e*TEMPO, two local minima were located, while four ground state conformations were found for both per–2-*e*TEMPO and per–3-*e*TEMPO. For each of these conformers, the excited state exchange interaction *J*_TR_ was then calculated as described in detail previously^34^ and outlined in the ESI (see Section 4.4†). The uncontracted version of the difference-dedicated configuration interaction method with two degrees of freedom (DDCI2) was applied on a state-averaged CASSCF(3,3) wavefunction (one quartet state and eight doublet states – all equally weighted) using the def2-SVP basis set. The results are shown underneath the respective structures in Fig. 6, where a positive value of *J*_TR_ indicates ferromagnetic coupling.

While all conformers are ferromagnetically coupled, the results demonstrate that the magnitude of the exchange interaction can vary substantially, depending on the relative orientation of chromophore and radical. This observation is crucial, since it underlines the need to consider and study the contribution of different molecular conformations carefully for a correct interpretation of spectroscopic data of any exchange-coupled systems. In the case of chromophore–radical systems, this may become particularly important when investigating systems that are not symmetric with respect to the chromophore–radical bonding axis (typically involving asymmetric radicals).

Tables S9–S11 in the ESI† summarise the relative energies of all conformers as well as their calculated *J*_TR_ values. Considering the occupation probability P of the different ground states at a specific temperature, a Boltzmann-weighted average value for *J*_TR_ can be calculated. At room temperature we obtain 〈*J*_TR_〉 = 3.12 cm^−1^ for per–1-*e*TEMPO, 〈*J*_TR_〉 = 0.303 cm^−1^ for per–2-*e*TEMPO, and 〈*J*_TR_〉 = 2.98 cm^−1^ for per–3-*e*TEMPO, while the corresponding values at the freezing point of toluene (178 K) are 3.04 cm^−1^, 0.285 cm^−1^, and 2.98 cm^−1^, respectively. This demonstrates that the presence of several conformers also implies that the average exchange interaction will vary as a function of temperature. The variation can be quite small, as observed here, but may be much larger for different structures. For instance, the use of a linker between chromophore and radical, such as a phenyl bridge, is expected to lead to much larger variations of *J*_TR_ between conformers and therefore also to larger variations of 〈*J*_TR_〉 as a function of temperature. Consequently, special attention needs to be paid to the influence of 〈*J*_TR_〉 when comparing spectroscopic data recorded at different temperatures.

## 3 Conclusions

In the present study, we investigated the influence of substitutional effects on the excited state properties of three perylene–nitroxide dyads using a combination of complementary techniques including fsTA and transient EPR spectroscopies. The synthesis of per–1-*e*TEMPO proved to be particularly challenging due to steric constraints. The latter cause a twist of the perylene core, entailing a high reactivity, reduced photostability, and substantially altered photophysical properties compared to the other two dyads.

The time constant of triplet state formation, *τ*_EISC_, was shown to depend strongly on the substitution position. Compared to per–1-*e*TEMPO, *τ*_EISC_ of per–2-*e*TEMPO is larger by about an order of magnitude, while triplet state formation is only slowed down by a factor of about six in per–3-*e*TEMPO.

Although distinct differences were observed, the EISC yield is relatively high for all three compounds, confirming the hypothesis that the yield of triplet state formation in photoexcited chromophore–radical systems is correlated with the chromophore–radical distance (in systems where ET as well as EET can be neglected).^20^ However, since the substitution position clearly has a major influence, as shown in this study, a direct comparison can only be valid when considering the same substitution pattern.

Variable-temperature NMR measurements revealed the contribution of several rotational conformers to the dyad spectra. Quantum chemical calculations were employed to rationalise these findings and suggest that there is a stringent need to consider the influence of such conformers for a correct interpretation of the spectroscopic data of any exchange-coupled system, as the magnitude of the exchange interactions can vary markedly between conformers. In addition, a temperature dependence of *J*_TR_ can be expected in these cases. While a different connection of the radical to the linker (*meta vs. para*) typically alters the sign of *J*_TR_, the present study shows that the position of attachment of the radical to the chromophore does only affect the magnitude but not the sign of *J*_TR_.

The pertinent influence of the substitution position is also evident when comparing the coherence properties of the quartet states formed by spin mixing. Compared to both per–3-*e*TEMPO and per–2-*e*TEMPO, the quartet state coherence time in per–1-*e*TEMPO is increased by almost a factor of two. Despite the relatively high EISC yields measured for all three perylene–nitroxide dyads, their pulse EPR signals at 80 K are surprisingly weak and comparatively short spin coherence times were obtained. We suggest that the weak pulse EPR signals are mainly caused by fast spin relaxation and anticipate that the pulse EPR signal intensities and spin coherence times may be increased significantly by the choice of a different radical. Coherence times of a few microseconds should be within reach, making such perylene-based systems suitable as building blocks of molecular spintronic devices.

## Data availability

The data supporting the findings of this study are available within the article and in the ESI.†

## Author contributions

Spectroscopic characterisation of the compounds, EPR data acquisition and analysis, fsTA data analysis, draft editing and data visualisation P. T.; synthesis and characterisation of the compounds, draft editing M. E. B. N.; spectroscopic characterisation of the compounds, EPR data acquisition, draft editing M. M.; quantum chemical calculations and analysis of excited state exchange couplings, draft editing M. F.; fsTA data acquisition, draft editing S. L. Z.; synthesis and compound characterisation F. F.; supervision of the fsTA experiments, funding acquisition, draft editing P. G.; supervision of the synthesis, DFT calculations of conformer geometries and rotational barriers, draft editing A. V. J.; supervision of the synthesis, funding acquisition, draft editing and data visualisation M. R.; conceptualisation, project administration, funding acquisition, supervision of the optical and EPR experiments, original draft writing, data visualisation S. R.

## Conflicts of interest

There are no conflicts to declare.

## Supplementary Material

SC-015-D4SC00328D-s001
