# Supplementary material for: Influence of the substitution position on spin communication in photoexcited perylene–nitroxide dyads
Source: Chem Sci. 2024 Mar 26;15(20):7515–23. doi: 10.1039/d4sc00328d (PMC11110163; doi:10.1039/d4sc00328d)
Supplement: SC-015-D4SC00328D-s001 [file SC-015-D4SC00328D-s001.pdf]

Electronic Supplementary Material (ESI) for Chemical Science.
This journal is © The Royal Society of Chemistry 2024

Electronic Supplementary Information for

Influence of the substitution position on spin communication in photoexcited perylene–nitroxide dyads

Philipp Thielert,[1,†] Mélissa El Bitar Nehme,[2,†] Maximilian Mayländer,[1] Michael Franz,[1] Simon L. Zimmermann,[3] Fabienne Fisch,[2] Peter Gilch,[3] Andreas Vargas Jentzsch,[4] Michel Rickhaus,[2,5]* Sabine Richert[1]*

[1] *Institute of Physical Chemistry, University of Freiburg, Albertstraße 21, 79104 Freiburg, Germany*
[2] *Department of Chemistry, University of Zurich, Winterthurerstrasse 190, 8057 Zurich, Switzerland*
[3] *Institute of Physical Chemistry, Heinrich Heine University Düsseldorf, Universitätsstraße 1, 40225 Düsseldorf, Germany*
[4] *SAMS Research Group, Université de Strasbourg, CNRS, Institut Charles Sadron UPR 22, 67000 Strasbourg, France*
[5] *Department of Organic Chemistry, University of Geneva, 30 Quai Ernest-Ansermet, 1211 Geneva 4, Switzerland*

* E-mail: michel.rickhaus@unige.ch, sabine.richert@physchem.uni-freiburg.de
† These authors contributed equally.

Table of Contents

1 Synthetic procedures **S1**
1.1 General methods S2
1.2 Synthetic protocols S3
1.3 Variable temperature NMR studies S9

2 Steady state and time-resolved UV-vis spectroscopy **S21**
2.1 UV-vis spectroscopy S21
2.2 Calculation of the radiative rate constant using the Strickler-Berg equation S23
2.3 Calculation of the Förster resonance energy transfer rate constants S23
2.4 Femtosecond TA data acquisition S25
2.5 Determination of the triplet yields S26
2.6 Additional fsTA data and global kinetic analysis S26

3 EPR characterisation **S30**
3.1 EPR sample preparation S30
3.2 EPR setup and parameters S30
3.3 EPR characterisation of the radical and triplet precursors S31
3.4 Simulation of the trEPR spectra S33
3.5 Additional pulse EPR data S34

4 **Quantum chemical calculations** **S37**
4.1 Optimisation of the structures S37
4.2 Transition dipole moments of bromoperylenes S37
4.3 Rotational conformers and barriers S37
4.4 Excited state exchange coupling calculations S39

5 **NMR spectra** **S41**

6 **HRMS data** **S63**

List of Figures

S1 Procedure for the synthesis of the bromoperylenes S1
S2 Procedure for the synthesis of the perylene–*e*TEMPO isomers S2
S3 NMR low temperature calibration for per–1-*e*TEMPOH S10
S4 NMR high temperature calibration for per–1-*e*TEMPOH S11
S5 NMR low temperature calibration for per–2-*e*TEMPOH S12
S6 Variable temperature ^{1}H NMR spectra of per–1-*e*TEMPOH S12
S7 Variable temperature ^{1}H NMR spectra of per–1-*e*TEMPOH (aliphatic region) S13
S8 Variable temperature ^{1}H NMR spectra of per–2-*e*TEMPOH S13
S9 Variable temperature ^{1}H NMR spectra of per–2-*e*TEMPOH (aliphatic region) S14
S10 Determination of the coalescence temperature for per–1-*e*TEMPOH S15
S11 Determination of the coalescence temperature for per–1-*e*TEMPOH S16
S12 Determination of the coalescence temperature for per–2-*e*TEMPOH S16
S13 NMR lineshape analysis for per–1-*e*TEMPOH S17
S14 NMR lineshape analysis for per–2-*e*TEMPOH S18
S15 Eyring plot for per–1-*e*TEMPOH S19
S16 Eyring plot for per–2-*e*TEMPOH S20
S17 UV-vis absorption and fluorescence spectra of perylene S21
S18 UV-vis absorption and fluorescence spectra of the perylene–*e*TEMPO dyads S22
S19 Fluorescence decay trace of perylene S22
S20 Fluorescence decay traces of the bromoperylenes S23
S21 Spectral overlap of chromophore emission and radical absorption S24
S22 Contour plot of the fsTA data for perylene S26
S23 Decay associated spectra for the perylene–*e*TEMPO dyads S27
S24 Species associated spectra for per–1-*e*TEMPO S28
S25 Species associated spectra for per–2-*e*TEMPO S28
S26 Species associated spectra for per–2-*e*TEMPO S28
S27 Comparison of the cw EPR data for all investigated dyads S31
S28 EPR parameters of the *e*TEMPO radical S32
S29 Simulations of the trEPR data of the bromoperylene precursors S33
S30 Pulse EPR spectra of the perylene–*e*TEMPO compounds S34
S31 Transient nutation data for per–1-*e*TEMPO S35
S32 Spin relaxation data for per–1-*e*TEMPO in frozen toluene S35

S33 Spin relaxation data for per–2-*e*TEMPO in frozen toluene S36
S34 Spin relaxation data for per–3-*e*TEMPO in frozen toluene S36
S35 Optimised structures of the bromoperylenes S37
S36 Optimised structure of 1-bromoperylene S37
S37 Optimised structures of the perylene–*e*TEMPO dyads S38
S38 Optimised structures of per–2-*e*TEMPOH dyads S38
S39 Optimised structures of per–1-*e*TEMPOH dyads S39
S40 ^{1}H NMR of 2-Br S41
S41 ^{13}C NMR of 2-Br S41
S42 ^{1}H NMR of 1-Br S42
S43 ^{13}C NMR of 1-Br S42
S44 ^{13}C NMR comparison of perylene and 1-Br S43
S45 ^{1}H NMR of per–1-*e*TEMPO S43
S46 ^{1}H NMR of per–2-*e*TEMPO S44
S47 ^{1}H NMR of per–3-*e*TEMPO S44
S48 ^{1}H NMR of per–1-*e*TEMPOH S45
S49 ^{1}H NMR of per–2-*e*TEMPOH S45
S50 ^{13}C NMR of per–2-*e*TEMPOH S46
S51 ^{1}H-^{1}H COSY of per–1-*e*TEMPOH S47
S52 ^{1}H-^{1}H COSY of per–1-*e*TEMPOH (aromatic region) S48
S53 ^{1}H-^{1}H NOESY of per–1-*e*TEMPOH S49
S54 ^{1}H-^{13}C HSQC of per–1-*e*TEMPOH S50
S55 ^{1}H-^{13}C HSQC of per–1-*e*TEMPOH (aromatic region) S51
S56 ^{1}H-^{13}C HSQC-TOCSY of per–1-*e*TEMPOH (aromatic region) S52
S57 ^{1}H-^{13}C HMBC of per–1-*e*TEMPOH S53
S58 ^{1}H-^{13}C HMBC of per–1-*e*TEMPOH (aromatic region) S54
S59 ^{1}H-^{1}H COSY of per–2-*e*TEMPOH S55
S60 ^{1}H-^{1}H COSY of per–2-*e*TEMPOH (aromatic region) S56
S61 ^{1}H-^{1}H NOESY of per–2-*e*TEMPOH S57
S62 ^{1}H-^{13}C HSQC of per–2-*e*TEMPOH S58
S63 ^{1}H-^{13}C HSQC of per–2-*e*TEMPOH (aromatic region) S59
S64 ^{1}H-^{13}C HSQC-TOCSY of per–2-*e*TEMPOH (aromatic region) S60
S65 ^{1}H-^{13}C HMBC of per–2-*e*TEMPOH S61
S66 ^{1}H-^{13}C HMBC of per–2-*e*TEMPOH (aromatic region) S62
S67 (+)-HR-APCI-MS of 1-Br S63
S68 HR-EI-MS of 2-Br S63
S69 (+)-HR-ESI-MS of per–1-*e*TEMPO S64
S70 (+)-HR-ESI-MS of per–2-*e*TEMPO S64
S71 HR-ESI-MS of per–3-*e*TEMPO S65
S72 (+)-HR-ESI-MS of per–1-*e*TEMPOH S66
S73 (+)-HR-ESI-MS of per–2-*e*TEMPOH S66

1 Synthetic procedures

Overviews of the general procedures employed for the synthesis of the bromoperylene and perylene–*e*TEMPO isomers are shown below in Figures S1 and S2.

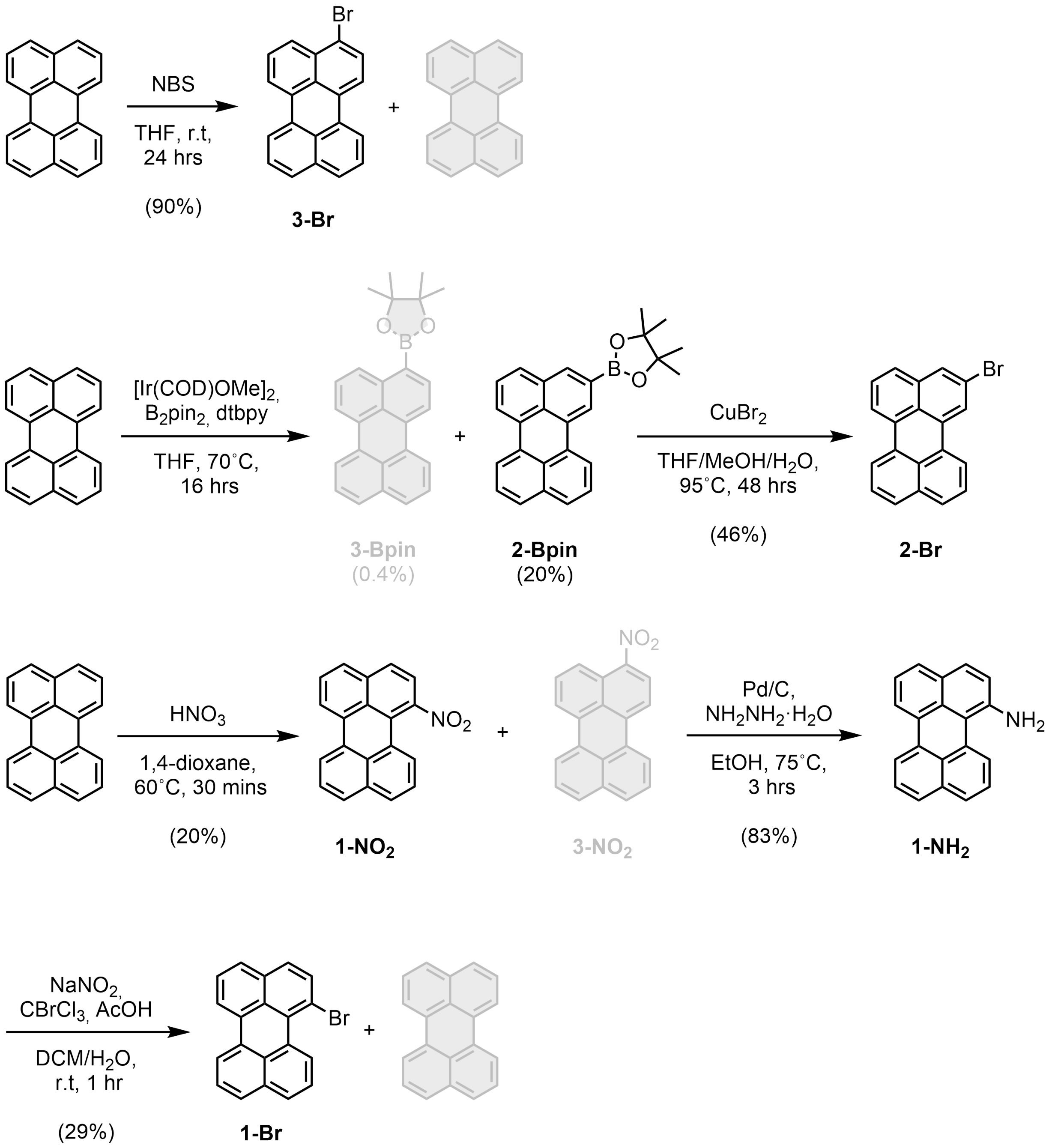

Figure S1: Overview of the procedures employed for the synthesis of the three bromoperylene isomers.

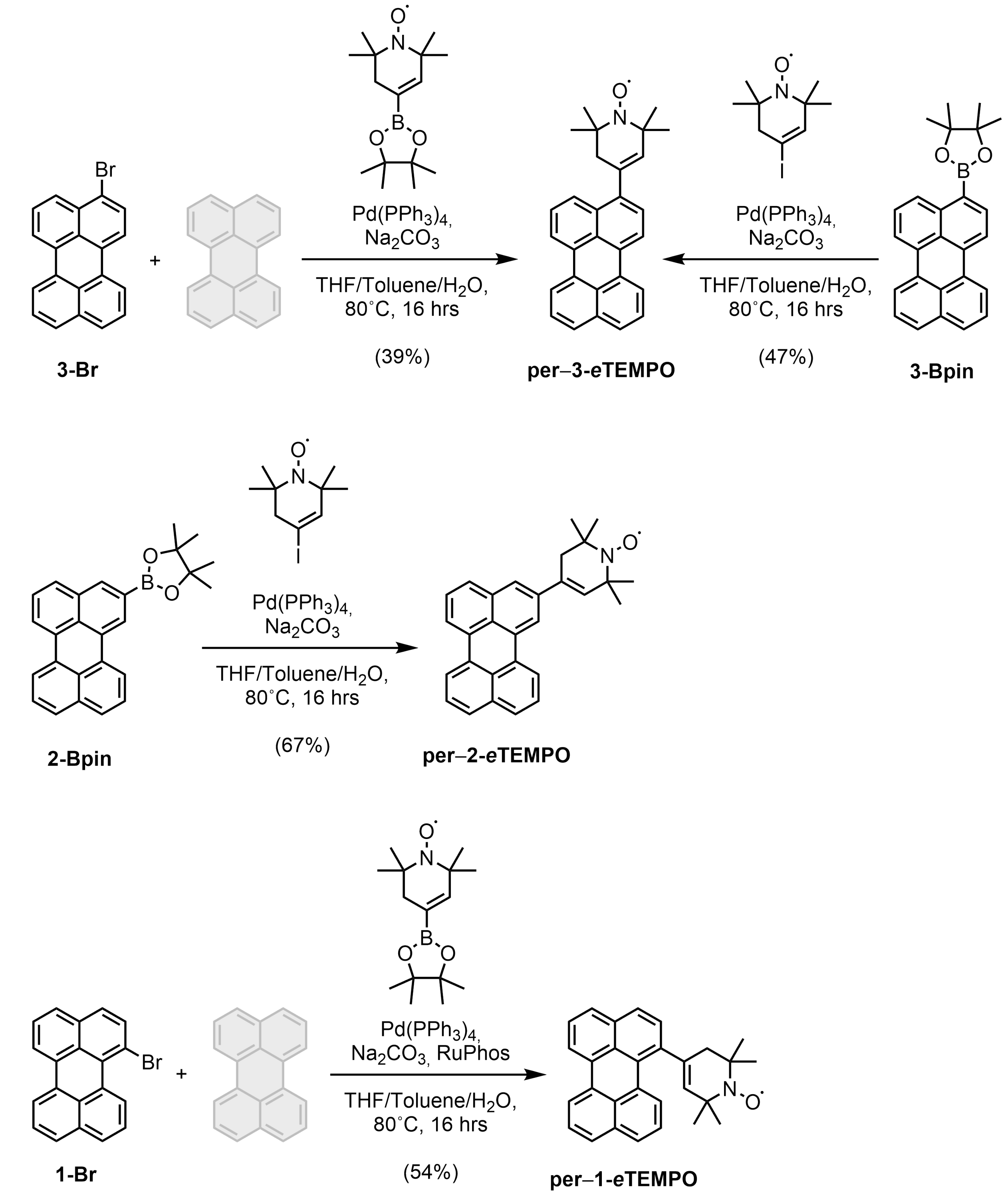

Figure S2: Overview of the procedures employed for the synthesis of the perylene–*e*TEMPO isomers.

1.1 General methods

All reagents and solvents were purchased from Avantor, Chemie Brunschwig AG, Sigma-Aldrich, or Thermo Fisher and used without further purification. Anhydrous solvents were obtained using a solvent purification system (Pure Solv PS-MD-4EN, Innovative Technology Inc.) equipped with alumina drying columns under argon. Reaction control was performed using analytical thin layer chromatography (TLC) on aluminum sheets coated with silica gel 60 F254 (Merck) or gas chromatography–mass spectrometry (GCMS) (Shimadzu Gas) Chromatograph GC-2010 Plus, GCMS-QP2010 SE. Visualisation of TLC plates

was achieved using UV light at 254 or 366 nm. Flash column chromatography (FC) was performed using SiO_2 (60 Å, 230–400 mesh, particle size 0.063–0.200 mm).

All **NMR spectra** were recorded on AV2-400, AV2-500 or AV-600 MHz Bruker spectrometers at 298 K unless stated otherwise. Chemical shifts are given in ppm and the spectra were calibrated using the residual chloroform signals (7.26 ppm for ^{1}H NMR and 77.16 ppm for ^{13}C NMR), and the residual tetrahydrofuran signals (3.58 ppm for ^{1}H NMR and 67.57 ppm for ^{13}C NMR). Coupling constants J are given in Hz and multiplicities are abbreviated as follows: s (singlet), d (doublet), t (triplet), m (multiplet), br (broad).

High resolution mass spectra (HR-MS) were recorded by the Mass Spectrometric Service at the University of Zurich on a Dionex Ultimate 3000 UHPLC system (ThermoFischer Scientifics, Germering, Germany) connected to a QExactive MS with a heated ESI source (ThermoFisher Scientific, Bremen, Germany) or on a double-focusing (BE geometry) magnetic sector mass spectrometer DFS (ThermoFisher Scientific, Bremen, Germany) with a heated EI source.

1.2 Synthetic protocols

Compounds 3-Br,[1] 3-Bpin (obtained as a side product of perylene borylation but can also be synthesised from the borylation of 3-Br[2]), 2-Bpin,[3] 2-Br,[3] 1-NO_2,[4] and 1-NH_2[4] were synthesised according to previously reported literature procedures. The analytical data were in agreement with those reported in the literature.

Compound 3-Br

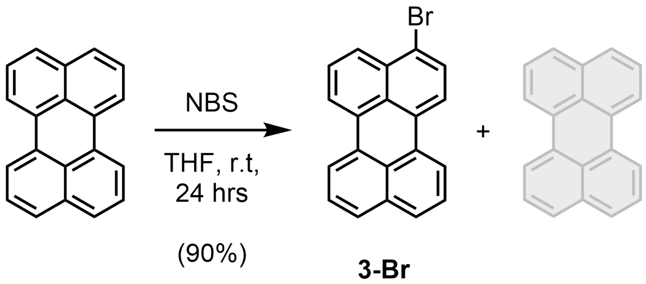

A solution of N-bromosuccinimide (NBS) (352 mg, 1.98 mmol, 1.0 equiv.) in dry THF (25 mL) was added to a solution of perylene (500 mg, 1.98 mmol, 1.0 equiv.) in dry THF (25 mL) and stirred at room temperature under nitrogen atmosphere for 24 h. The mixture was poured into water (75 mL) and extracted with dichloromethane (3×25 mL). The organic layer was dried over anhydrous $MgSO_4$. After filtration, the solvent was removed under reduced pressure to afford compound 3-Br (655 mg, 90 % + 10 % perylene) with unreacted perylene as a side product (≈ 10 % by NMR). The spectroscopic characterisation was consistent with the reported data.[1]

Note: Increasing the amount of brominating agent (NBS) could potentially lead to the formation of isomeric dibromoperylenes which would make the purification difficult. Efforts to isolate pure 3-Br using column chromatography and sublimation were unsuccessful due to the limited solubility of perylene and the compounds' closely matched R_f values in the former method, while the latter approach resulted in the degradation of the crude mixture. An analytically pure sample of 3-Br could, however, be obtained by recrystallisation and was used for the spectroscopic and photophysical characterisation.

^{1}**H NMR** (400 MHz, $CDCl_3$) δ = 8.27–8.13 (m, 3H), 8.09 (dd, J = 8.5, 1.0 Hz, 1H), 8.00 (d, J = 8.1 Hz, 1H), 7.77 (d, J = 8.1 Hz, 1H), 7.71 (dt, J = 8.3, 1.2 Hz, 2H), 7.58 (dd, J = 8.4, 7.5 Hz, 1H), 7.49 (td, J = 7.7, 4.7 Hz, 2H).

Compounds 2-Bpin and 3-Bpin

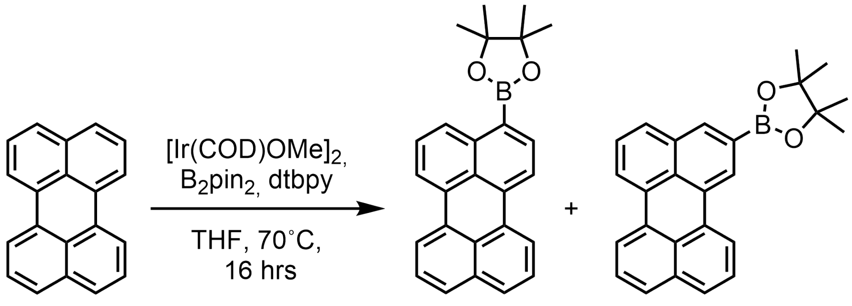

3-Bpin (0.4%) **2-Bpin** (20%)

A Schlenk flask was charged with perylene (1.00 g, 3.96 mmol, 1.0 equiv.), B_2pin_2 (1.00 mg, 3.96 mmol, 1.0 equiv.), $[Ir(OMe)COD]_2$ (39.4 mg, 0.0594 mmol, 0.015 equiv.) and dtbpy (31.9 mg, 0.119 mmol, 0.03 equiv.). THF (40 mL) was added under nitrogen and the reaction was stirred at 70°C for 16 h, after which the solvent was removed, and the crude mixture was purified by column chromatography (SiO_2; Hex/DCM = 7:3) to yield the desired compound 2-Bpin as an orange solid (305.5 mg, 20 %). 3-Bpin was obtained as a yellow solid (6.1 mg, 0.4 %). The spectroscopic characterisation was consistent with the reported data.[2,3]

^{1}H NMR, 2-Bpin (400 MHz , $CDCl_3$) δ = 8.56 (d, J = 1.1 Hz, 1H), 8.33 (dd, J = 7.6, 1.1 Hz, 1H), 8.21 (dd, J = 7.6, 1.1 Hz, 1H), 8.19 (s, 1H), 8.20–8.14 (m, 1H), 7.72 (dd, J = 8.3, 1.0 Hz, 1H), 7.68 (d, J = 1.9 Hz, 1H), 7.66 (d, J = 2.0 Hz, 1H), 7.50–7.43 (m, 3H), 1.43 (s, 12H).

^{1}H NMR, 3-Bpin (400 MHz, $CDCl_3$) δ = 8.68–8.64 (m, 1H), 8.26–8.16 (m, 4H), 8.06 (d, J = 7.6 Hz, 1H), 7.69 (dd, J = 11.3, 8.1 Hz, 2H), 7.58–7.44 (m, 3H), 1.44 (s, 12H).

Compound 2-Br

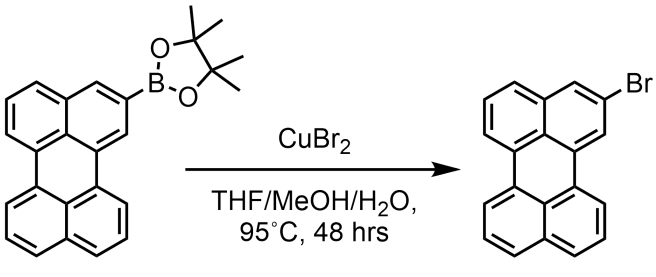

2-Bpin (46%) **2-Br**

2-Bpin (49.9 mg, 0.132 mmol, 1.0 equiv.) was dissolved in 2 mL of THF and heated to 50°C. Then, 2 mL of MeOH was added to this solution followed by $CuBr_2$ (88.4 mg, 0.396 mmol, 3.0 equiv.) dissolved in 2 mL of H_2O. The mixture was stirred at 95°C for 48 h until reaction monitoring by TLC showed that the reaction was complete. The solution was diluted with water and extracted with DCM (3×50 mL). The organic phases were collected, and the solvent was removed under reduced pressure. The crude product was purified by column chromatography (SiO_2; Hexane) to give 2-Br as a yellow solid (20 mg, 46 %).

^{1}H NMR (400 MHz, $CDCl_3$) δ = 8.22 (d, J = 1.8 Hz, 1H), 8.21–8.13 (m, 3H), 7.81 (d, J = 1.8 Hz, 1H), 7.71 (t, J = 7.7 Hz, 2H), 7.57 (d, J = 8.1 Hz, 1H), 7.49 (td, J = 7.9, 2.5 Hz, 3H).

^{13}C NMR (101 MHz, $CDCl_3$) δ = 136.08, 134.71, 133.56, 131.57, 130.70, 129.95, 129.40, 128.84, 128.65, 128.36, 127.63, 127.39, 126.86, 126.73, 126.61, 123.30, 121.13, 121.01, 120.76, 120.41.

EI-HRMS, m/z = 330.00378 ($C_{20}H_{11}Br$, $[M]^+$ requires 330.00386).

Compound 1-NO_2

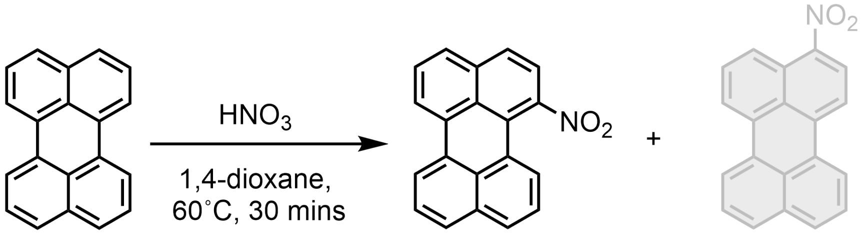

1-NO_2 (20%) **3-NO_2**

Perylene (500 mg, 1.98 mmol, 1.0 equiv.) was added in dry 1,4-dioxane (40 mL), then the mixture was stirred and heated to 85°C until it was completely dissolved. Concentrated HNO_3 (2 mL, 60–80 %) was added dropwise at 60°C. After stirring for 30 minutes, the reaction mixture was poured into a large amount of ice water and filtered under reduced pressure to get a red crude product which was purified by column chromatography (SiO_2; DCM/PE = 2:3) to give the desired compound 1-NO_2 (117 mg, 20 %). The low yield in this step is probably due to the low solubility of the crude mixture. The spectroscopic characterisation was consistent with the reported data.[4]

^{1}**H NMR**, 1-NO_2 (400 MHz, $CDCl_3$) δ = 8.22 (ddd, J = 9.8, 7.5, 1.1 Hz, 2H), 7.83–7.75 (m, 3H), 7.72–7.67 (m, 2H), 7.60 (t, J = 7.8 Hz, 1H), 7.56–7.51 (m, 2H), 7.43 (t, J = 7.9 Hz, 1H).

Compound 1-NH_2

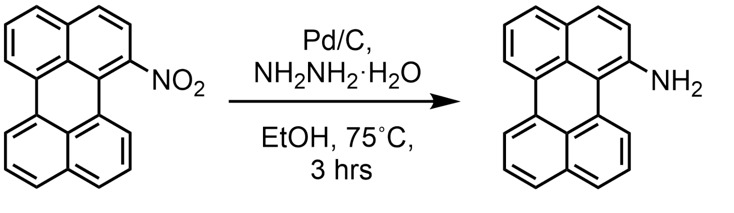

1-NO_2 (83%) **1-NH_2**

Under nitrogen atmosphere, 1-NO_2 (90.1 mg, 0.303 mmol, 1.0 equiv.), 10 % Pd/C (79 mg, 0.742 mmol, 2.4 equiv.), $NH_2NH_2 \cdot H_2O$ (2.25 mL) were added in ethanol (70 mL), then the mixture was refluxed and stirred for 3 h under nitrogen atmosphere. After TLC monitoring showed that the reaction was complete, the mixture was filtered under reduced pressure to give an orange solution. Then the solvent was removed under reduced pressure to give compound 1-NH_2 as an orange solid (67.5 mg, 83 %). The compound was used in the next step without further purification. The spectroscopic characterisation was consistent with the reported data.[4]

^{1}**H NMR** (400 MHz, DMSO-d_6) δ = 8.42 (dd, J = 7.6, 1.1 Hz, 1H), 8.15 (ddd, J = 7.4, 6.0, 1.2 Hz, 2H), 7.68 (dd, J = 8.2, 1.1 Hz, 1H), 7.62–7.53 (m, 3H), 7.47 (dt, J = 9.3, 7.8 Hz, 2H), 7.26 (t, J = 7.7 Hz, 1H), 7.19 (d, J = 8.8 Hz, 1H), 6.06 (s, 2H).

Compound 1-Br

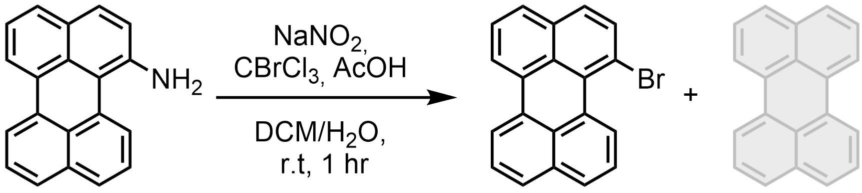

1-NH_2 (29%) **1-Br**

Compound 1-NH_2 (246.5 mg, 0.922 mmol, 1.0 equiv.), $NaNO_2$ (318 mg, 4.61 mmol, 5 equiv.), $BrCCl_3$ (0.183 mL, 1.84 mmol, 2 equiv.), and solvent system (5 mL each of DCM and H_2O) were stirred at room temperature for 5 minutes. HOAc (1.05 mL, 18.4 mmol, 20 equiv.) was then added in one portion and the reaction was stirred under nitrogen for 1 h. After TLC monitoring showed the completion of the reaction, the organic layer was separated and the aqueous layer was extracted with DCM (3×20 mL). The combined organic layers were washed with brine, dried over magnesium sulfate, and concentrated under reduced pressure. The crude compound was purified by column chromatography (SiO_2; Hexane). 1-Br was obtained as a yellow solid (89.2 mg, 29 %).

Note: Perylene was obtained as a side product. The mechanism proposed in the literature [5] suggests that the reaction follows a free radical mechanism arising from the diazonium cation's homolytic cleavage. This accounts for the substantial quantity of perylene produced also leading to a reduced overall yield. The aryl free radical subsequently reacts with $CBrCl_3$, resulting in the formation of 1-Br, Cl_3C-OAc, and Cl_3C-CCl_3.

^{1}H NMR (400 MHz, $CDCl_3$) δ = 9.22 (dd, J = 7.7, 1.0 Hz, 1H), 8.18 (ddd, J = 7.5, 3.9, 1.2 Hz, 2H), 7.80–7.76 (m, 2H), 7.75 (d, J = 8.7 Hz, 1H), 7.69 (dd, J = 8.1, 1.2 Hz, 1H), 7.58–7.48 (m, 4H).

^{13}C NMR (101 MHz, $CDCl_3$) δ = 134.59, 134.06, 133.40, 131.02, 130.88, 130.39, 130.36, 129.76, 129.57, 128.67, 128.11, 128.01, 127.48, 126.91, 126.75, 126.43, 125.71, 121.42, 120.63, 118.18.

(+)-HR-APCI-MS, m/z = 331.01198 ($C_{20}H_{12}Br$, $[M+H]^+$ requires 331.01169).

Compound per–3-*e*TEMPO: Route A

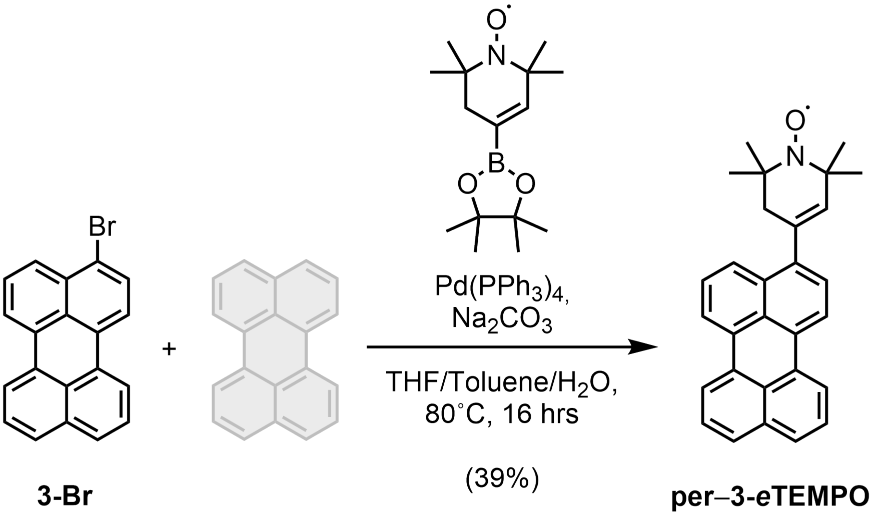

3-Br (39%) **per–3-*e*TEMPO**

In a 50 mL Schlenk flask, 3-Br (15.0 mg, 0.0453 mmol, 1.0 equiv.), Bpin–*e*TEMPO (21.2 mg, 0.0757 mmol, 1.67 equiv.) and Na_2CO_3 (15.1 mg, 0.142 mmol, 3.14 equiv.) were dissolved in

18 mL of water/THF/toluene (1:1:1). Tetrakis(triphenylphosphine) palladium (3.19 mg, 0.00276 mmol, 0.0609 equiv.) was added and the reaction mixture was stirred at 80°C for 16 h (monitored by TLC). After reaction completion, a saturated solution of Na_2CO_3 (15 mL) was added and the mixture was extracted with DCM (3×50 mL). The combined organic extracts were dried over Na_2SO_4 and filtered. The solvent was removed under reduced pressure, and the compound was purified by column chromatography (SiO_2; DCM/Hex = 7:3 → DCM) to give compound per–3-*e*TEMPO (7.2 mg, 39 %) as a yellow solid.

^{1}H NMR (400 MHz, $CDCl_3$) δ = 8.34–8.27 (m, 2H), 8.24 (s, 1H), 8.10 (s, 1H), 7.79 (t, J = 8.3 Hz, 2H), 7.61 (s, 1H), 7.53 (d, J = 8.2 Hz, 2H).

HR-ESI-MS, m/z = 404.20067 ($C_{29}H_{26}NO^{\bullet}$, $[M]^+$ requires 404.20089).

Compound per–3-*e*TEMPO: Route B

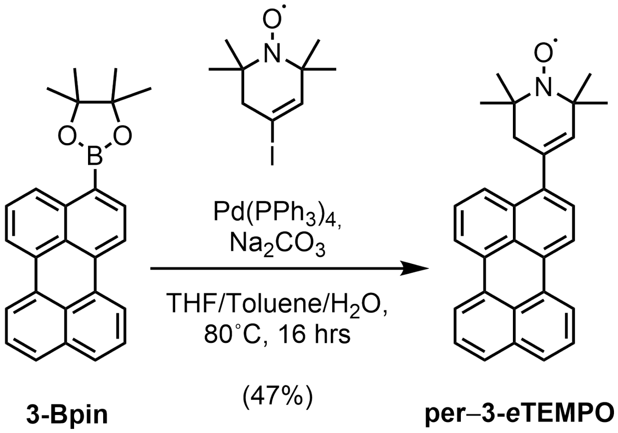

3-Bpin **per–3-*e*TEMPO**

In a 50 mL Schlenk flask, 3-Bpin (12.1 mg, 0.0321 mmol, 1.0 equiv.), I–*e*TEMPO (15.0 mg, 0.0535 mmol, 1.67 equiv.) and Na_2CO_3 (10.7 mg, 0.101 mmol, 3.14 equiv.) were dissolved in 18 mL of water/THF/toluene (1:1:1). Tetrakis(triphenylphosphine) palladium (2.26 mg, 0.00195 mmol, 0.0609 equiv.) was added and the reaction mixture was stirred at 80°C for 16 h (monitored by TLC). After reaction completion, a saturated solution of Na_2CO_3 (15 mL) was added and the mixture was extracted with DCM (3×50 mL). The combined organic extracts were dried over Na_2SO_4 and filtered. The solvent was removed under reduced pressure, and the compound was purified by column chromatography (SiO_2; DCM/Hex = 7:3 → DCM) to give compound per–3-*e*TEMPO (6.1 mg, 47 %) as a yellow solid.

Compound per–2-*e*TEMPO

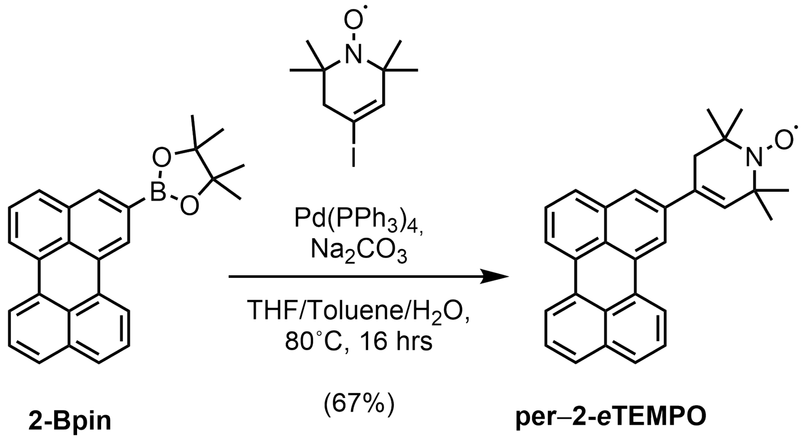

2-Bpin **per–2-*e*TEMPO**

In a 50 mL Schlenk flask, 2-Bpin (16.0 mg, 0.0423 mmol, 1.0 equiv.), I–*e*TEMPO (19.8 mg, 0.0706 mmol, 1.67 equiv.) and Na_2CO_3 (14.1 mg, 0.133 mmol, 3.14 equiv.) were dissolved in 18 mL of water/THF/toluene (1:1:1). Tetrakis(triphenylphosphine)palladium (2.98 mg, 0.00258 mmol, 0.0609 equiv.) was added and the reaction mixture was stirred at 80°C for 16 h (monitoring by TLC). After reaction completion, a saturated solution of Na_2CO_3 (15 mL) was added and the mixture extracted with DCM (3×50 mL). The combined organic extracts were dried over Na_2SO_4 and filtered. The solvent was removed under reduced pressure, and the compound was purified by column chromatography (SiO_2; DCM/Hex = 7:3 → DCM) to give compound per–2-*e*TEMPO (11.6 mg, 67 %) as a yellow solid.

^{1}**H NMR** (400 MHz, $CDCl_3$) δ = 8.40 (d, J = 6.2 Hz, 1H), 8.25 (s, 1H), 8.18 (d, J = 7.3 Hz, 1H), 7.88 (s, 1H), 7.69 (d, J = 7.8 Hz, 2H), 7.59–7.51 (m, 2H), 7.45 (d, J = 7.8 Hz, 1H).

(+)-HR-ESI-MS, m/z = 404.20158 ($C_{29}H_{26}NO^{\bullet}$, $[M]^+$ requires 404.20089).

Compound per–1-*e*TEMPO

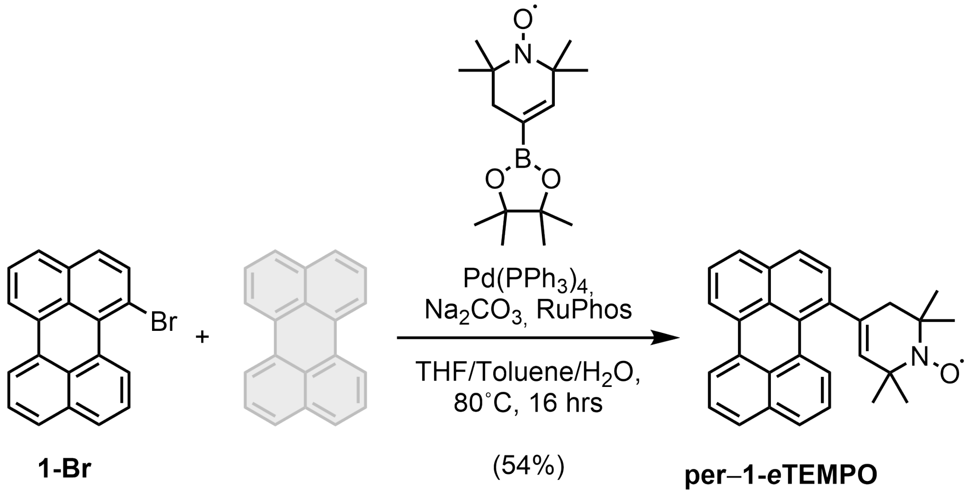

In a 50 mL Schlenk flask, 1-Br (11.7 mg, 0.0353 mmol, 1.1 equiv.), Bpin–*e*TEMPO (9.0 mg, 0.0321 mmol, 1.0 equiv.) and Na_2CO_3 (10.2 mg, 0.0963 mmol, 3.0 equiv.) were dissolved in 18 mL of water/THF/toluene (1:1:1). Tetrakis(triphenylphosphine)palladium (3.71 mg, 0.00321 mmol, 0.1 equiv.) and RuPhos (1.58 mg, 0.00321 mmol, 0.1 equiv.) were added and the reaction mixture was stirred at 80°C for 16 h (monitoring by TLC). After reaction completion, a saturated solution of Na_2CO_3 (15 mL) was added and the mixture extracted with DCM (3×50 mL). The combined organic extracts were dried over Na_2SO_4 and filtered. The solvent was removed under reduced pressure, and the compound was purified by prepTLC (SiO_2; DCM) to give compound per–1-*e*TEMPO as a yellow solid (7.0 mg, 54 %).

^{1}**H NMR** (400 MHz, $CDCl_3$) δ = 8.29 (d, J = 7.1 Hz, 1H), 8.20 (d, J = 7.0 Hz, 1H), 7.84–7.76 (m, 2H), 7.70 (d, J = 7.6 Hz, 1H), 7.65–7.58 (m, 1H), 7.55 (d, J = 7.0 Hz, 2H).

(+)-HR-ESI-MS, m/z = 404.20125 ($C_{29}H_{26}NO^{\bullet}$, $[M]^+$ requires 404.20089).

1.3 Variable temperature NMR studies

Variable temperature NMR studies were carried out on the reduced versions of per–1-*e*TEMPO and per–2-*e*TEMPO to confirm the presence of conformers. These data can be used to extract the thermodynamic parameters associated with their thermal isomerisation using two different approaches: (a) the determination of the theoretical coalescence temperature, and (b) the analysis of the lineshape as describe in dynamic nuclear magnetic resonance spectroscopy. Both analysis have been carried out and are presented in the following.

General procedure for the reduction of perylene–*e*TEMPO

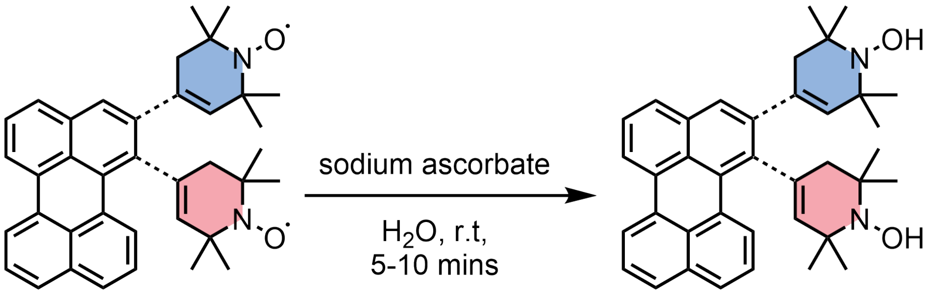

per–1-*e*TEMPO **per–1-*e*TEMPOH**

per–2-*e*TEMPO **per–2-*e*TEMPOH**

Following a modified procedure reported by Tidwell,[6] a solution of sodium ascorbate (1.18 mg, 0.00593 mmol, 1.2 equiv.) in D_2O (0.1–0.2 mL) was added to a solution of perylene–*e*TEMPO (2.0 mg, 0.00494 mmol, 1.0 equiv.) in THF-d_8 (0.5 mL) in an NMR tube. The mixture was sonicated for 2-3 minutes using a vortex mixer after which a ^{1}H NMR was measured and showed the formation of perylene–*e*TEMPOH.

^{1}H NMR, per–1-*e*TEMPOH (600 MHz, THF-d_8) δ = 8.42 (s, 1H), 8.17 (d, J = 6.8 Hz, 2H), 7.69–7.58 (m, 4H), 7.44 (td, J = 7.6, 5.7 Hz, 2H), 7.39–7.35 (m, 1H), 7.24–7.18 (m, 1H), 5.61 (s, 1H), 2.50 (s, 1H), 1.94 (s, 1H), 1.26 (s, 6H), 1.18 (s, 3H), 1.07 (s, 3H).

^{13}C NMR, per–1-*e*TEMPOH (151 MHz, THF-d_8) δ = 140.05, 138.03, 135.54, 134.96, 134.63, 132.39, 132.31, 131.95, 131.86, 130.86, 130.41, 129.26, 128.94, 128.78, 128.71, 128.58, 128.35, 127.73, 127.59, 127.32, 122.19, 121.37, 43.40, 26.60, 26.05.
The ^{13}C NMR peaks were deduced from 2D-NMR measurements since a proper ^{13}C NMR could not be recorded. The $\underline{C}(CH_3)_2$ could not be assigned.

(+)-HR-ESI-MS, per–1-*e*TEMPOH, m/z = 406.21676 ($C_{29}H_{28}NO$, $[M+H]^+$ requires 406.21654).

^{1}H NMR, per–2-*e*TEMPOH (600 MHz, THF-d_8) δ = 8.33 (d, J = 1.7 Hz, 1H), 8.30 (dd, J = 7.7, 1.0 Hz, 1H), 8.23 (dd, J = 7.6, 1.0 Hz, 1H), 8.19 (dd, J = 7.6, 1.0 Hz, 1H), 7.69–7.64 (m, 3H), 7.63 (d, J = 1.6 Hz, 1H), 7.47–7.42 (m, 3H), 6.15 (s, 1H), 2.60 (s, 2H), 1.31 (s, 6H), 1.23 (s, 6H).

^{13}C NMR, per–2-*e*TEMPOH (151 MHz, THF-d_8) δ = 139.89, 135.99, 135.91, 133.76, 131.92, 131.91, 131.86, 131.84, 130.89, 129.63, 128.90, 128.78, 128.77, 128.61, 127.87, 127.53, 127.41, 124.43, 121.24, 121.20, 120.92, 118.48, 60.45, 57.95, 42.85, 27.09, 25.81.

(+)-HR-ESI-MS, per–2-*e*TEMPOH, m/z = 406.21684 ($C_{29}H_{28}NO$, $[M+H]^+$ requires 406.21654).

NMR temperature calibration

Despite modern NMR spectrometers' temperature control, calibration is essential for reliability in variable temperature experiments. Temperature profoundly affects molecular dynamics, shifting peaks and altering the spectral appearance. This offers insights into thermodynamic parameters, relying on precise temperature measurement.

Methanol and ethylene glycol are commonly chosen calibration solvents for various temperature ranges. Methanol is utilised for lower temperatures (170–320 K), whereas ethylene glycol is used at higher temperatures (300–410 K). These standards generate a two-peak spectrum, distinguishing methyl and hydroxyl groups. With increasing temperature, hydrogen bonding decreases, and the hydroxyl peak shifts upfield towards the CH_3 or CH_2 resonances. Instead of measuring chemical shifts directly, the peak-to-peak distance is measured for temperature determination: Van Geet's technique involves gauging the temperature of liquid methanol by measuring the chemical shift difference ($\Delta\upsilon$, in Hz) between methyl and hydroxyl proton peaks in ^{1}H NMR spectra.[7] The Bruker software TopSpin offers the "calctemp" macro for peak identification, chemical shift difference measurement, and temperature reporting.

Low temperature calibration for per–1-*e*TEMPOH

For temperature calibration, a CH_3OH (4 %) in CD_3OD sample was used. The following equations were used to draw the subsequent calibration curve:

$$\begin{aligned} &\text{For } \Delta\delta \text{ 1.4965 to 1.76:} && \text{T [K]} = -114.83\,\Delta\delta + 471.85 \\ &\text{For } \Delta\delta \text{ 1.76 to 2.08:} && \text{T [K]} = -125\,\Delta\delta + 490 \\ &\text{For } \Delta\delta \text{ 2.08 to 2.43:} && \text{T [K]} = -140\,\Delta\delta + 521.33 \end{aligned}$$

where T is the absolute temperature in Kelvin and $\Delta\delta$ the chemical shift difference between CH_3 and OH resonances in CH_3OH (in ppm).[8]

Figure S3 shows the calibration curve together with its associated statistical errors. The temperatures utilised in the computation of thermodynamic information were adjusted to align with the calibrated temperature data. The following equation was then used to calculate the calibrated temperatures:

$$T_{\text{calibrated}}\,[\text{K}] = (1.107)\,T_{\text{set}} - 35.39$$

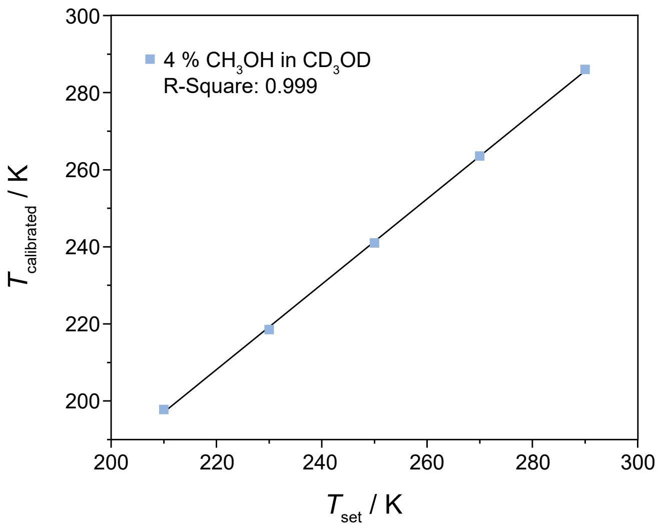

Figure S3: Temperature calibration for low temperature NMR experiments using a CH_3OH (4 %) in CD_3OD sample.

High temperature calibration for per–1-*e*TEMPOH

For temperature calibration, a $(CH_2OH)_2$ (80 %) in DMSO-d_6 sample was used. The following equation was used to draw the subsequent calibration curve:

$$T\ [K] = -108.33\,\Delta\delta + 460.41$$

where T is the absolute temperature in Kelvin and $\Delta\delta$ the chemical shift difference between CH_2 and OH resonances in $(CH_2OH)_2$ (in ppm).[8]

Figure S4 displays the calibration curve along with its associated statistical errors. The temperatures used in the computation of thermodynamic information were adjusted to align with the calibrated temperature data. The following equation was then used to calculate the calibrated temperatures:

$$T_{calibrated}\ [K] = (1.1012)\,T_{set} - 9.816$$

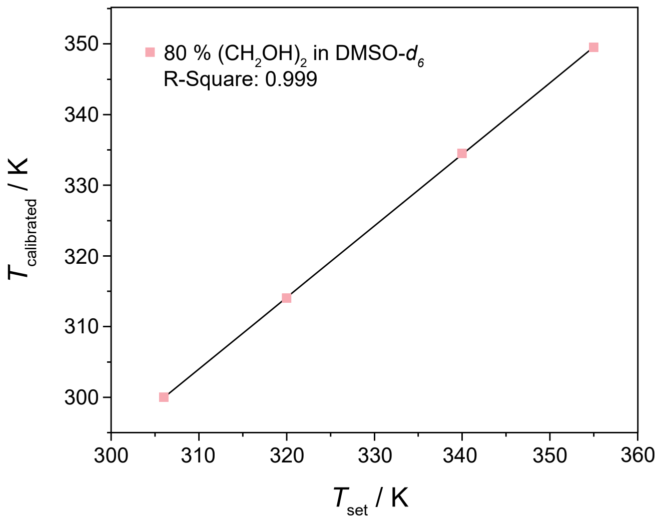

Figure S4: Temperature calibration for high temperature NMR experiments using a $(CH_2OH)_2$ (80 %) in DMSO-d_6 sample.

Low temperature calibration for per–2-*e*TEMPOH

For temperature calibration, a CH_3OH (4 %) in CD_3OD sample was used. The following equations were used to draw the subsequent calibration curve:

For $\Delta\delta$ 1.4965 to 1.76:	$T\ [K] = -114.83\,\Delta\delta + 471.85$
For $\Delta\delta$ 1.76 to 2.08:	$T\ [K] = -125\,\Delta\delta + 490$
For $\Delta\delta$ 2.08 to 2.43:	$T\ [K] = -140\Delta\delta + 521.33$

where T is the absolute temperature in Kelvin and $\Delta\delta$ the chemical shift difference between CH_3 and OH resonances in CH_3OH (in ppm).[8]

Figure S5 shows the calibration curve together with its associated statistical errors. The temperatures utilised in the computation of thermodynamic information were adjusted to align with the calibrated temperature data. The following equation was then used to calculate the calibrated temperatures:

$$T_{calibrated}\ [K] = (1.114)\,T_{set} - 34.52$$

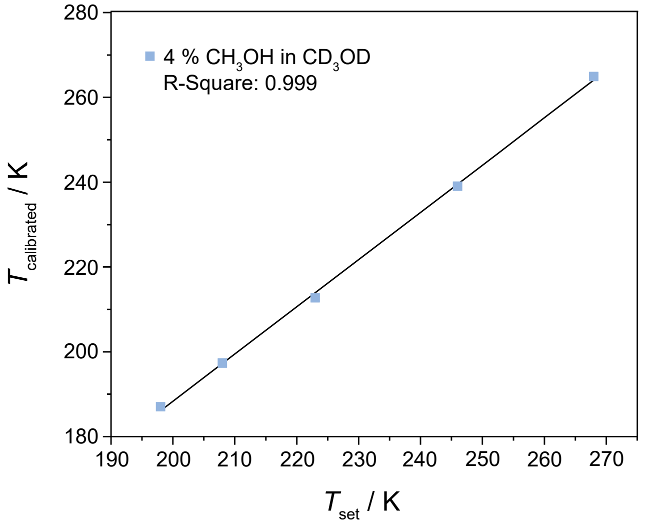

Figure S5: Temperature calibration for low temperature NMR experiments using a CH_3OH (4 %) in CD_3OD sample.

Variable temperature ^{1}H NMR spectra

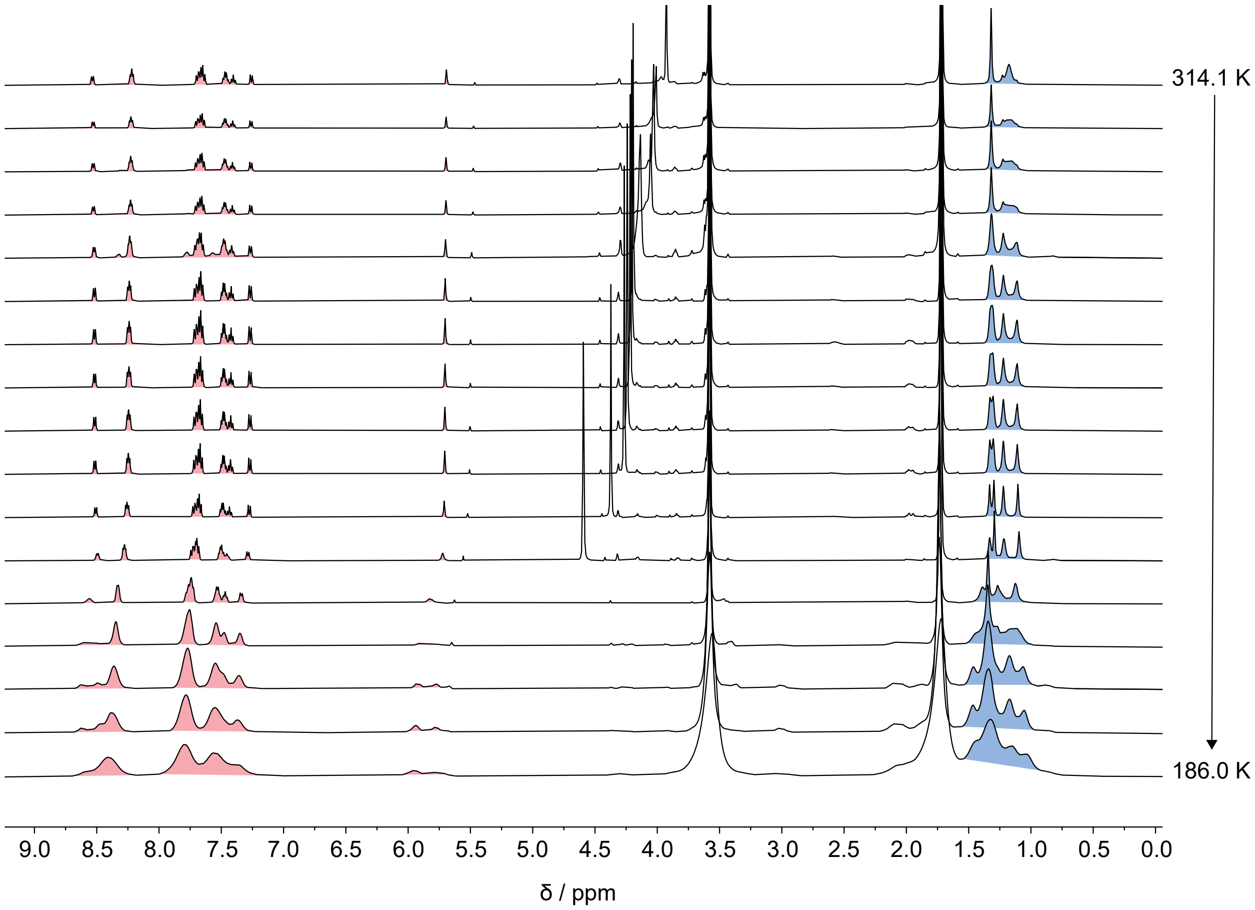

Figure S6: Experimental variable temperature ^{1}H NMR spectra of per–1-*e*TEMPOH (500 MHz, 314.1 → 186.0 K, THF-d_8).

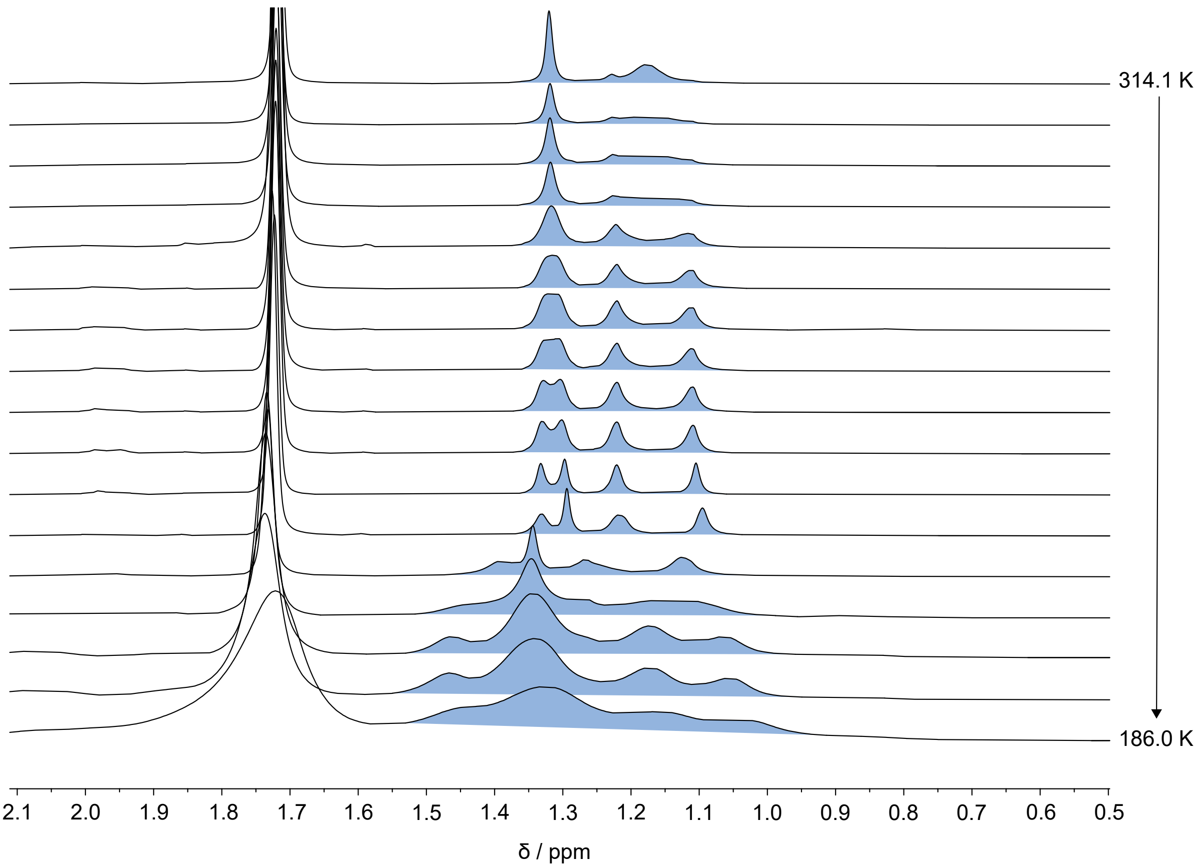

Figure S7: Experimental variable temperature ^{1}H NMR spectra of per–1-*e*TEMPOH (500 MHz, 314.1 → 186.0 K, THF-d_8, aliphatic region).

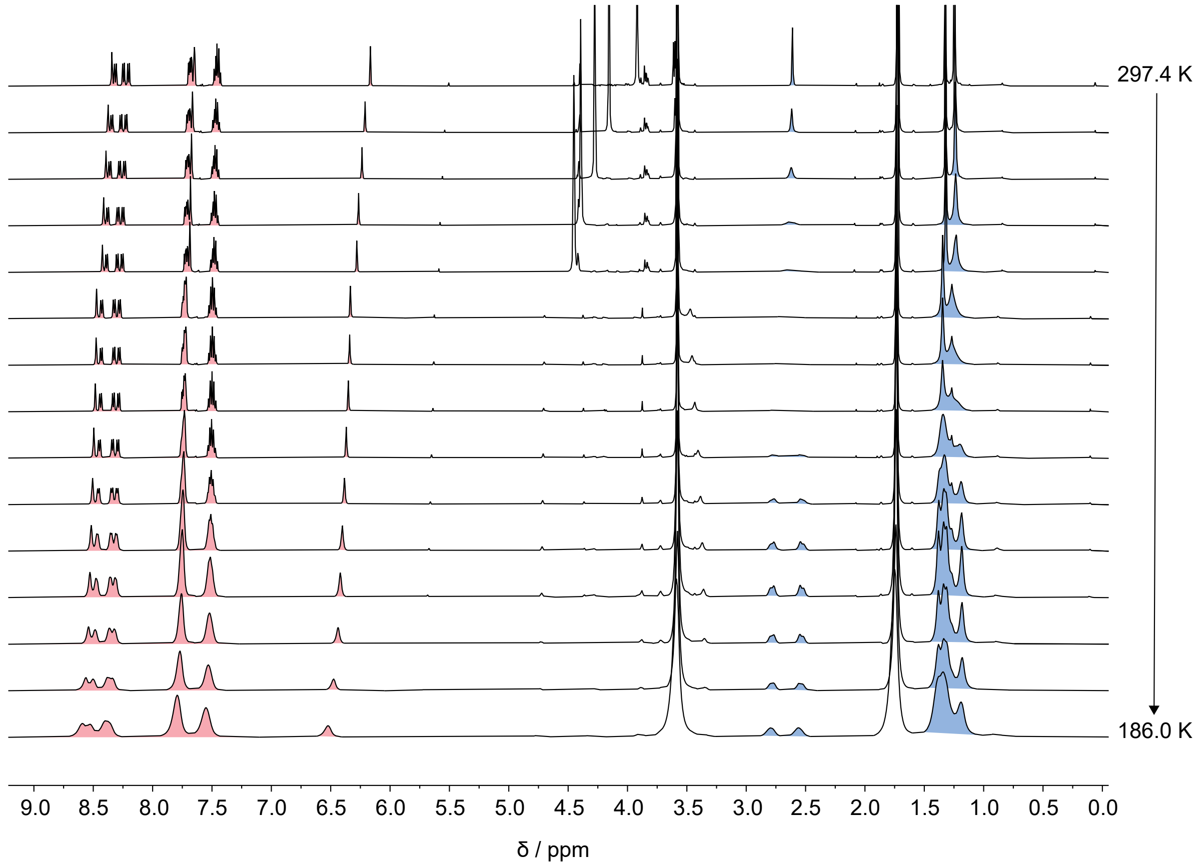

Figure S8: Experimental variable temperature ^{1}H NMR spectra of per–2-*e*TEMPOH (500 MHz, 297.4 → 186.0 K, THF-d_8).

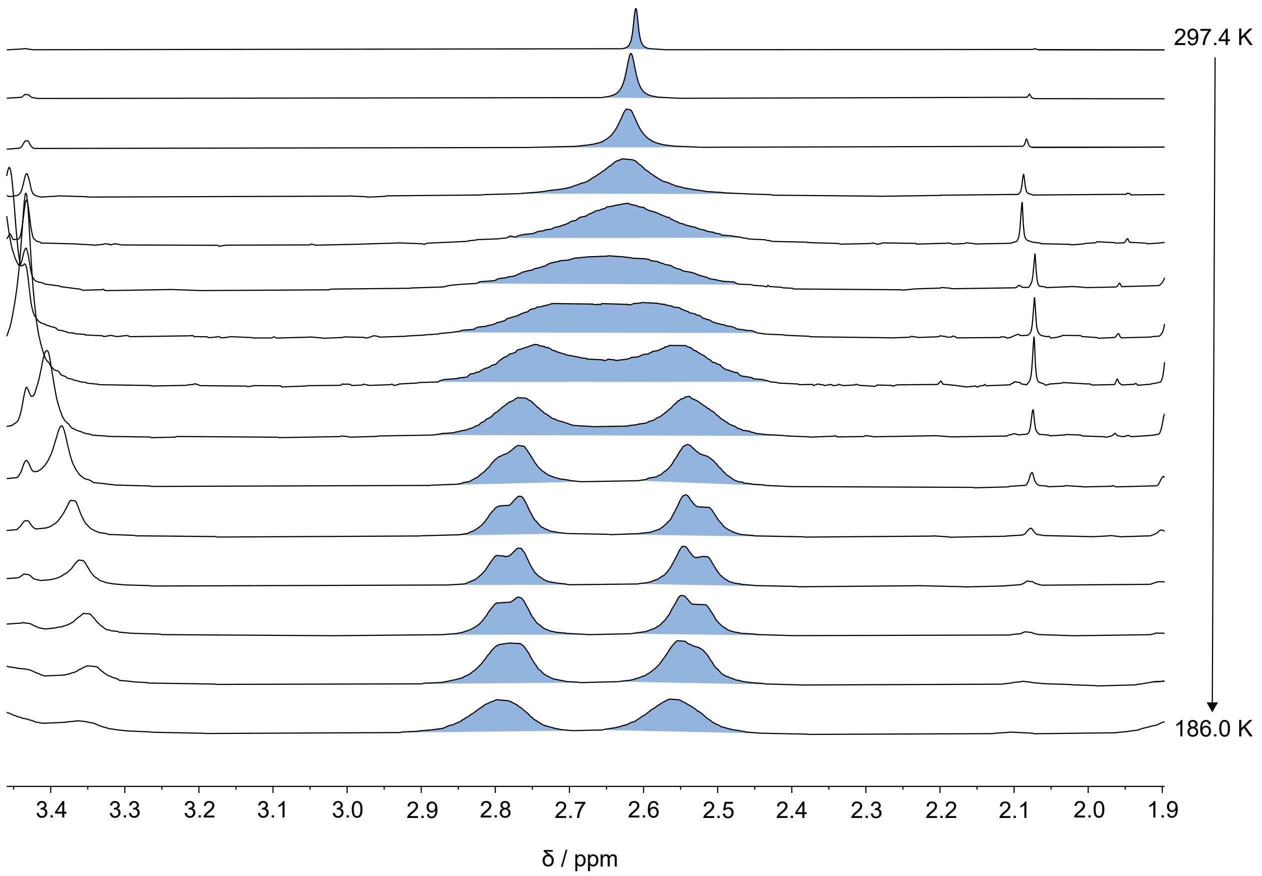

Figure S9: Experimental variable temperature ^{1}H NMR spectra of per–2-*e*TEMPOH (500 MHz, 297.4 → 186.0 K, THF-d_8, aliphatic region).

Determination of the theoretical coalescence temperature T_c

The coalescence temperatures were calculated by determining the full width at half maximum (FWHM) of the anisochronous signal. Subsequently, a plot is constructed, illustrating the relationship between the acquired linewidth values and the corresponding recorded internal temperatures. A Lorentzian fit is applied to the plotted data, aligning a Lorentzian function with the observed points. Through this fitting procedure, the temperature at which the maximum linewidth is attained can be determined, thereby establishing the theoretical coalescence temperature, T_c.[9] The corresponding data are shown below in Figures S10 to S12.

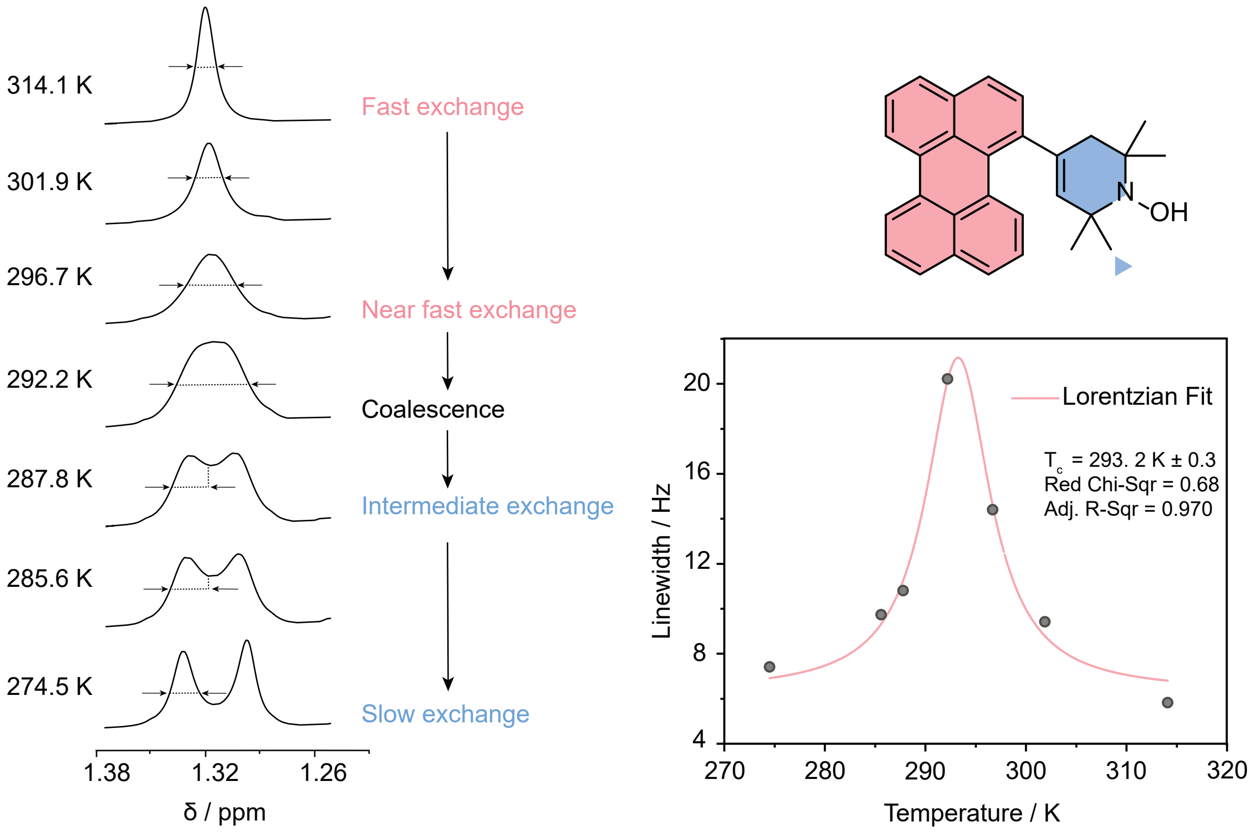

Figure S10: Process of determining the theoretical coalescence temperature, T_c, for per–1-*e*TEMPOH: the full width at half maximum (FWHM) of the anisochronous signal is measured, indicated by arrows and dotted lines on the left side. A graph is then plotted to show the relationship between the linewidth and the recorded internal temperature, shown on the right side. A Lorentzian curve is applied to this data, revealing the temperature at which the maximum linewidth is obtained. This temperature corresponds to T_c, with a value of 293.2±0.3 K, resulting from the fit of the experimental data.

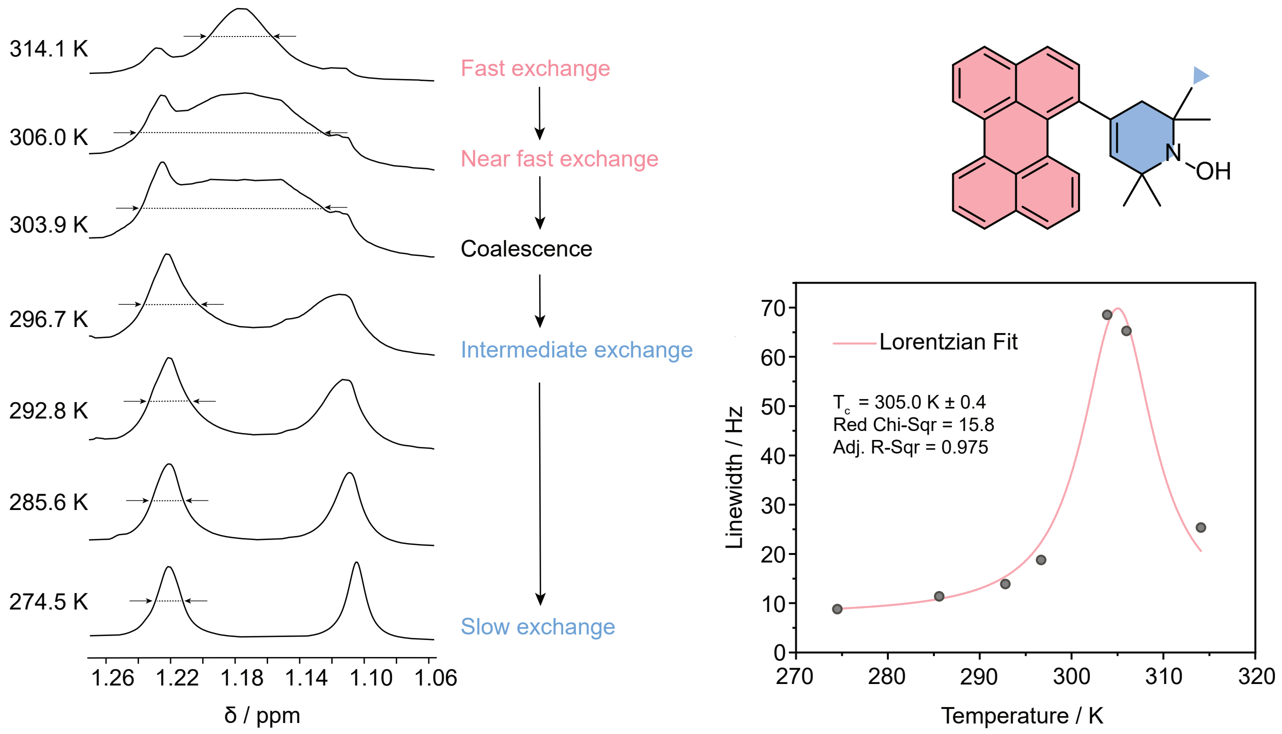

Figure S11: Process of determining the theoretical coalescence temperature, T_c, for per–1-*e*TEMPOH. The procedure is the same as explained above. For T_c a value of 305.0±0.4 K was obtained.

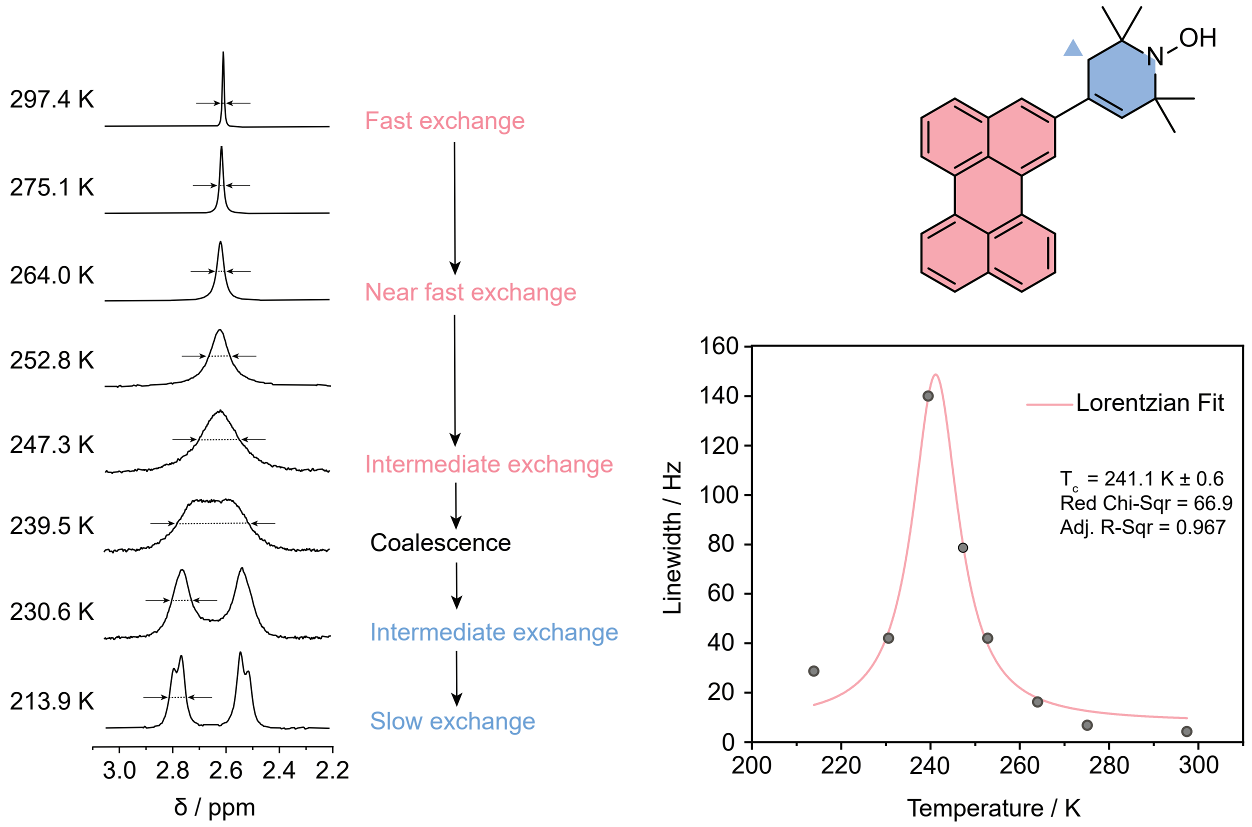

Figure S12: Process of determining the theoretical coalescence temperature, T_c, for per–2-*e*TEMPOH. The procedure is the same as explained above. For T_c a value of 241.1±0.6 K was obtained.

Enantiomerisation barriers

The barrier of enantiomerisation from the NMR results can be calculated according to the following equation: [9]

$$\Delta G^{\ddagger}_{\mathrm{Tc}} = R\,T_{\mathrm{c}}\left[\ln\left(\frac{k_{\mathrm{B}}\,T_{\mathrm{c}}}{h}\right) - \ln\left(\pi\frac{\Delta\nu}{\sqrt{2}}\right)\right]$$

where T_c is the theoretical coalescence temperature, k_{B} is the Boltzmann constant, h is the Planck constant, and $\Delta\nu$ the maximum peak separation in the low-temperature (i.e. slow-exchange) limit.

For **per–1-*e*TEMPOH** we obtained $\Delta G^{\ddagger}_{293.2} = 62.9\,\mathrm{kJ/mol}$ and $\Delta G^{\ddagger}_{305.0} = 62.4\,\mathrm{kJ/mol}$, while for **per–2-*e*TEMPOH** a barrier of $\Delta G^{\ddagger}_{241.1} = 47.5\,\mathrm{kJ/mol}$ was obtained.

Lineshape analysis

Dynamic Nuclear Magnetic Resonance (DNMR) spectroscopy is a well-established experimental technique for studying gradual nuclear exchange processes like hindered rotations and isomerisations, resulting in NMR signal broadening with temperature variations. As temperature increases, distinct signals broaden, merge, and then sharpen. DNMR utilises NMR parameters and hidden exchange rates to derive activation thermodynamic parameters. Spectrum fitting and parameter refining are achieved iteratively, commonly through simulation programs like DNMR5, MEXICO, WinDNMR, and Bruker's TOPSPIN DNMR module (utilised here). The DNMR Lineshape Fitting module is an interactive tool to simulate and refine temperature-dependent 1D NMR spectra, aiming at extracting exchange process kinetics at the measurement temperature.

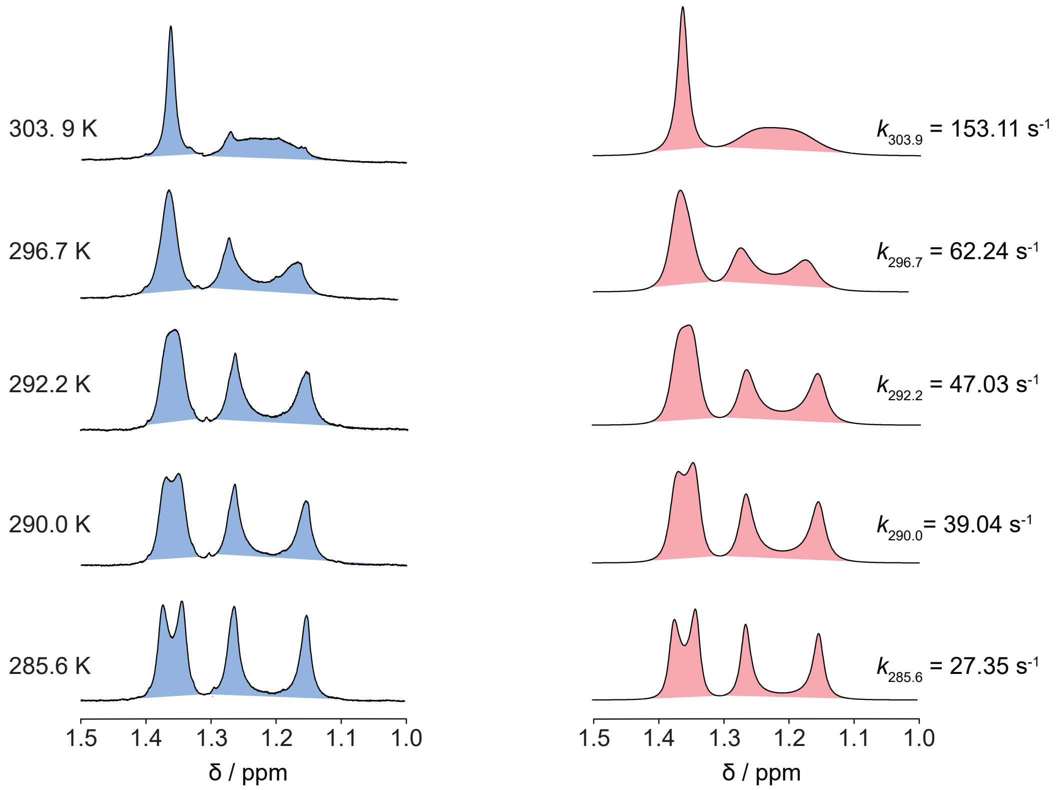

Figure S13: Lineshape analysis for per–1-*e*TEMPOH was employed to determine the values of $k_{\mathrm{e(T)}}$, the temperature-dependent rate constants. On the left side, the experimental spectra of the CH_3 groups of *e*TEMPO in THF-d_8 distinctly demonstrate the temperature-linked enantiomerisation, leading to observable coalescence phenomena. On the right side, the analysis of the spectral lineshapes yields calculated $k_{\mathrm{e(T)}}$ values.

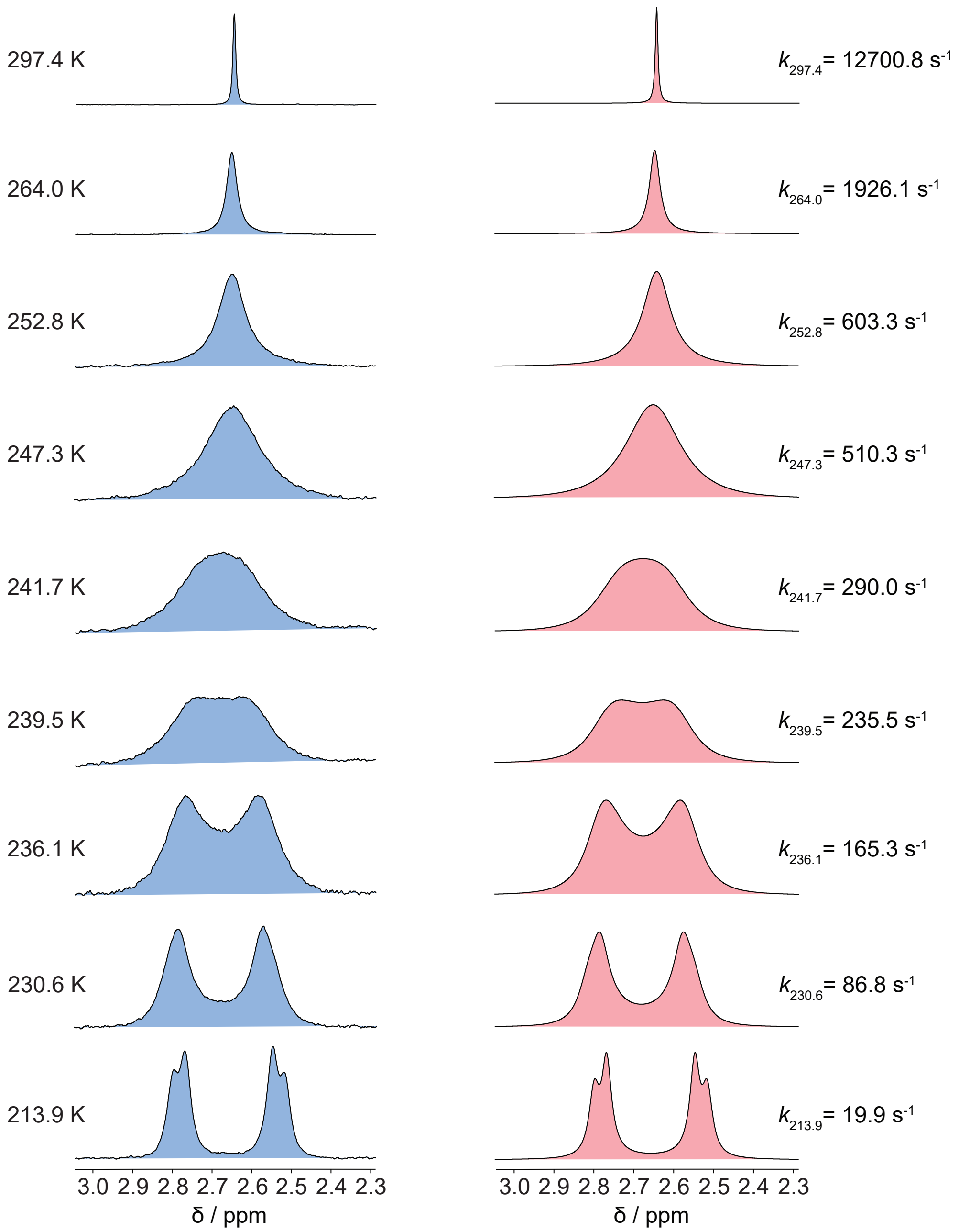

Figure S14: Lineshape analysis for per–2-*e*TEMPOH. Experimental spectra of the diastereotopic protons of the CH_2 group in THF-d_8 (*left*) and lineshape analysis (*right*).

Tables S1 and S2 below summarise the temperature-dependent rate constants obtained from the lineshape analysis.

Table S1: Experimental rate constants for per–1-*e*TEMPOH obtained from lineshape analysis.

$T_{\text{calibrated}}$ / K	Fitted $k_{e(T)}$ / s^{-1}
303.9	153.11
296.7	62.24
292.2	47.03
290.0	39.04
285.6	27.35

Table S2: Experimental rate constants for per–2-*e*TEMPOH obtained from lineshape analysis.

$T_{\text{calibrated}}$ / K	Fitted $k_{e(T)}$ / s^{-1}
297.4	12700.8
264.0	1928.1
252.8	603.3
247.3	510.3
241.7	290.0
239.5	235.5
236.1	165.3
230.6	86.8
213.9	19.9

Eyring plots

All experimental NMR spectra were fitted to obtain rate constants for the rotation process. These rate constants were then further analysed with Eyring plots ($R^2 \geq 0.97$) to get deeper insight into thermodynamic data.

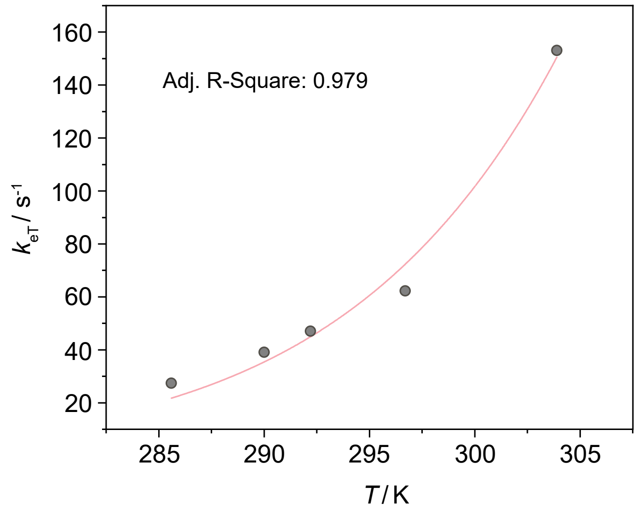

Figure S15: Eyring plot of the rates of per–1-*e*TEMPOH as determined by lineshape analysis. A nonlinear regression yields $\Delta H^{\ddagger}$ = 73.8 kJ/mol and $\Delta S^{\ddagger}$ = 39.5 J/mol.

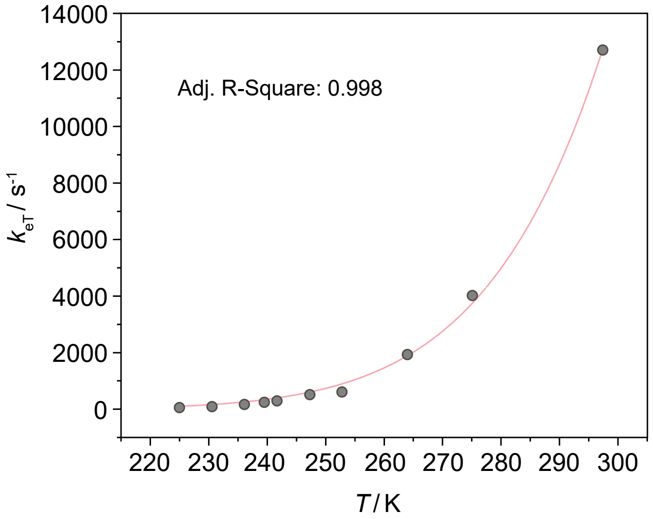

Figure S16: Eyring plot of the rates of per–2-*e*TEMPOH as determined by lineshape analysis. A nonlinear regression yields $\Delta H^{\ddagger}$ = 34.9 kJ/mol and $\Delta S^{\ddagger}$ = −48.8 J/mol.

Thermodynamic parameters

Inserting the thermodynamic parameters $\Delta H^{\ddagger}$ and $\Delta S^{\ddagger}$ determined from the nonlinear fits of the Eyring plots into the equation below allows the determination of the isomerisation barrier $\Delta G^{\ddagger}_{e}$.

$$\Delta G^{\ddagger}_{e} = \Delta H^{\ddagger}_{e} - T\Delta S^{\ddagger}_{e}$$

For **per–1-*e*TEMPOH** we obtain the following: $\Delta G^{\ddagger}_{293.2}$ = 62.2 kJ/mol, $\Delta G^{\ddagger}_{305.0}$ = 61.8 kJ/mol.

For **per–2-*e*TEMPOH** we obtain: $\Delta G^{\ddagger}_{241.1}$ = 46.7 kJ/mol.

Both analytical methods, lineshape analysis and T_c calculations, give comparable values for $\Delta G^{\ddagger}$. If we define $\Delta\Delta G^{\ddagger}$ as the difference of the isomerisation barrier between the $\Delta G^{\ddagger}$ obtained from the T_c calculations and the $\Delta G^{\ddagger}$ obtained from lineshape analysis, we obtained the following:

For **per–1-*e*TEMPOH**: $\Delta\Delta G^{\ddagger}_{293.2}$ = 0.7 kJ/mol, and $\Delta\Delta G^{\ddagger}_{305.0}$ = 0.6 kJ/mol.

For **per–2-*e*TEMPOH**: $\Delta\Delta G^{\ddagger}_{241.1}$ = 0.8 kJ/mol.

2 Steady state and time-resolved UV-vis spectroscopy

2.1 UV-vis spectroscopy

Steady-state absorption measurements of the samples in toluene were carried out on a V-770-ST UV-vis-NIR spectrometer from JASCO.

For the fluorescence measurements, the samples were diluted substantially, corresponding to absorbances < 0.1 at the excitation wavelength. Steady-state fluorescence spectra were recorded on a FluoroMax-4 fluorimeter from Horiba. The raw spectra were corrected for the spectral sensitivity of the instrument and fluctuations of the excitation light source.

Figure S17 shows the normalised UV-vis and fluorescence spectra of perylene. From the intersection of the two spectra, the $S_{0,0}$ energy can be determined. It amounts to 2.815 eV (= 440.5 nm).

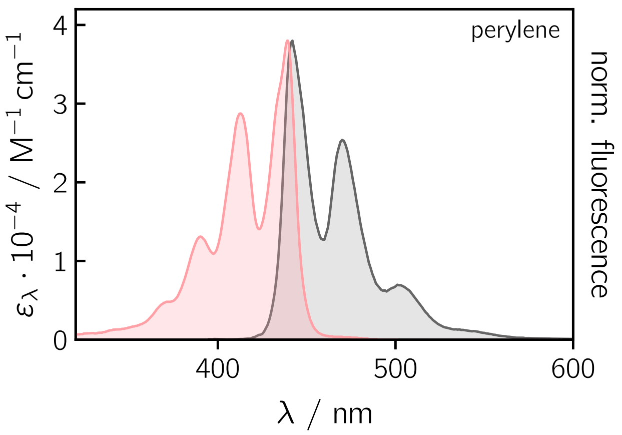

Figure S17: UV-vis absorption and fluorescence spectra of perylene recorded in toluene solution at room temperature. The UV-vis spectrum was scaled to match the reported molar absorption coefficient of $3.8 \cdot 10^4$ $M^{-1}cm^{-1}$.[10]

The normalised UV-vis absorption and fluorescence spectra of the three perylene–*e*TEMPO dyads are shown in Figure S18. It can be seen that, relative to perylene, the absorption and fluorescence spectra of all dyads are slightly red-shifted, with the largest shifts observed for per–3-*e*TEMPO. An overview of the photophysical properties is provided in Table S3.

Table S3: Overview of the photophysical properties of the dyads in toluene at 295 K. λ_{abs}: absorption maximum; λ_{flu}: fluorescence maximum; $S_{0,0}$: excited singlet state energy.

Compound	λ_{abs} / nm	λ_{flu} / nm	Stokes shift / cm^{-1}	$S_{0,0}$ / eV
per–1-*e*TEMPO	441.5	443.5	100	2.80
per–2-*e*TEMPO	441.5	447.5	300	2.79
per–3-*e*TEMPO	447	458	540	2.74

Fluorescence quantum yields were determined using a C11347 absolute photoluminescence quantum yield spectrometer from Hamamatsu Photonics K.K., Japan. The same solutions as prepared for the fluorescence measurements were used. An excitation wavelength of 400 nm was chosen and the analysis was performed using the tools provided with the data acquisition software.

Fluorescence lifetime measurements were carried out using a FluoTime 300 fluorescence lifetime spectrometer from Picoquant GmbH, Germany. The samples were excited at 355 nm and the scattering light

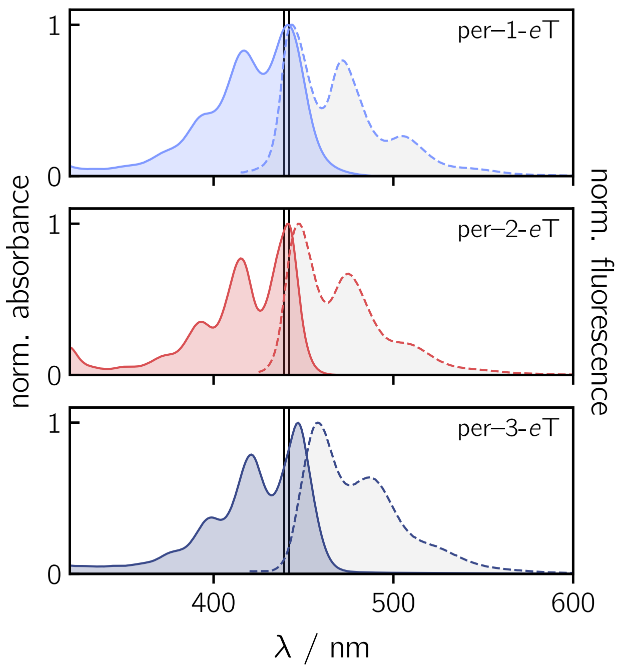

Figure S18: Normalised UV-vis absorption (solid lines) and fluorescence (dashed lines) spectra of the three perylene–*e*TEMPO dyads recorded in toluene solution at room temperature. The black vertical lines indicate the positions of the absorption and fluorescence intensity maxima of perylene.

from the excitation source was cut off with the help of a long-pass filter placed in the detection path. The instrument response function was collected (without any filters) using a solution of LUDOX® (colloidal silica) in distilled water. To obtain the fluorescence decay times, iterative re-convolution of the instrument response function with a monoexponential decay function was performed in MATLAB. The model decay function was fit to the experimental data using a least-squares fitting approach (minimisation of the residuals using a built-in trust-region-reflective algorithm). The data obtained for the bromoperylenes as well as the perylene reference are shown in Figures S19 and S20.

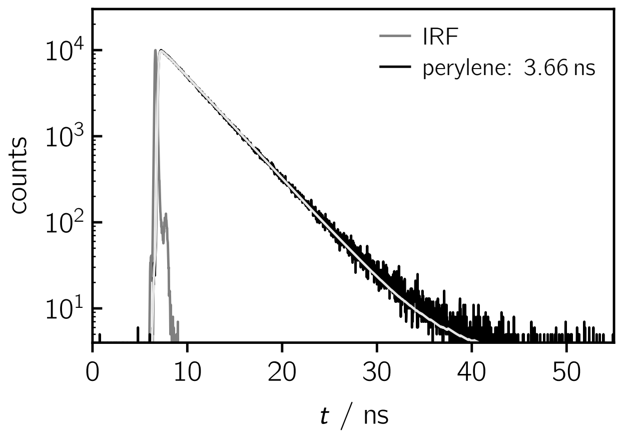

Figure S19: Fluorescence decay of perylene in toluene and best fit to the experimental data.

While the decay is monoexponential for 2-bromoperylene and 3-bromoperylene, with a fluorescence lifetime in the nanosecond range, the decay measured for 1-bromoperylene is clearly biexponential. The two time constants found for 1-bromoperylene have relative amplitudes of 11.7:1, indicating that the first time constant, in the picosecond range, is by far the main contribution. Unfortunately, this time constant, corresponding to the fluorescence lifetime of 1-bromoperylene, cannot be resolved reliably by single photon timing experiments given the time-resolution of the instrument. The second time constant

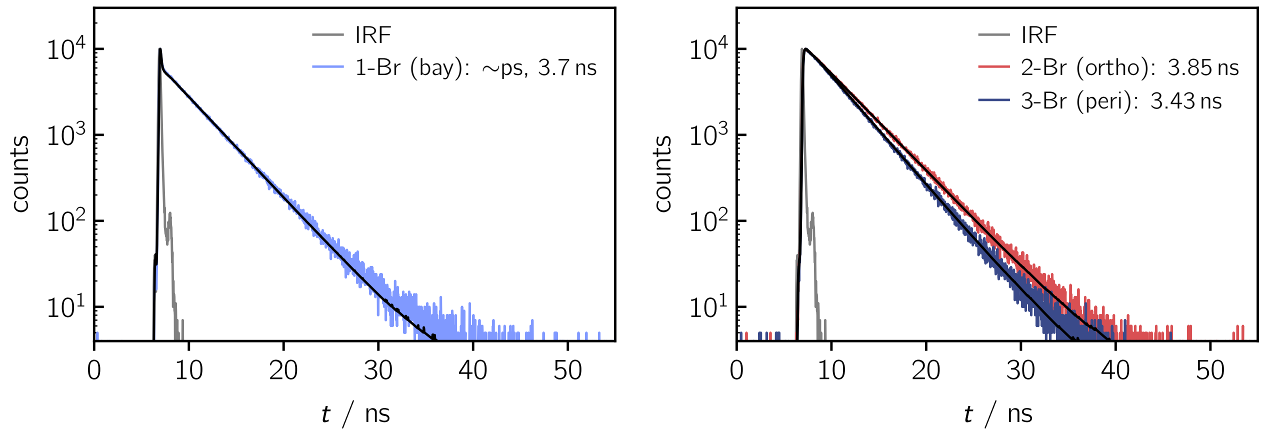

Figure S20: Fluorescence decays of the three bromoperylenes measured by single photon timing and best fit to the experimental data. While the fluorescence decays of 2-bromoperylene and 3-bromoperylene are monoexponential, the decay of 1-bromoperylene is clearly biexponential. Given the low fluorescence quantum yield of 1-bromoperylene and the ratio of the amplitudes obtained from the fit of the fluorescence decay trace, it is clear that the first time constant, in the picosecond range, corresponds to the true fluorescence lifetime of 1-bromoperylene. For further details please see the text.

likely indicates the presence of a small amount of residual perylene in the sample.

Assuming a direct correlation between the fluorescence quantum yields and lifetimes, a fluorescence lifetime of at most $\sim 270\,\mathrm{ps}$ can be estimated for 1-bromoperylene.

2.2 Calculation of the radiative rate constant using the Strickler-Berg equation

The radiative rate constant k_r^SB, as derived by Strickler and Berg[11] as well as Birks and Dyson[12], is given as

$$k_\mathrm{r}^\mathrm{SB} = \frac{8\pi\, c\, \ln(10)}{N_\mathrm{A}} \frac{n_\mathrm{f}^3}{n_\mathrm{i}} \frac{g_\mathrm{f}}{g_\mathrm{i}} \cdot \frac{\int F(\tilde{\nu})\,\mathrm{d}\tilde{\nu}}{\int F(\tilde{\nu})\,\tilde{\nu}^{-3}\,\mathrm{d}\tilde{\nu}} \cdot \int \varepsilon(\tilde{\nu})\,\tilde{\nu}^{-1}\,\mathrm{d}\tilde{\nu} \tag{S1}$$

where c is the speed of light, N_A the Avogadro constant, ε the molar absorption coefficient and $F(\tilde{\nu})$ the corrected fluorescence signal as a function of wavenumber. The parameter g represents the vibronic degeneracy of the final and initial states. Since the spectral shift between the absorption and emission spectra is small, the dispersion of the refractive index n can be assumed to be negligible and $\frac{n_\mathrm{f}^3}{n_\mathrm{i}}$ can be replaced by n^2. Assuming further that $g_\mathrm{f} = g_\mathrm{i}$, and using a molar absorption coefficient of $3.8 \cdot 10^4\,\mathrm{M^{-1}cm^{-1}}$,[10] the radiative rate constant calculated for perylene amounts to $k_\mathrm{r} = 2.38 \cdot 10^8\,\mathrm{s^{-1}}$.

Considering the measured fluorescence quantum yield of perylene (0.87) and the definition of the fluorescence quantum yield

$$\Phi_\mathrm{F} = \frac{N_\mathrm{emitted}}{N_\mathrm{absorbed}} = \frac{k_\mathrm{r}}{k_\mathrm{r} + k_\mathrm{IC} + k_\mathrm{ISC} + \ldots} = k_\mathrm{r} \cdot \tau_\mathrm{F} \tag{S2}$$

we obtain a calculated fluorescence lifetime of $\tau_\mathrm{F}^\mathrm{SB} = 3.65\,\mathrm{ns}$ for perylene, in excellent agreement with the measured value of 3.66 ns.

2.3 Calculation of the Förster resonance energy transfer rate constants

To judge the likelihood of Förster-type excitation energy transfer (FRET) taking place between perylene and *e*TEMPO, calculations of the theoretically expected EET rate constants were carried out for all three investigated dyads.

The Förster radius R_0 (obtained in nm) can be calculated from[13]

$$(R_0)^6 = 8.785 \cdot 10^{-11} \frac{\Phi^{\mathrm{D}}_{\mathrm{F},0}\,\kappa^2}{n^4} \int I^{\mathrm{D}}_{\mathrm{F}}(\lambda)\,\varepsilon^{\mathrm{A}}(\lambda)\,\lambda^4\,\mathrm{d}\lambda \tag{S3}$$

with

$$\int I^{\mathrm{D}}_{\mathrm{F}}(\lambda)\,\mathrm{d}\lambda = 1 \tag{S4}$$

where $\Phi^{\mathrm{D}}_{\mathrm{F},0}$ and $I^{\mathrm{D}}_{\mathrm{F}}$ are the fluorescence quantum yield and fluorescence intensity of the donor, ε^{A} is the molar absorption coefficient (in $\mathrm{M}^{-1}\mathrm{cm}^{-1}$) of the acceptor and n the refractive index of the medium. The orientation factor κ^2 accounts for the relative orientation of the two transition dipole moment vectors (emission of donor and absorption of acceptor) with respect to the axis connecting the FRET pair.

The energy transfer rate constant is then given as

$$\tau^{-1}_{\mathrm{FRET}} = k_{\mathrm{FRET}} = \frac{1}{\tau^{\mathrm{D}}_{\mathrm{F},0}} \left(\frac{R_0}{r_{\mathrm{DA}}}\right)^6 \tag{S5}$$

where $\tau^{\mathrm{D}}_{\mathrm{F},0}$ is the fluorescence lifetime of the donor in the absence of any quenchers and r_{DA} is the center-to-center distance (point dipole) between donor and acceptor.

As can be seen from the formulae for the calculation of the FRET rate constants, i.e. Equations (S3) to (S5), the efficiency of this process will depend primarily on (i) the spectral overlap of chromophore emission and radical absorption and, (ii) the relative orientation of the transition dipole moments of chromophore and radical (κ^2), in addition to the distance r_{DA} between the donor and acceptor moieties. A visualisation of the spectral overlap is shown in Figure S21.

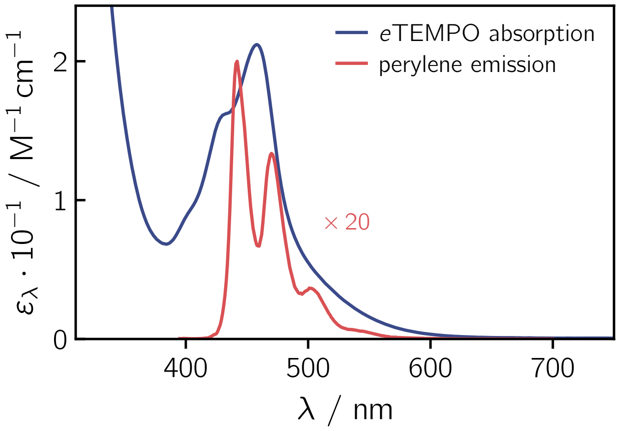

Figure S21: UV-vis absorption spectrum of the *e*TEMPO radical together with the fluorescence emission spectrum of perylene for an illustration of the spectral overlap.

The transition dipole moment (TDM) of the perylene chromophore lies along the long axis of the molecule, while that of the *e*TEMPO radical is perpendicular to the chromophore–radical bonding axis (i.e. perpendicular to the N–O axis).[14] Based on the knowledge of the orientation of the TDMs of chromophore and radical, we calculated the κ^2 values for the three dyads. We assumed a relative orientation of the two moieties as obtained from a DFT geometry optimisation (vide infra) of the dyad structures and considered all conformers. The Boltzmann-weighted (295 K) calculated κ^2 values obtained this way amount to 0.306, 0.154, and 0.086 for per–1-*e*TEMPO, per–2-*e*TEMPO, and per–3-*e*TEMPO, respectively.

For the calculation of the FRET rate constants k_{FRET} for the perylene–*e*TEMPO dyads, the molar absorption coefficient of the *e*TEMPO radical was taken to amount to 21.2 $M^{-1}cm^{-1}$ at its absorption maximum of 458 nm, as determined previously.[14] The fluorescence quantum yield and lifetime of the perylene chromophore were measured as detailed above and amount to $\Phi_F = 0.87$ and $\tau_F = 3.7$ ns, respectively, while the centre-to-centre distances, r_{DA}, were taken from DFT models of the structures (vide infra) and amount to 0.73 nm for per–1-*e*TEMPO, 0.87 nm for per–2-*e*TEMPO, and 0.87 nm for per–3-*e*TEMPO. An overview of the results and parameters is provided in Table S4.

Table S4: Overview of the results from the calculation of the Förster radius and FRET time constant for the investigated structures. The following parameters were used: $r_{\mathrm{DA}} = 0.73$ nm for per–1-*e*TEMPO and 0.87 nm for per–2-*e*TEMPO and per–3-*e*TEMPO, $n = 1.496$ (toluene), $\tau^{\mathrm{D}}_{\mathrm{F},0} = 3.66$ ns, $\Phi^{\mathrm{D}}_{\mathrm{F},0} = 0.87$, $\varepsilon^{\mathrm{A}} = 21.2\,M^{-1}cm^{-1}$ for *e*TEMPO.

compound	κ^2	R_0 / nm	τ_{FRET} / ns
per–1-*e*TEMPO	0.306	1.19	0.19
per–2-*e*TEMPO	0.154	1.07	1.06
per–3-*e*TEMPO	0.086	0.96	2.00

2.4 Femtosecond TA data acquisition

The setup has been described in detail elsewhere.[15–18] In brief, a Ti:Sapphire laser amplifier system (Coherent Libra) with a repetition rate of 1 kHz, a pulse duration of 100 fs, and a wavelength of 800 nm was used as the pulse source. Part of its output was frequency doubled (type I, 1 mm BBO, 29.2°) to obtain an excitation wavelength of 400 nm. The pump power at the sample position amounted to 1 mW (i.e., 1 μJ/pulse). For probing (330−740 nm), a supercontinuum was generated in a CaF_2 plate. The pump beam diameter at the sample was ∼160 μm (FWHM), while the diameter of the probe beam was ∼100 μm. The relative polarisation of pump and probe beams was set to the magic angle and the instrument response time was ∼180 fs (FWHM).

Femtosecond transient UV-vis absorption (fsTA) spectra were recorded at 139 different time delays, where 50 linear time steps between −1 and 1 ps were followed by linear steps on a logarithmic scale up to roughly 4 ns. A total of 2500 spectra were acquired per time point and averaged over two successive scans. For every time point, four different sets of data were collected: (i) only the white light (probe) reaches the sample, (ii) pump and probe both blocked, (iii) pump and probe both reach the sample, (iv) only the pump reaches the sample. Signals (i) and (iii) are used for the calculation of ΔA, while signals (ii) and (iv) are needed to account for dark signals and pump light scattering. The chirp of the white light was measured in a separate (optical Kerr effect, OKE) experiment and accounted for in the processing of the TA data. In addition, solvent spectra were recorded separately under identical conditions as the samples and subtracted from the sample data following the procedure detailed in reference 19.

The TA data were analysed using lab-written MATLAB routines. After subtraction of the suitably scaled solvent background, the data were chirp-corrected by interpolation in the time domain using a function of the form $f(t_D) = p_1 + \frac{p_2}{t_D^2} + \frac{p_3}{t_D^4}$, where $f(t_D)$ is the instrumental delay time and the parameters p_n are obtained by fitting the temporal peaks of the OKE response as a function of wavelength.

For the room temperature femtosecond TA experiments the samples were prepared in toluene solutions with an absorbance between 0.2 and 0.3 at the excitation wavelength of 400 nm in a 1 mm cuvette. During

the experiments, the sample solutions (∼ 2 mL) were flown continuously. In addition, UV-vis spectra were taken before and after the measurements to verify the sample absorbances and confirm the absence of sample degradation during the measurement.

2.5 Determination of the triplet yields

As explained in detail in the Supporting Information of reference 20, the triplet yield of the dyads can be estimated by a careful analysis of the time-dependence of the ground state bleach amplitude in fsTA experiments.

The triplet yield of the three dyads was determined from the corresponding fsTA data by comparing the amplitude of the perylene ground state bleach directly after photoexcitation and at a later time, corresponding to $5 \times \tau_{S1}$, where perylene triplet state formation can be considered to be complete. Considering also the contribution of excited state absorption and stimulated emission to the absorption signal at the position of the ground state bleach, values of roughly 75 %, 70 %, and 40 % were obtained for per–1-*e*TEMPO, per–2-*e*TEMPO, and per–3-*e*TEMPO, respectively. We estimate the error on these values to be less than ±5 %. For per–1-*e*TEMPO, photodegradation of the sample was observed during the measurement, potentially leading to a larger uncertainty (also regarding the time constant of triplet formation).

2.6 Additional fsTA data and global kinetic analysis

For reference, a contour plot of the fsTA data recorded for perylene in toluene is shown in Figure S22.

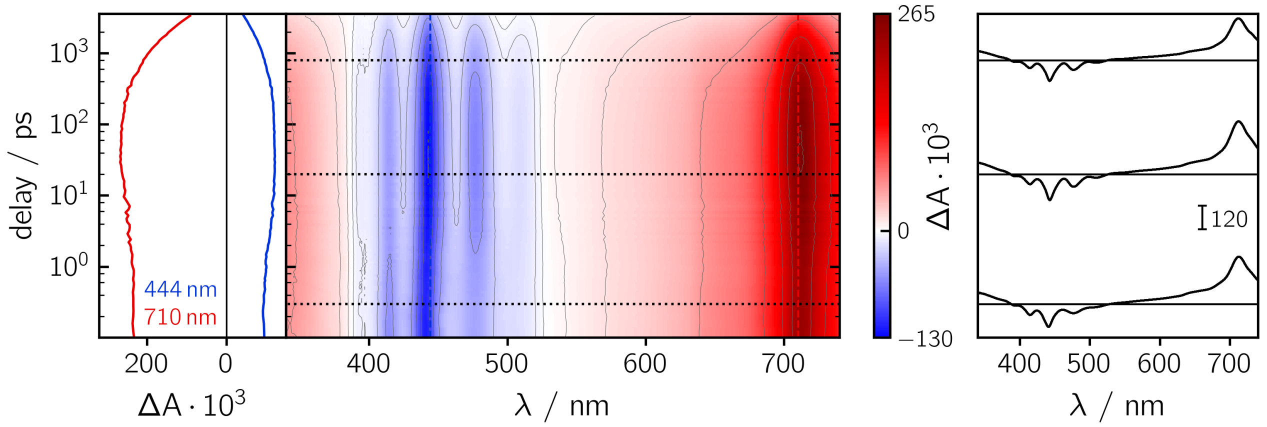

Figure S22: Contour plot of the fsTA data for perylene recorded in toluene solution at room temperature after photoexcitation at 400 nm.

To determine the time constants of the excited state deactivation processes, a global kinetic analysis of the fsTA data was carried out for all compounds. In a first step, the number of time constants required to reproduce the experimental data satisfactorily was determined by computation of the decay associated spectra (DAS).[21] Here, a multi-exponential function convoluted with the instrument response function (IRF) served as the trial function. A time constant and an offset were required for all three dyads. The time constant τ_1 corresponds to the decay of the excited singlet state of the perylene derivatives. The remaining signals (offset) represent the triplet/quartet state, the lifetime of which cannot be assessed by fsTA measurements. The DAS obtained for the three dyads are shown in Figure S23.

In a second step, species associated spectra (SAS) were computed assuming a branched model of the form:

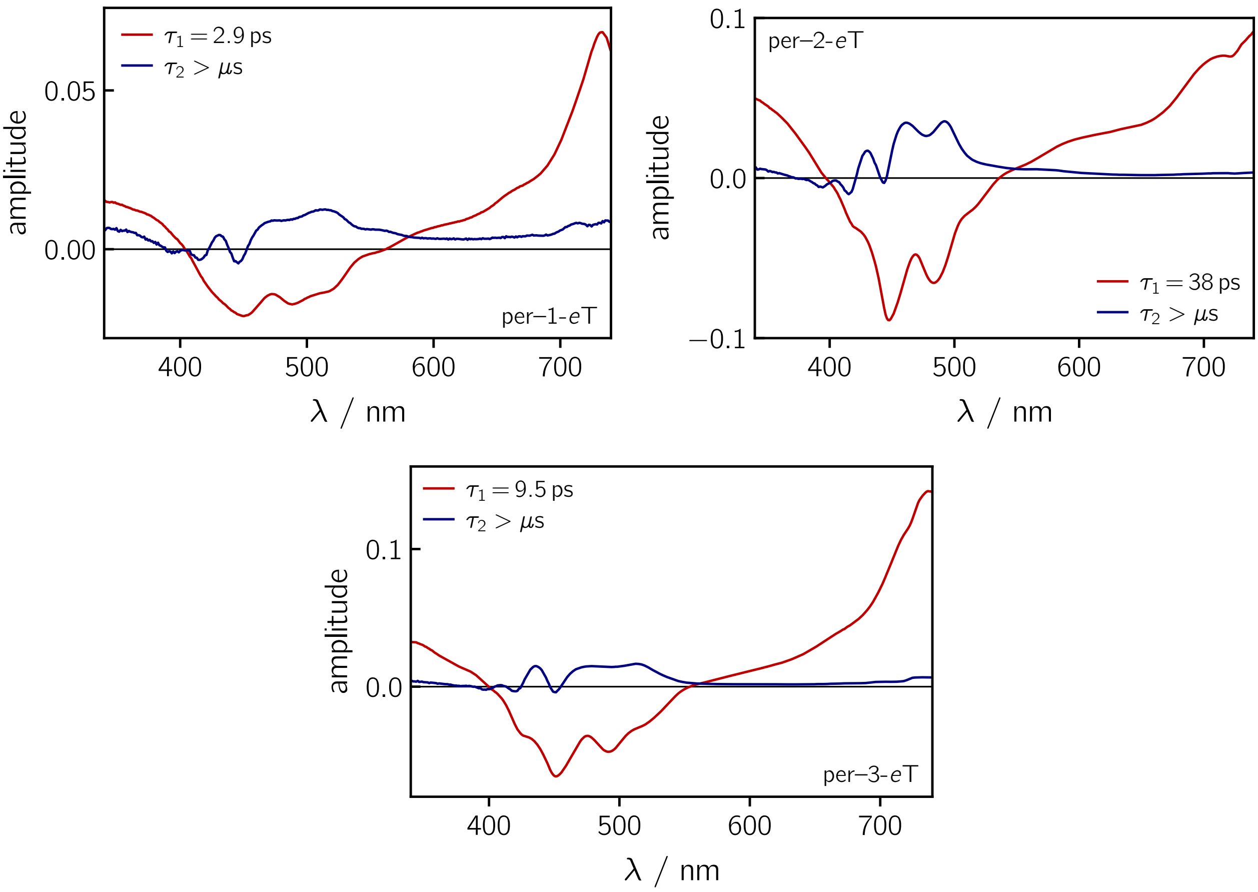

Figure S23: Decay associated spectra and time constants obtained from a global kinetic analysis of the fsTA data of per–1-*e*TEMPO (*left*, *top*), per–2-*e*TEMPO(*right*, *top*), and per–3-*e*TEMPO (*bottom*) in toluene.

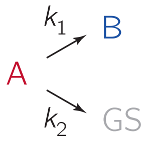

per-1-*e*TEMPO: $\Phi_T = 0.75 \rightarrow k_2 \sim 0.33\, k_1$

per-2-*e*TEMPO: $\Phi_T = 0.70 \rightarrow k_2 \sim 0.43\, k_1$

per-3-*e*TEMPO: $\Phi_T = 0.40 \rightarrow k_2 \sim 1.5\, k_1$

The species A and B are assigned to the excited singlet state of perylene and the perylene triplet state, respectively. Part of the excited state population (A) is assumed to decay directly to the ground state by enhanced internal conversion, explaining the experimentally determined triplet yields below unity. The branching ratio was adapted based on the experimentally determined triplet yields. The SAS, obtained by plotting the contributions (i.e. amplitudes) of the individual time constants as a function of wavelength and assuming the validity of the chosen branched model, are shown in Figures S24 to S26.

The time constants obtained for the formation and decay of the different species (elementary time constants) assuming a branched kinetic model are slightly different as compared to the lifetimes from the model-free analysis described above. In addition, when incorporating the known triplet yield as an additional constraint, the time constant of triplet state formation can be calculated. Table S5 gives a summary of these time constants.

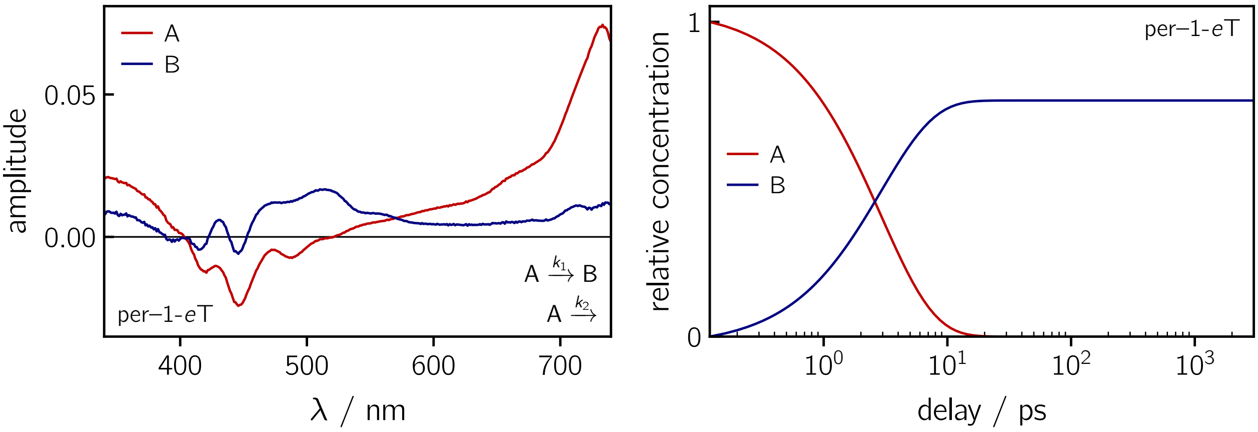

Figure S24: Species associated spectra obtained from a global kinetic analysis of the fsTA data of per–1-*e*TEMPO in toluene (*left*) and corresponding kinetics (*right*).

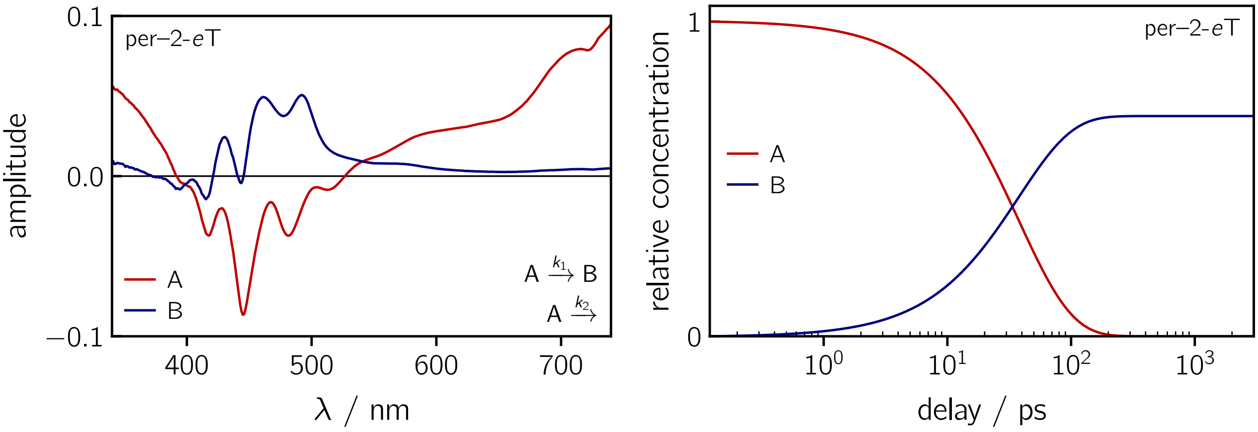

Figure S25: Species associated spectra obtained from a global kinetic analysis of the fsTA data of per–2-*e*TEMPO in toluene (*left*) and corresponding kinetics (*right*).

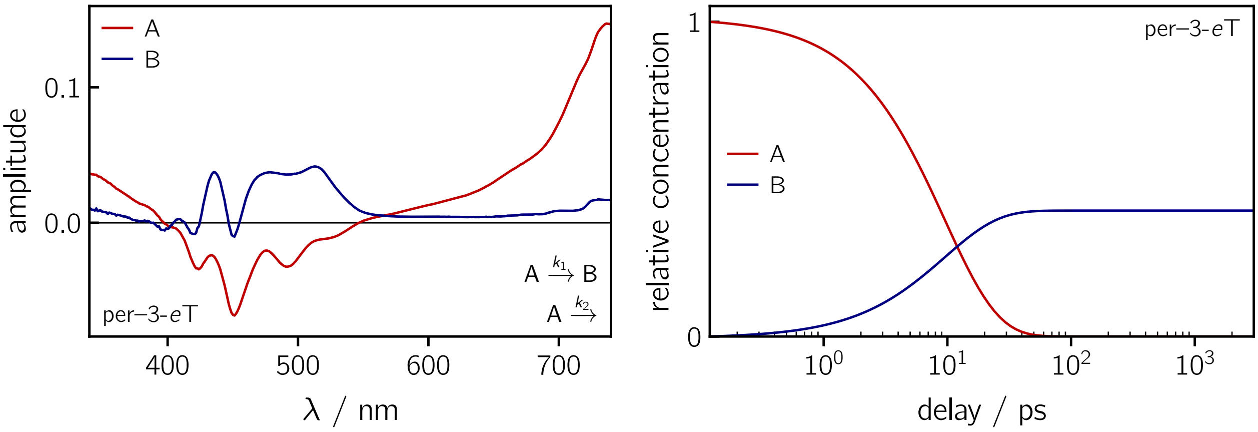

Figure S26: Species associated spectra obtained from a global kinetic analysis of the fsTA data of per–2-*e*TEMPO in toluene (*left*) and corresponding kinetics (*right*).

Table S5: Time constants obtained from a global kinetic analysis of the fsTA data of per–1-*e*TEMPO, per–2-*e*TEMPO, and per–3-*e*TEMPO recorded in toluene solution at room temperature. A blue colour coding is used for the time constant corresponding to deactivation of the chromophore S_1 state.

compound	τ_1^{DAS} / ps	τ_2^{DAS} / ps	τ_1^{SAS} / ps
per–1-*e*TEMPO	2.9	$> 10^6$	3.9
per–2-*e*TEMPO	38	$> 10^6$	54
per–3-*e*TEMPO	9.5	$> 10^6$	24

3 EPR characterisation

3.1 EPR sample preparation

EPR samples in toluene solution. For the transient EPR measurements in isotropic frozen solution, the samples were prepared with an absorbance of roughly 0.3 at the excitation wavelength of 435 nm, measured in a 2 mm cuvette. The sample solutions were then transferred into quartz EPR tubes with an outer diameter of 3.8 mm or 1.6 mm (inner diameter of 3 mm or 1 mm) for measurements at the X-band or Q-band, respectively. The solutions were rapidly frozen in liquid nitrogen before insertion into the EPR resonator for the measurement.

3.2 EPR setup and parameters

Continuous wave EPR spectroscopy. The spectra were recorded at the X-band (9.75 GHz) at room temperature on a Bruker EMXnano benchtop EPR spectrometer using quartz EPR tubes with an outer diameter of 3.8 mm (inner diameter of 3 mm). The modulation frequency was set to 100 kHz and the modulation amplitude to 0.1 mT at a microwave power of 0.25 mW (26 dB). The recorded, background-corrected, spectra were frequency-corrected to 9.75 GHz and field-corrected using a carbon fibre standard with $g = 2.002644$.[22]

Transient continuous wave EPR spectroscopy. Transient continuous wave EPR measurements were performed at the X-band (9.75 GHz) on a Bruker ELEXSYS E580 spectrometer at a constant temperature of 80 K, using an Oxford Instruments nitrogen gas-flow cryostat (CF 935). The samples were excited directly with depolarised laser light through the optical window of the cryostat and resonator (without an optical fibre). Excitation energies of ~2.3 mJ were used.

The spectra were acquired in direct detection mode with a video amplifier bandwidth of 20 MHz using the built-in transient recorder and a microwave power of 1.5 mW (20 dB). Any positive signals thus correspond to an absorptive transition (*a*) and any negative signals to an emissive (*e*) one. The transient recorder was used in AC-AFC mode and the transient signal was fed through a low-noise voltage preamplifier (Stanford Research Systems SR 560) with a bandpass filter of 3 kHz−1 MHz before entering the detection circuitry. Typically, for every magnetic field value, a time trace with 4096 points was recorded using a time base of 4 ns. After data acquisition, the 2D spectra were baseline-corrected in both dimensions using a lab-written MATLAB routine.

The spectra shown in the figures are time-slices at 0.8 μs after laser excitation unless otherwise stated. All X-band trEPR spectra were frequency-corrected to 9.75 GHz and field-corrected using a carbon fibre standard with $g = 2.002644$.[22]

Transient pulse EPR spectroscopy. Transient pulse EPR measurements were performed at Q-band frequencies (34.0 GHz) on a Bruker ELEXSYS E580 spectrometer equipped with a Bruker EN45107D2 resonator. During the measurement, the sample was kept at a constant temperature of 80 K using an Oxford Instruments nitrogen gas-flow cryostat (CF 935). The samples were excited through the top of the sample holder with depolarised light at 435 nm using an optical fibre with a diameter of 0.8 mm. The excitation energy was ~0.5 mJ at a repetition rate of 50 Hz (pulse duration ~5 ns).

Echo-detected field-swept EPR spectra at the Q-band were recorded using the sequence $h\nu - \mathrm{DAF} - \pi/2 - \tau - \pi - \tau -$ echo with DAF $= 9\,\mu$s at 80 K (DAF $\equiv$ delay after flash), $\tau = 180$ ns, and a π-pulse length of 32 ns. Unless otherwise stated, a two-step phase cycle was applied in all pulse EPR experiments.

The same pulse lengths were also used for the determination of T_m. To record T_m of the quartet state, a pre-saturation pulse was applied to avoid any contributions of the radical signal to the spin relaxation decay of the quartet state.

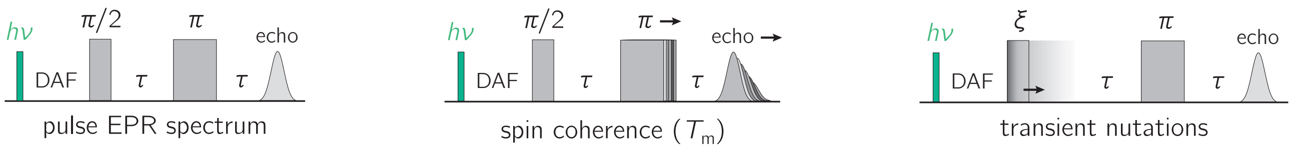

Transient nutation measurements used the sequence $h\nu$ – DAF – ξ – τ – π – τ – echo where the flip angle ξ was gradually increased by increasing the corresponding microwave pulse length in steps of 2 ns, starting at 16 ns ($\pi/2$). At specific magnetic field positions within the region of the quartet spectrum, the integrated echo intensity was then recorded as a function of this pulse length. The data were background corrected using a polynomial background function. The (cross-term averaged) Fourier transform was then calculated after dead-time reconstruction, windowing using a Hamming window, and zero filling to 2048 data points. The frequency spectra were normalised by division of the frequency axis by the reference frequency obtained for the same sample in the dark (doublet multiplicity). Whenever the dark state contribution to the transient signal at the chosen field position was still significant, a pre-saturation pulse was applied to limit any such contributions.

Saturation recovery measurements. T_1 of the dark state (*e*TEMPO radical) in frozen toluene at 80 K was measured by echo-detected saturation recovery making use of the Picket-fence method. The saturation pulse was composed of a train of 28 π pulses with a length of 32 ns each and an inter-pulse delay t of 128 ns between the individual pulses. The magnetisation was detected after a delay T with a Hahn echo sequence with pulse lengths of 16 ns and 32 ns for π and $\pi/2$ pulses, respectively and an inter-pulse delay τ of 180 ns. The delay T between the last π pulse of the Picket train and the echo detection sequence was incremented in non-linear time steps such that $T_{n+1} = T_n + n\,\Delta t_{\text{step}}$. A relaxation curve with $n = 128$ points was then recorded with a time step Δt_{step} of 496 ns, corresponding to a maximum T value of 4 ms. Unwanted echos were averaged out making use of a four-step phase cycle.

3.3 EPR characterisation of the radical and triplet precursors

The room temperature continuous wave (cw) EPR spectra of all three dyads, dissolved in toluene, are overlaid in Figure S27.

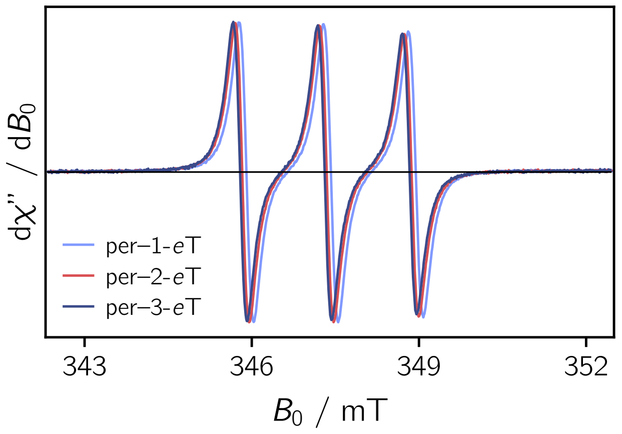

Figure S27: Comparison of the room temperature cw EPR data (field-modulated ≃ derivative shape) of all three investigated dyads.

The dark state cw EPR spectra of the dyads exhibit the characteristics of a typical nitroxide spectrum. Three lines with a peak separation of ∼1.5 mT are observed, resulting from the coupling of the unpaired electron spin to the ^{14}N nucleus ($I = 1$) of the nitroxide group.

To determine the nitroxide **g** and **A** tensors, as needed for the simulation of the trEPR spectra of the coupled dyads, cw EPR and pulse Q-band EPR data of *e*TEMPO were recorded and later fitted simultaneously. The data and fits are shown in Figure S28.

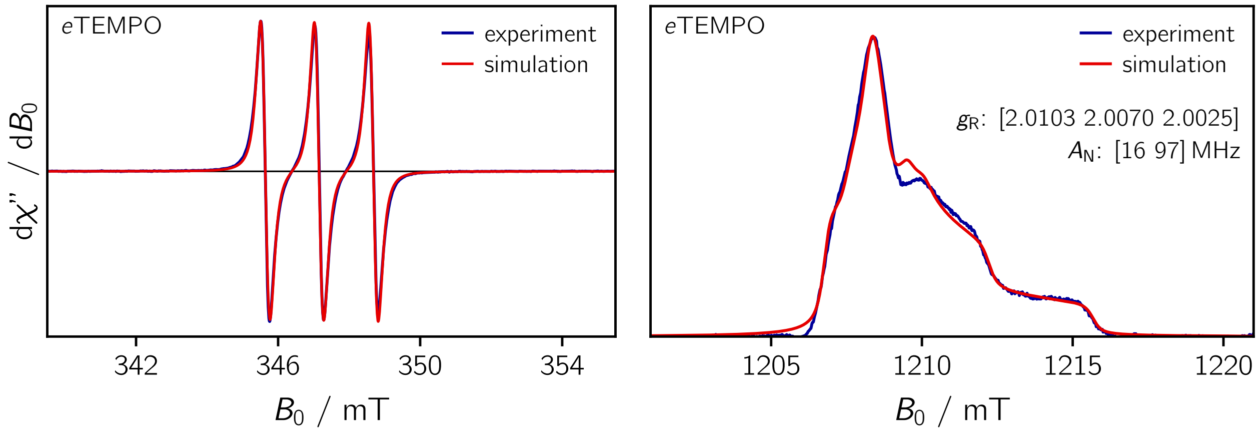

Figure S28: Determination of the **g** and **A** tensors of the *e*TEMPO radical by simultaneous fitting of its X-band room temperature cw EPR spectrum (*left*) and corresponding pulse Q-band spectrum recorded in frozen toluene solution at 80 K (*right*).

The triplet parameters for the simulation of the trEPR spectra of the coupled dyads were taken from simulations of the trEPR spectra of the respective bromoperylene precursors. The data were recorded at the X-band in frozen toluene solution at 80 K and are shown in Figure S29 together with the best fits to the data. An overview of all simulation parameters is provided in Table S6.

Table S6: Overview of the simulation parameters used to reproduce the triplet spectra of the three bromoperylenes.

compound	g_{iso}	D_T / MHz	E_T / MHz	HStrain / MHz	[P_X P_Y P_Z]
1-Br	2.0058	1426	−54	[295 95 380]	[0.58 0.42 0]
2-Br	2.0058	1627	−72	[330 130 340]	[0.48 0.52 0]
3-Br	2.0058	1560	−32	[320 170 210]	[1.00 0 0]

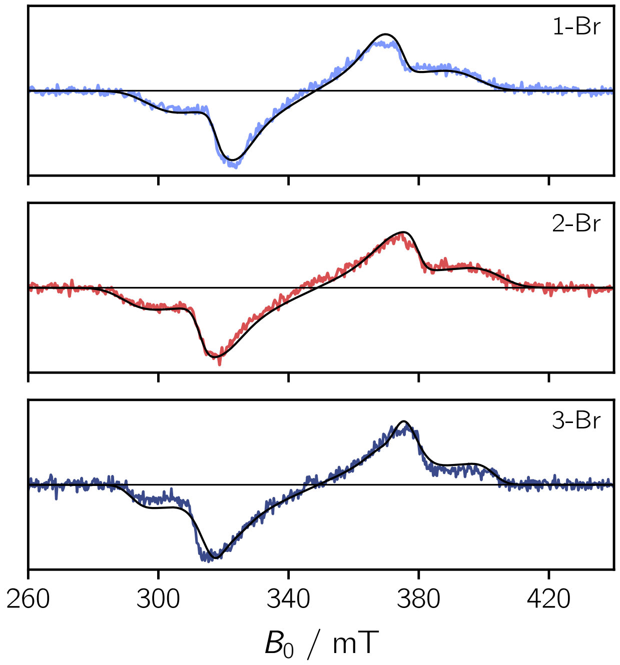

Figure S29: Comparison of the trEPR data of the bromoperylene precursors, recorded in frozen toluene solution at 80 K. The best triplet state simulations using a spin Hamiltonian approach are overlaid (black solid lines). The simulation parameters are given in Table S6.

3.4 Simulation of the trEPR spectra

The frozen solution trEPR spectra of per–1-*e*TEMPO, per–2-*e*TEMPO, and per–3-*e*TEMPO, shown in the main part, were simulated using the current developer version of EasySpin (v6.0.0-dev.50).[23,24] The function 'pepper' was used for the simulation of the frozen solution spectra of the coupled spin systems and the simulation was carried out in the 'coupled' basis. During the fitting procedure, the magnetic parameters of the radical and chromophore precursor molecules ($\boldsymbol{g}_{\mathrm{R}}$, $\boldsymbol{g}_{\mathrm{T}}$, D_{T}) were kept fixed, so that only the coupling parameters (D_{TR}, J_{TR}), linewidths, and the populations of the quartet spin states needed to be adapted. The results are summarised in Table S7.

The exchange interaction J_{TR} was assumed to be ferromagnetic for all three compounds, as predicted by quantum chemical calculations (see Section 4.4 below). Ferromagnetic coupling corresponds to a negative sign of J in EasySpin. Note that the exchange coupling calculations shown further below and the EasySpin software rely on a different definition of the exchange coupling Hamiltonian: in our quantum chemical calculations (Section 4.4), a positive value of J_{TR} indicates ferromagnetic coupling (i.e. $E_{\mathrm{Q}} < E_{\mathrm{D1}}$).

Table S7: Simulation parameters for the coupled perylene–*e*TEMPO systems.

	per–1-*e*TEMPO	per–2-*e*TEMPO	per–3-*e*TEMPO
g_R	[2.0103 2.0070 2.0025]	[2.0103 2.0070 2.0025]	[2.0103 2.0070 2.0025]
g_T	2.0058	2.0058	2.0058
D_T / MHz	1426	1627	1560
E_T / MHz	−10	−94	−35
J_{TR} / MHz	−40000	−40000	−40000
D_{TR} / MHz	[−320 −320 640]	[−70 −70 140]	[−240 −240 480]
populations			
$Q_{+3/2}$	0.52	0.64	0.44
$Q_{+1/2}$	0.09	0.23	0.09
$Q_{-1/2}$	0	0.13	0
$Q_{-3/2}$	0.39	0	0.47
HStrain / MHz	[370 390 95]	[150 280 75]	[140 240 260]
DStrain / MHz	[730 2]	[640 2]	[200 1]

3.5 Additional pulse EPR data

Figure S30 shows the pulse EPR spectra recorded for the three perylene–*e*TEMPO dyads in the dark and after photoexcitation at 435 nm. The light-induced signal was found to be very weak in all cases, with the largest changes being observed for per–1-*e*TEMPO. The light-induced spectra were recorded after application of a pre-saturation pulse adapted to suppress the dark state signal. The field positions where the relaxation measurements were performed are indicated by vertical lines.

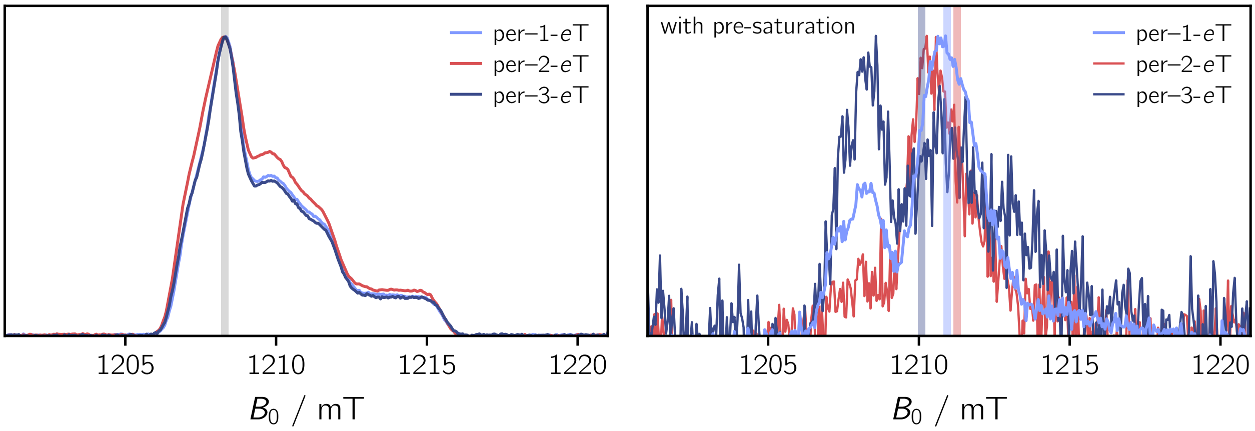

Figure S30: Comparison of the pulse EPR spectra of the perylene–*e*TEMPO dyads recorded at the Q-band at 80 K in the dark (*left*) and after photoexcitation at 435 nm (*right*). The light-induced signal, shown on the right, was recorded after application of a pre-saturation pulse adapted to suppress the dark state signal. The vertical lines indicate the field positions where the relaxation and transient nutation experiments were performed.

To confirm the quartet nature of the light-induced signal, transient nutation measurements were performed. Due to the extremely weak pulse EPR signals obtained for per–2-*e*TEMPO and per–3-*e*TEMPO, transient nutation data could only be recorded for per–1-*e*TEMPO and are shown in Figure S31.

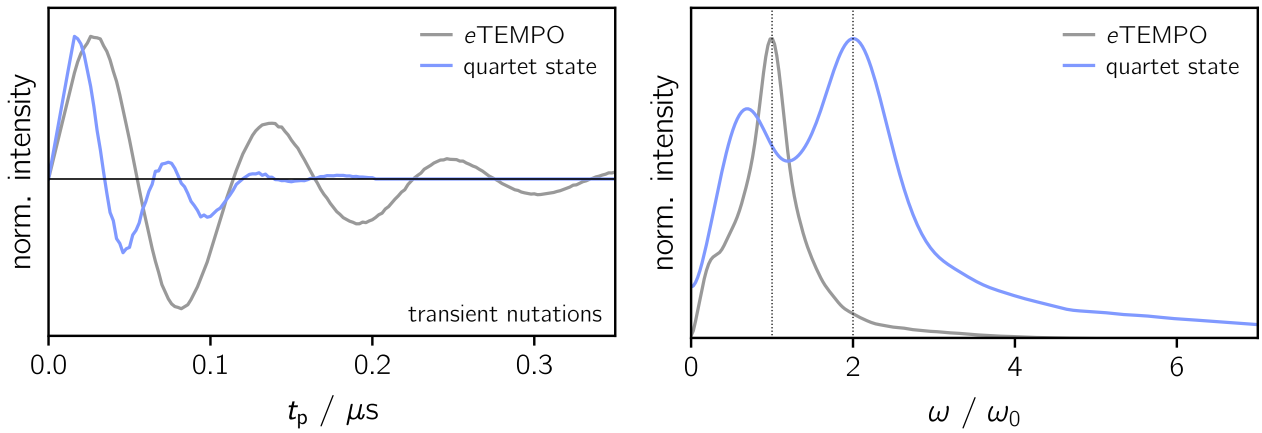

Figure S31: Transient nutation data recorded for per–1-*e*TEMPO in frozen toluene solution at 80 K (*left*) and corresponding Fourier transform (*right*). The field position where the measurement was performed is indicated in the light-induced spectrum shown in the figure above. To obtain the reference frequency ω_0 of the *e*TEMPO radical the same experiment was repeated in the dark.

The light-induced trace is affected by a small residual contribution of the dark state signal, although a pre-saturation pulse was applied to record the data. Nevertheless, the frequency of $2\,\omega_0$, expected for the $|-1/2\rangle \leftrightarrow |+1/2\rangle$ transition of the quartet state, could clearly be detected, leaving no doubt about the quartet nature of the signal.

Figures S32 to S34 show a comparison of the relaxation properties (T_m, T_1) of the three dyads. The measurements were performed in isotropic frozen toluene solution at 80 K. Due to the poor light-induced signal, T_1 could only be measured for the dark state (i.e., *e*TEMPO radical).

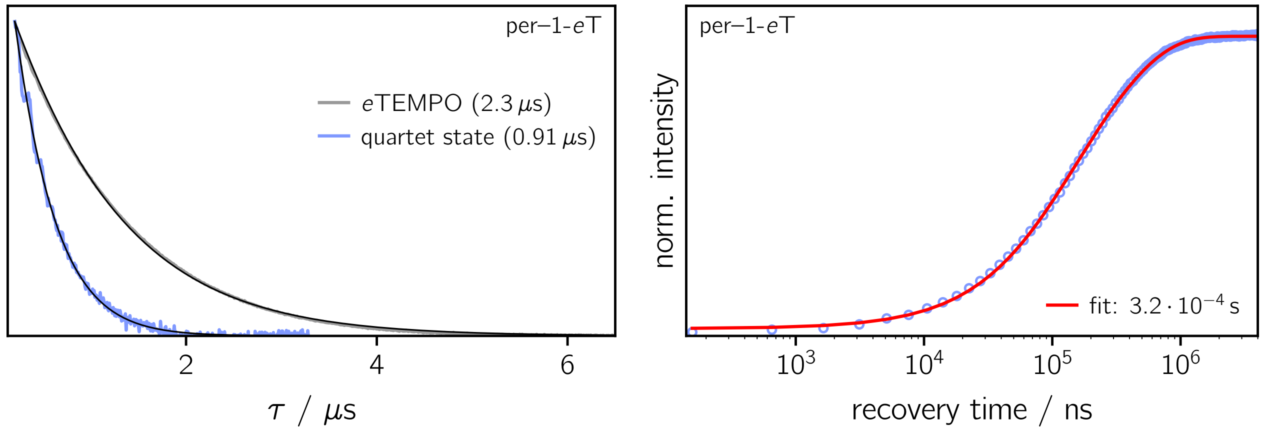

Figure S32: Spin echo decay (*left*) and saturation recovery (*right*) data recorded for per–1-*e*TEMPO in frozen toluene solution at 80 K together with the best fit to the data (solid black or red line). The obtained relaxation times are indicated.

The spin echo decays were fit assuming a stretched exponential model of the form $V_{SE} = \exp(-(2\tau/T_m)^{\beta})$, while a biexponential fit was applied to the saturation recovery data. The obtained spin relaxation times are indicated in the figures. In the case of the saturation recovery experiment, the longer of the two obtained τ values was assumed to correspond to T_1. The β values obtained when fitting the spin echo decay traces recorded in the dark (*e*TEMPO radical) and after photoexcitation at 435 nm (quartet state $|-1/2\rangle \leftrightarrow |+1/2\rangle$ transition) are summarised in Table S8 below.

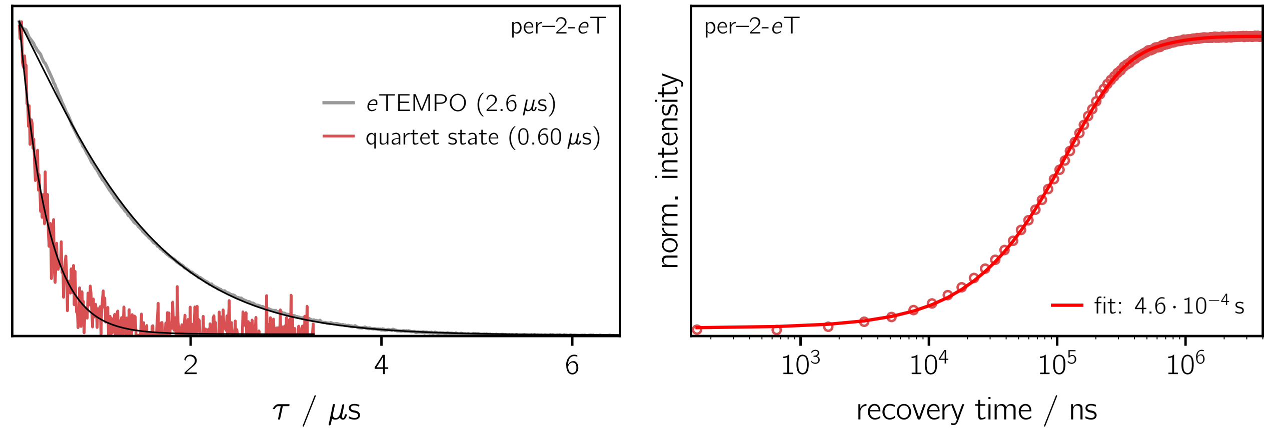

Figure S33: Spin echo decay (*left*) and saturation recovery (*right*) data recorded for per–2-*e*TEMPO in frozen toluene solution at 80 K together with the best fit to the data (solid black or red line). The obtained relaxation times are indicated.

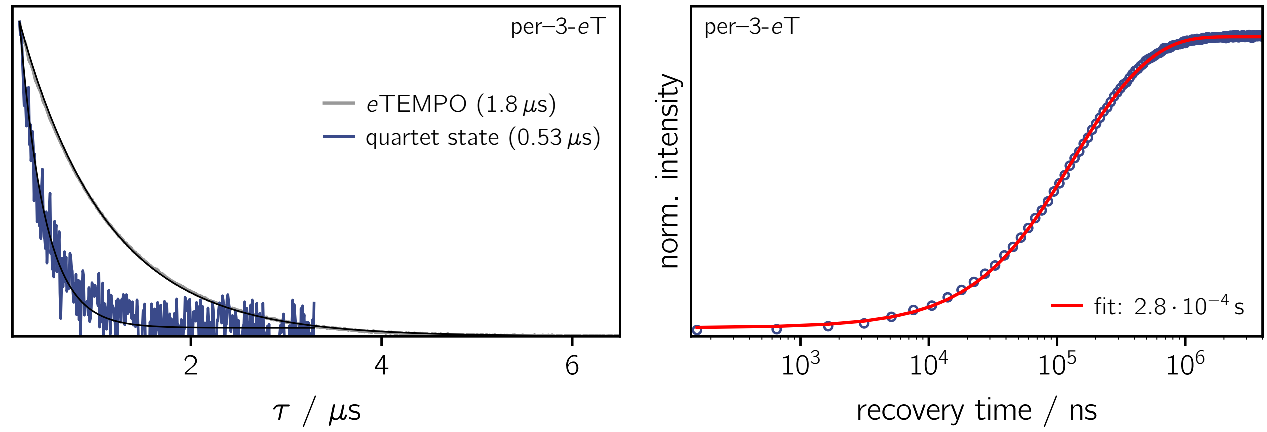

Figure S34: Spin echo decay (*left*) and saturation recovery (*right*) data recorded for per–3-*e*TEMPO in frozen toluene solution at 80 K together with the best fit to the data (solid black or red line). The obtained relaxation times are indicated.

Table S8: Overview of the measured phase memory times T_m and stretched exponential factors β obtained from a fit of the experimental spin echo decay of the three dyads.

compound	T_m^{dark} / µs	β^{dark}	$T_m^{quartet}$ / µs	$\beta^{quartet}$
per–1-*e*TEMPO	2.3	1.1	0.91	1.1
per–2-*e*TEMPO	2.6	1.3	0.60	1.0
per–3-*e*TEMPO	1.8	1.0	0.53	1.0

4 Quantum chemical calculations

Unless stated otherwise, all quantum chemical calculations were carried out using ORCA 5.0.3.[25]

4.1 Optimisation of the structures

The structures of the bromoperylenes shown in Figure S35 were optimised at the B3LYP/def2-TZVP level of theory, whereas the structures of the perylene–*e*TEMPO dyads shown in Figure S37 were optimised at the BP86/def2-TZVP level of theory.[26] The calculations were accelerated with the RIJCOSX approximation using the def2/J auxiliary basis set.[27]

The torsion angles between the naphthalene subunits of the optimised (bromoperylene) structures were determined in order to receive additional information about the influence of the substitution in the peri (3), ortho (2), or bay (1) position on the planarity of the perylene core. Slight distortions are found for the peri and ortho substituted perylenes (peri: 1.4° and ortho: 6.9°). As expected, the bay substituted perylene shows a stronger distortion of 24°, which results from the greater steric repulsion in this position.

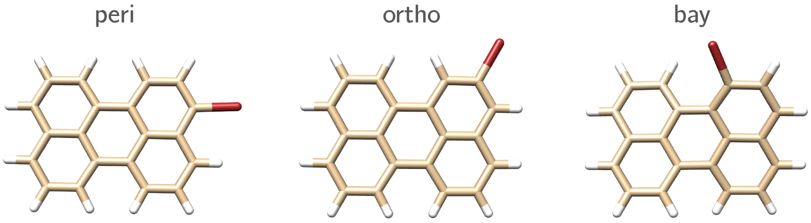

Figure S35: Structures of the bromoperylene dyads calculated at the B3LYP/def2-TZVP level of theory.

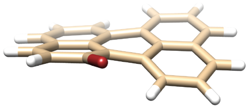

Figure S36: Side view of the optimised structure of 1-bromoperylene. The naphthalene subunits of the perylene are relatively strongly tilted compared to the other two dyads due to the stronger steric repulsion in the bay position.

4.2 Transition dipole moments of bromoperylenes

The transition dipole moments of the bromoperylenes were calculated using TDDFT at the CAM-B3LYP/def2-TZVP level of theory. The calculations were accelerated with the RIJCOSX approximation using the def2/J auxiliary basis set. The transition dipole moments (shown in the main part, Figure 2) are related to the S_1 transitions.

4.3 Rotational conformers and barriers

The energies of the rotational conformers of the perylene–*e*TEMPO dyads and their barriers were calculated at the BP86/def2-TZVP level of theory. The results are shown in the main part (Figure 6). A relaxed surface scan was applied in order to find the ground state conformers, where the torsion angle between the chromophore and the radical was altered by 5° in each cycle. In order to obtain the energetically lowest structures on the potential surface, the structures corresponding to the calculated minima found during the scan were reoptimised at the BP86/def2-TZVP level of theory without a torsion angle constraint between chromophore and radical. The structures obtained this way are shown in Figure S37.

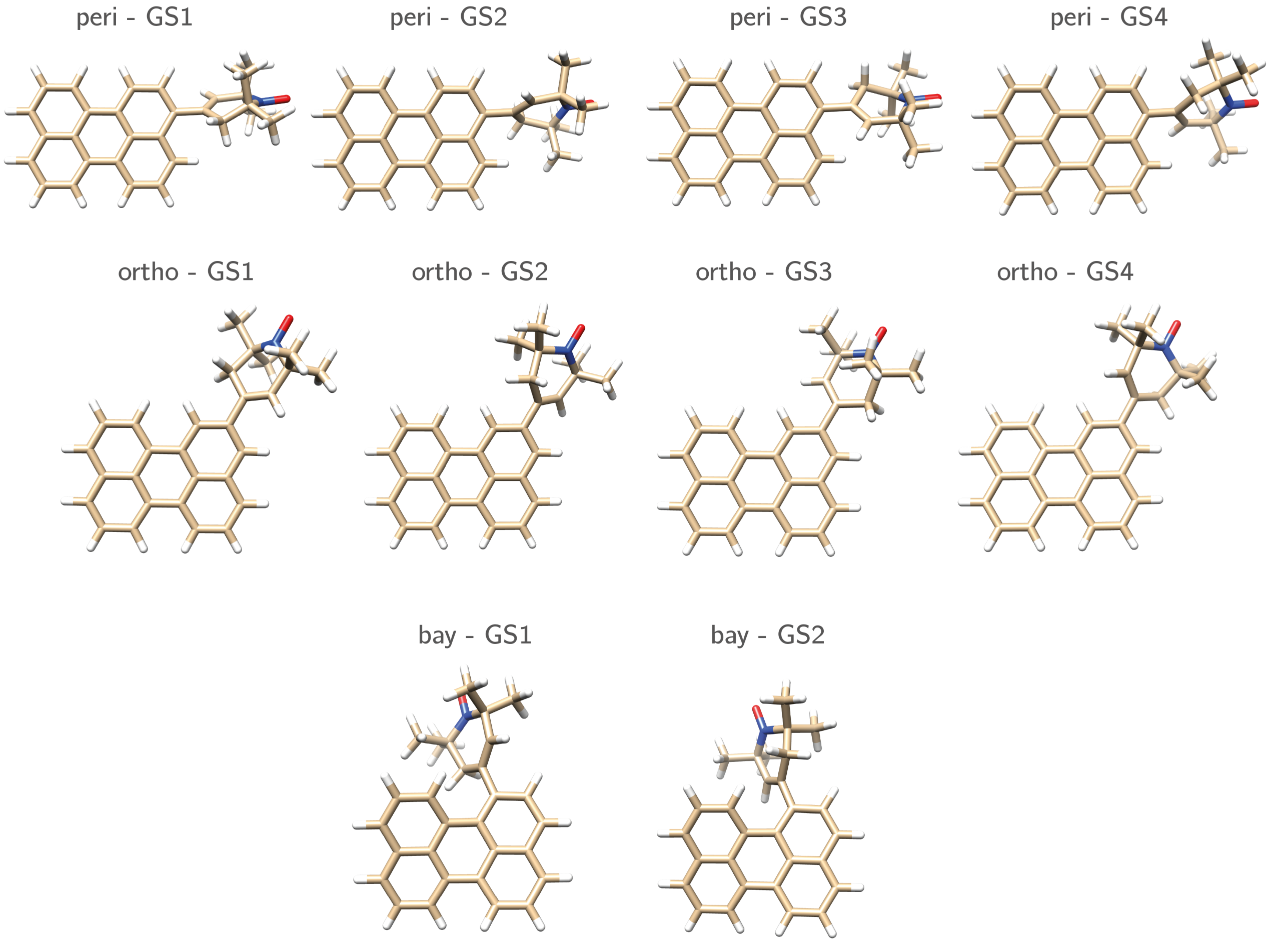

Figure S37: Structures of the perylene–*e*TEMPO dyads calculated at the BP86/def2-TZVP level of theory.

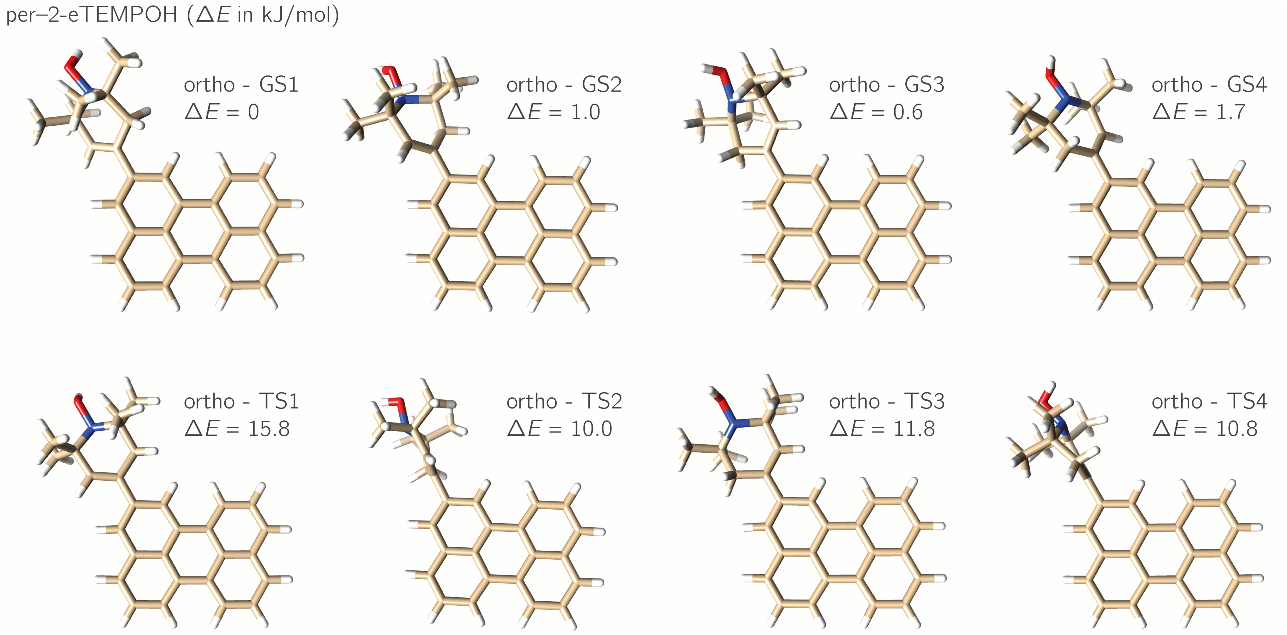

Figure S38: Ground and transition state structures of per–2-*e*TEMPOH dyads calculated at the ωB97XD/6-311G(d,p) level of theory.

Four ground state structures could be found for per–3-*e*TEMPO and per–2-*e*TEMPO, whereas only two ground state structures were found for per–1-*e*TEMPO.

The rotation energy barriers for the reduced versions of per–2-*e*TEMPO and per–1-*e*TEMPO, namely per–2-*e*TEMPOH and per–1-*e*TEMPOH, were calculated as described above but using the Gaussian

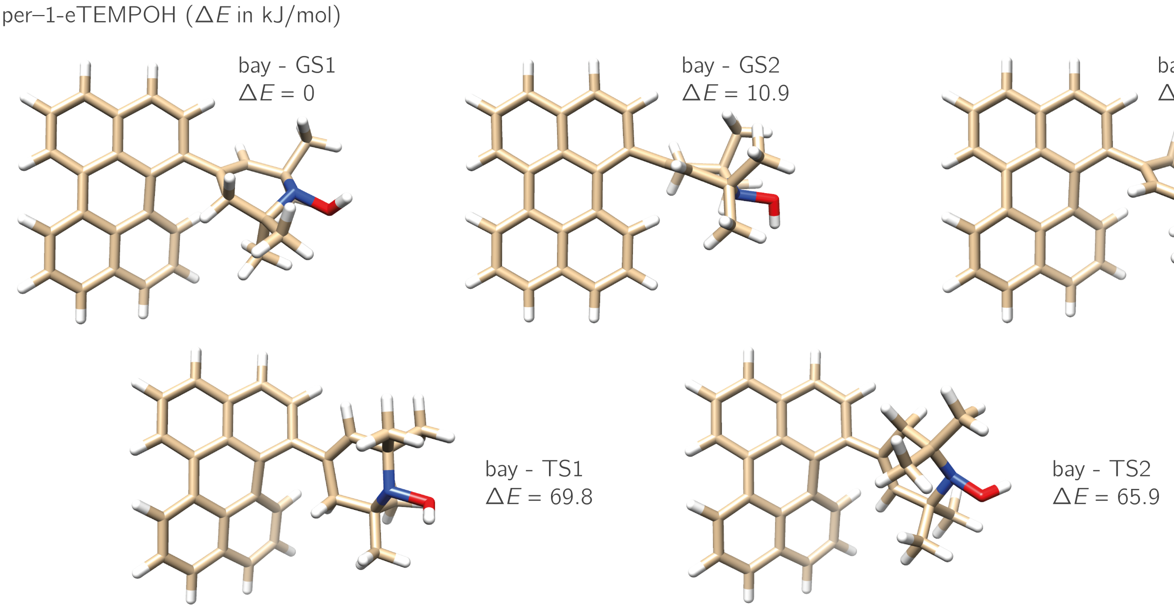

Figure S39: Ground and transition state structures of per–1-*e*TEMPOH dyads calculated at the ωB97XD/6-311G(d,p) level of theory.

software suite[28] at the ωB97XD/6-311G(d,p) level of theory[29–31] using a polarisable continuum model (PCM)[32] for toluene to better compare with the experimental NMR data. The energy differences of the transition states were calculated by optimising the corresponding least favourable geometries found based on the rotational scan. The optimised transition state structures were confirmed to have a unique imaginary frequency that corresponded, in all cases, to the expected molecular motion. The optimised geometries and relative energies for per–2-*e*TEMPOH and per–1-*e*TEMPOH are shown in Figure S38 and Figure S39 respectively.

The obtained calculated barriers for per–1-*e*TEMPOH are in excellent agreement with the measured values (62.9 and 62.2 kJ/mol vs 69.8 kJ/mol), confirming the presence of several rotational isomers and that these are accessible under the experimental conditions. The values obtained for per–2-*e*TEMPOH do not match because the half-boat isomerisation of the TEMPOH unit (detected by NMR) shows a higher barrier than the rotation around the bond connecting perylene and *e*TEMPOH. Thus, the rotation, targeted here, is not observed by NMR, which is expected given the small barrier height.

4.4 Excited state exchange coupling calculations

The excited state exchange couplings were calculated at the DDCI2/def2-SVP level of theory. Here, the uncontracted variant of the DDCI2 method was used. Furthermore, the calculations were accelerated with the RIJCOSX approximation using the def2/J and def2-SVP/C auxiliary basis sets. The reference wavefunctions were obtained by a state-averaged CASSCF(3,3) calculation, whereby all possible states (one quartet state and eight doublet states) were equally weighted. The initial active orbitals were obtained from a previous TDDFT calculation.

The values for J_{TR} listed in Tables S9 to S11 were calculated by using the DDCI2 energies of the quartet state and the trip-doublet state:

$$J_{\mathrm{TR}} = \frac{2}{3}(E_{\mathrm{D}_1} - E_{\mathrm{Q}_0}). \tag{S6}$$

Tables S9 to S11 summarise the relative energies of the rotational conformers of per–1-*e*TEMPO,

per–2-*e*TEMPO, and per–3-*e*TEMPO, their calculated J_{TR} values, as well as the occupation probabilities of their respective ground states. The occupation probabilities of the ground states were calculated both for room temperature (295 K) and the freezing point of the solvent toluene (178 K) according to

$$P_i = \frac{\exp\left(-\frac{E_i}{k_B T}\right)}{\sum_i \exp\left(-\frac{E_i}{k_B T}\right)} \tag{S7}$$

where k_B is the Boltzmann constant and E_i is the relative energy of the ith state with respect to the global minimum. The Boltzmann-weighted average value for J_{TR} at these temperatures is given by

$$\langle J \rangle = \frac{\sum_i J_i \exp\left(-\frac{E_i}{k_B T}\right)}{\sum_i \exp\left(-\frac{E_i}{k_B T}\right)}. \tag{S8}$$

Table S9: Relative energies of the rotational conformers of per–1-*e*TEMPO, their calculated J_{TR} values, and occupation probabilities of the respective ground states at 295 K and 178 K. The Boltzmann-weighted average value for J_{TR} amounts to $\langle J_{TR} \rangle = 3.12\,cm^{-1}$ at 295 K and $\langle J_{TR} \rangle = 3.04\,cm^{-1}$ at 178 K.

ground state	ΔE / kJ mol^{-1}	J_{TR} / cm^{-1}	P(295 K)	P(178 K)
**1**	0	2.94	0.754	0.865
**2**	2.75	3.68	0.246	0.135

Table S10: Relative energies of the rotational conformers of per–2-*e*TEMPO, their calculated J_{TR} values, and occupation probabilities of the respective ground states at 295 K and 178 K. The Boltzmann-weighted average value for J_{TR} amounts to $\langle J_{TR} \rangle = 0.303\,cm^{-1}$ at 295 K and $\langle J_{TR} \rangle = 0.285\,cm^{-1}$ at 178 K.

ground state	ΔE / kJ mol^{-1}	J_{TR} / cm^{-1}	P(295 K)	P(178 K)
**1**	1.14	0.363	0.252	0.236
**2**	2.34	0.422	0.155	0.105
**3**	0	0.209	0.401	0.509
**4**	1.81	0.325	0.192	0.150

Table S11: Relative energies of the rotational conformers of per–3-*e*TEMPO, their calculated J_{TR} values, and occupation probabilities of the respective ground states at 295 K and 178 K. The Boltzmann-weighted average value for J_{TR} amounts to $\langle J_{TR} \rangle = 2.98\,cm^{-1}$ at 295 K and $\langle J_{TR} \rangle = 2.98\,cm^{-1}$ at 178 K.

ground state	ΔE / kJ mol^{-1}	J_{TR} / cm^{-1}	P(295 K)	P(178 K)
**1**	0.769	2.35	0.231	0.214
**2**	0.357	1.50	0.273	0.283
**3**	1.38	4.18	0.180	0.142
**4**	0	4.05	0.316	0.361

5 NMR spectra

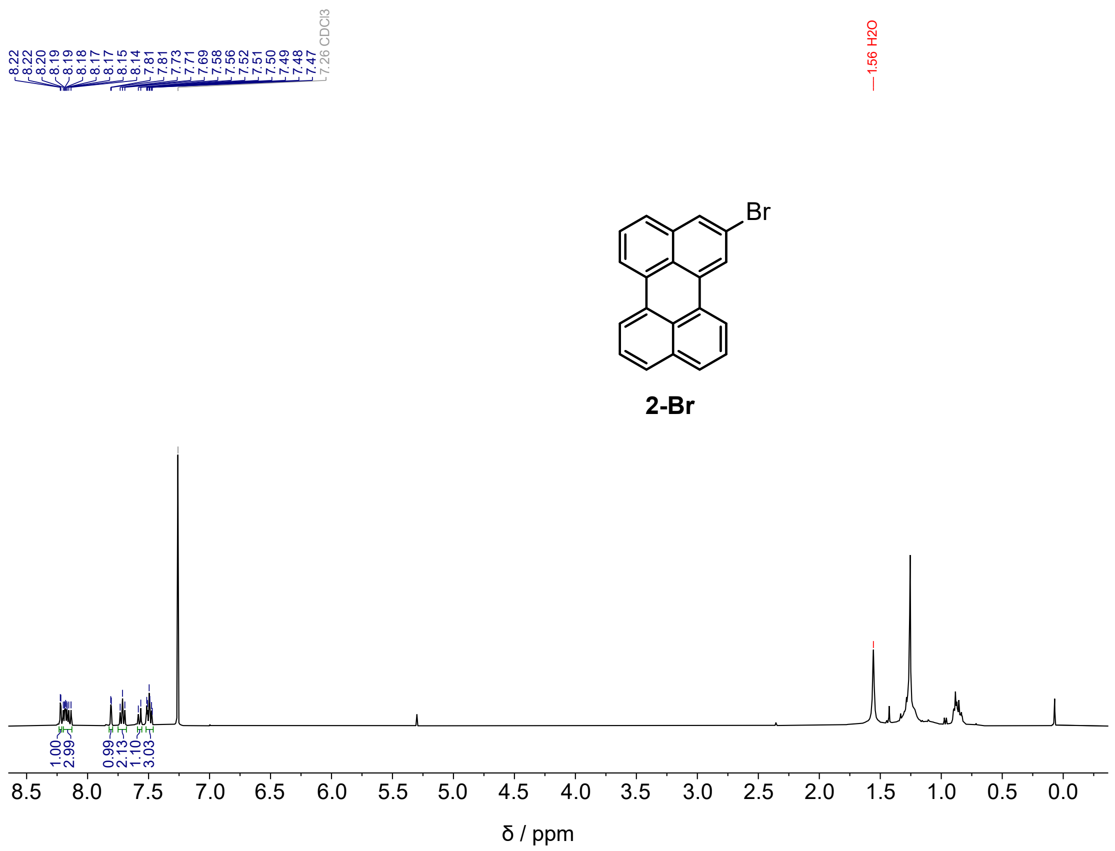

Figure S40: ^{1}H NMR of 2-Br (400 MHz, 298 K, $CDCl_3$).

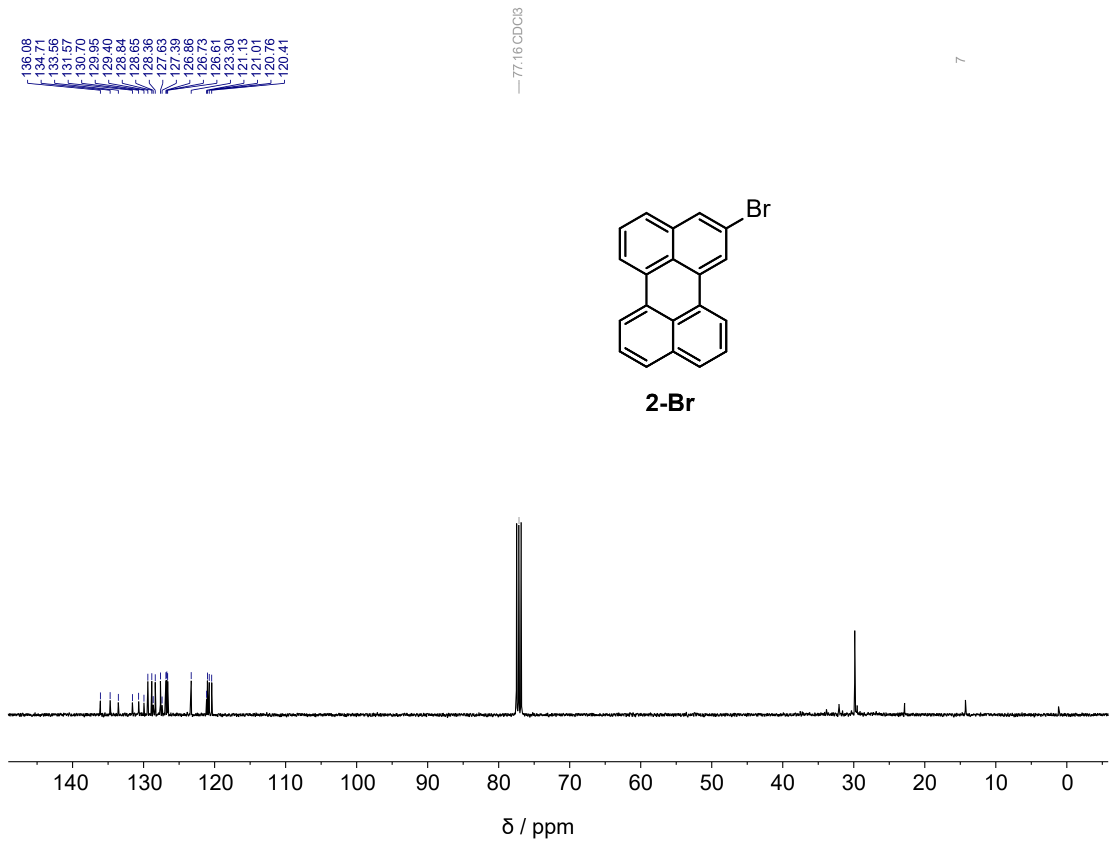

Figure S41: ^{13}C NMR of 2-Br (101 MHz, 298 K, $CDCl_3$).

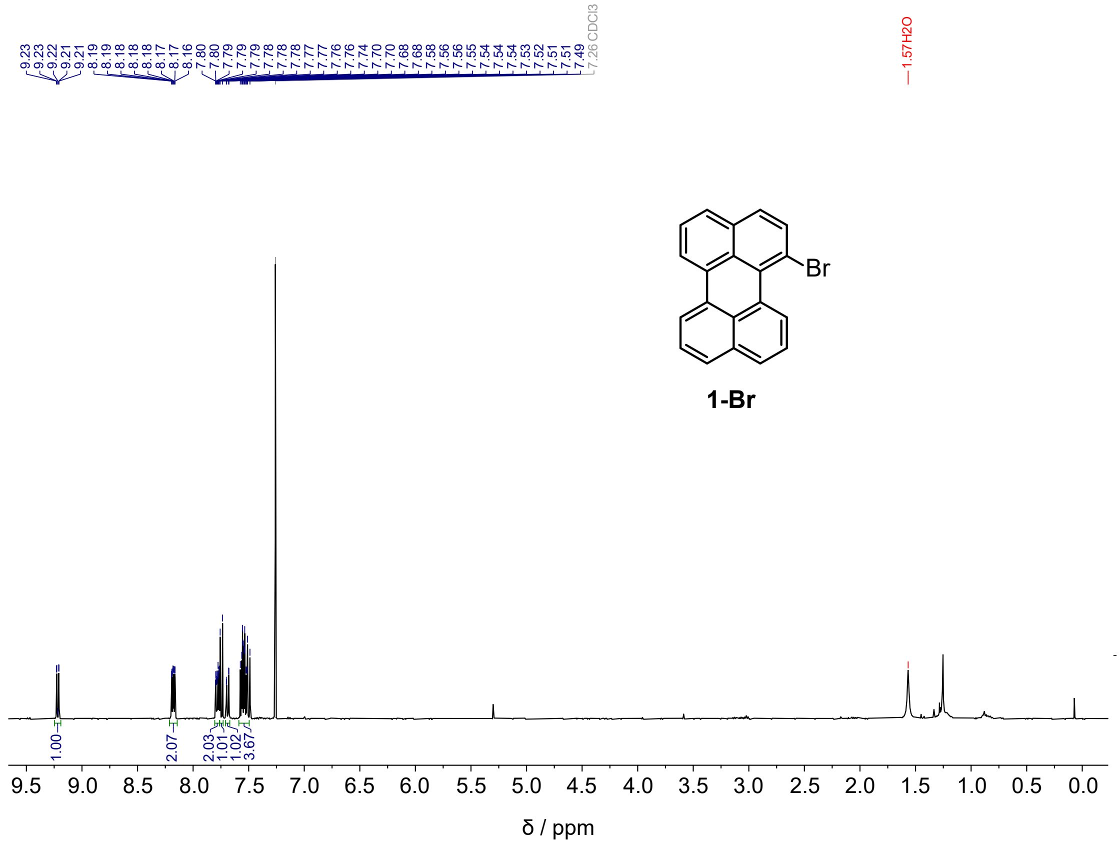

Figure S42: ^{1}H NMR of 1-Br (400 MHz, 298 K, $CDCl_3$).

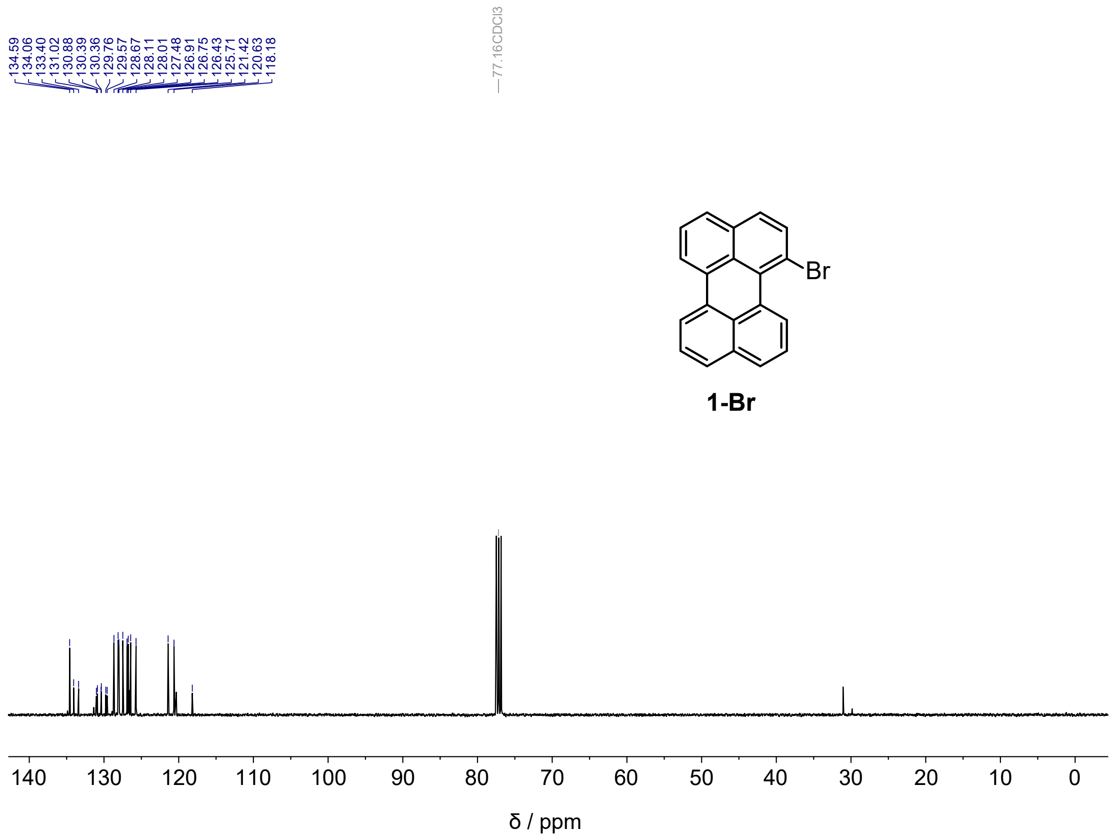

Figure S43: ^{13}C NMR of 1-Br (101 MHz, 298 K, $CDCl_3$).

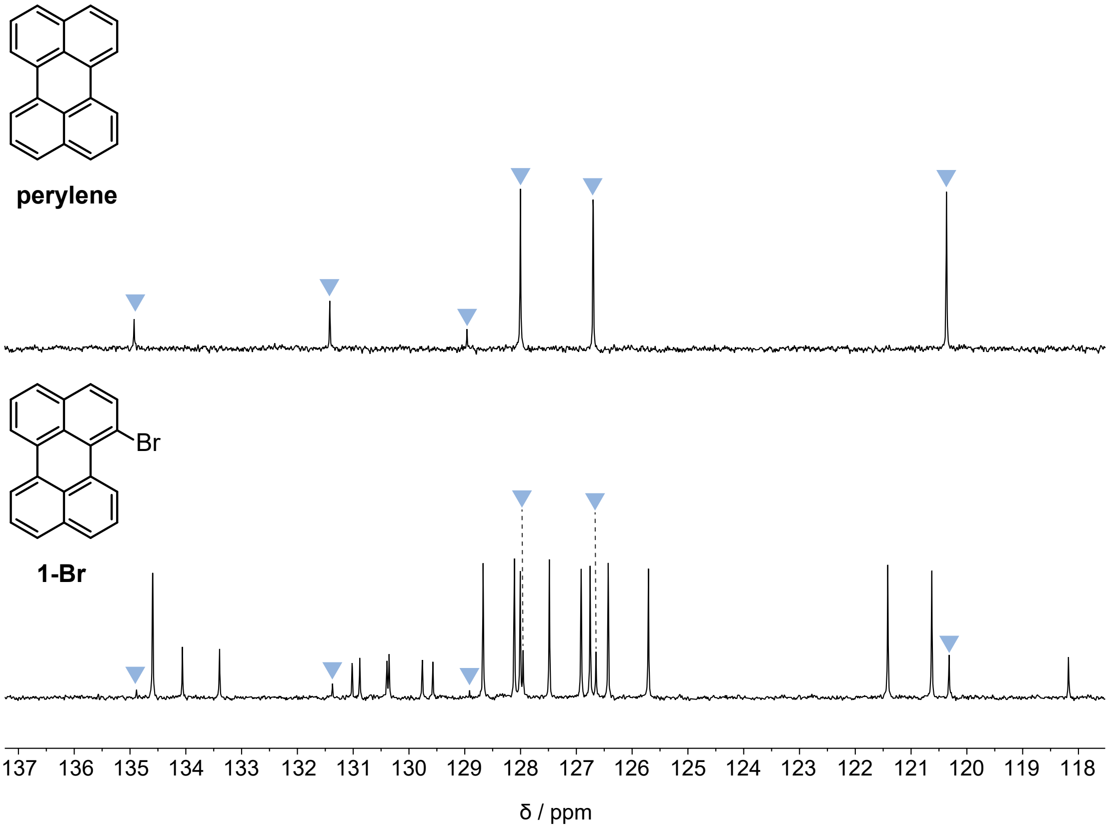

Figure S44: ^{13}C NMR comparison of perylene and 1-Br (101 MHz, 298 K, $CDCl_3$).

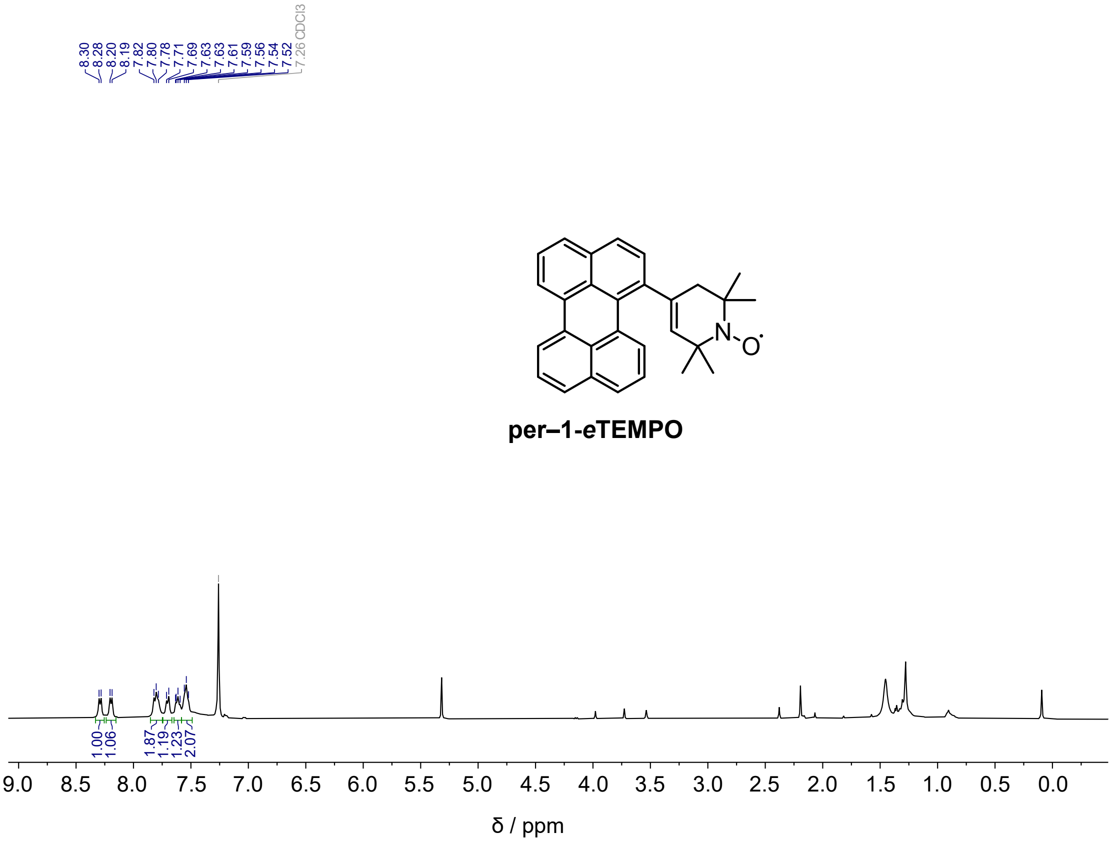

Figure S45: ^{1}H NMR of per–1-*e*TEMPO (400 MHz, 298 K, $CDCl_3$).

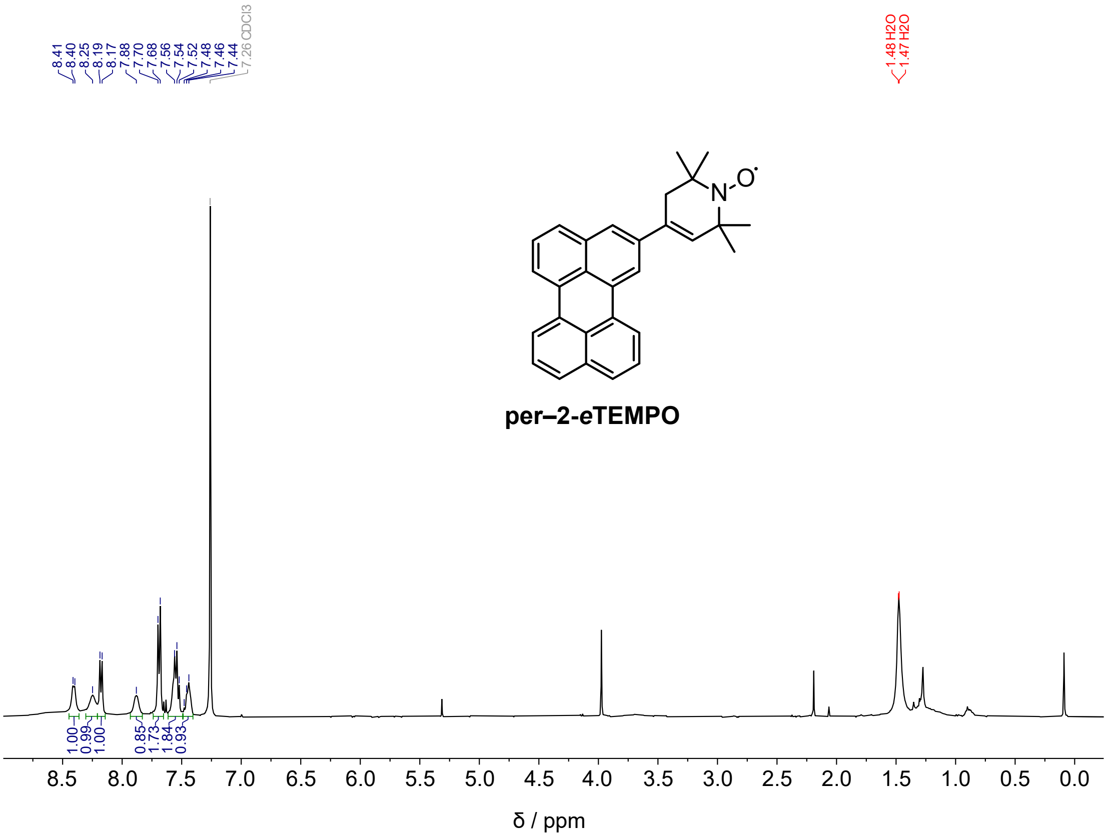

Figure S46: ^{1}H NMR of per–2-*e*TEMPO (400 MHz, 298 K, $CDCl_3$).

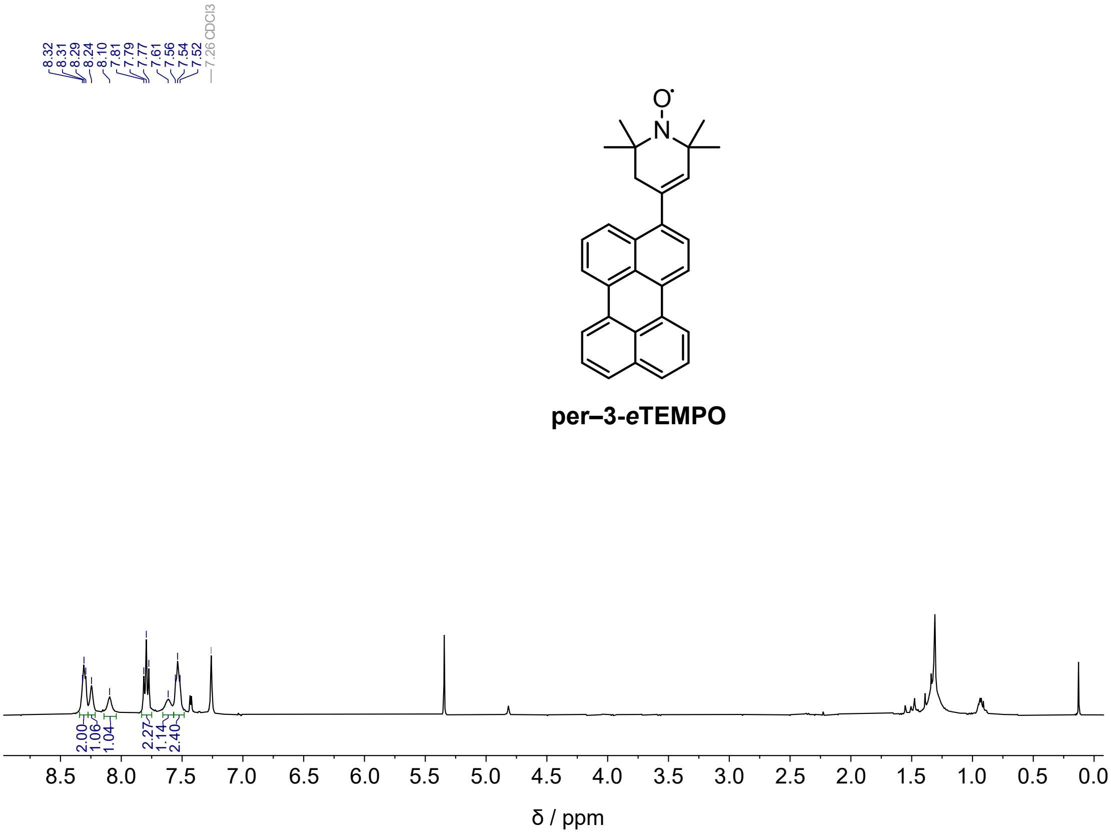

Figure S47: ^{1}H NMR of per–3-*e*TEMPO (400 MHz, 298 K, $CDCl_3$).

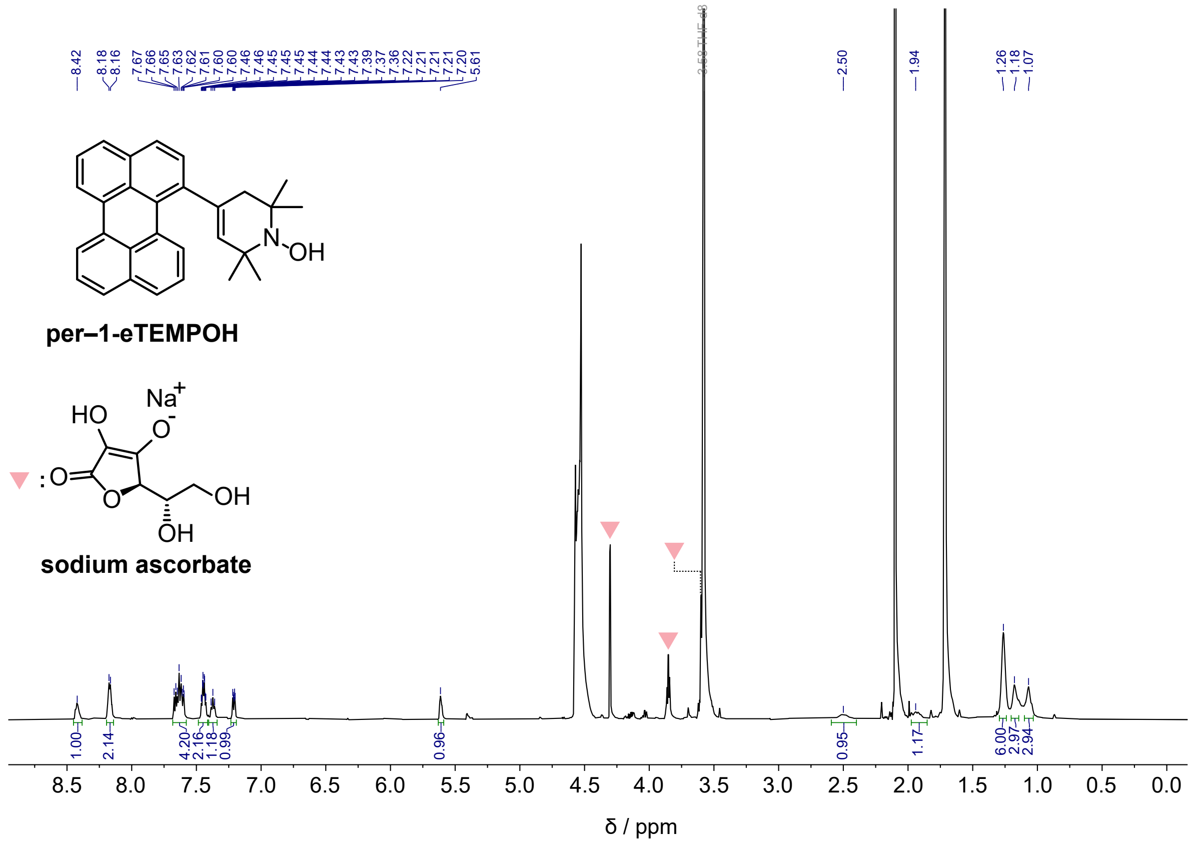

Figure S48: ^{1}H NMR of per–1-*e*TEMPOH (400 MHz, 298 K, THF-d_8).

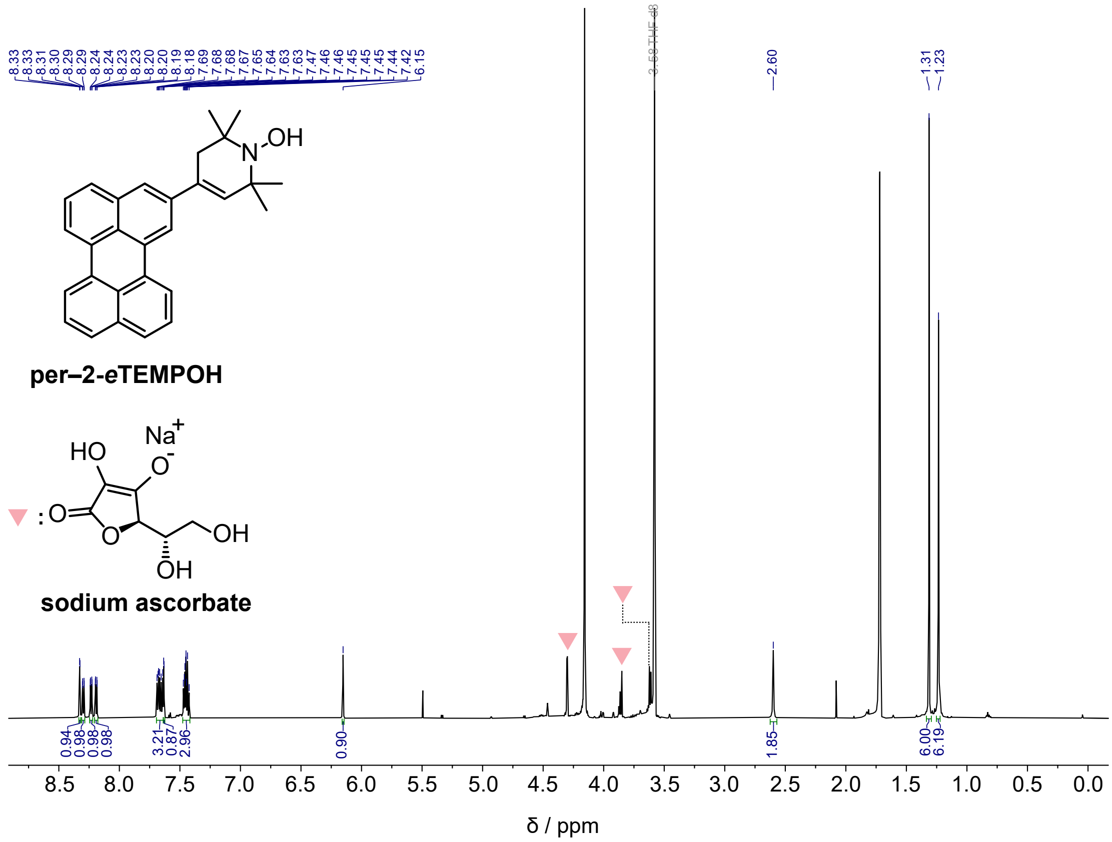

Figure S49: ^{1}H NMR of per–2-*e*TEMPOH (400 MHz, 298 K, THF-d_8).

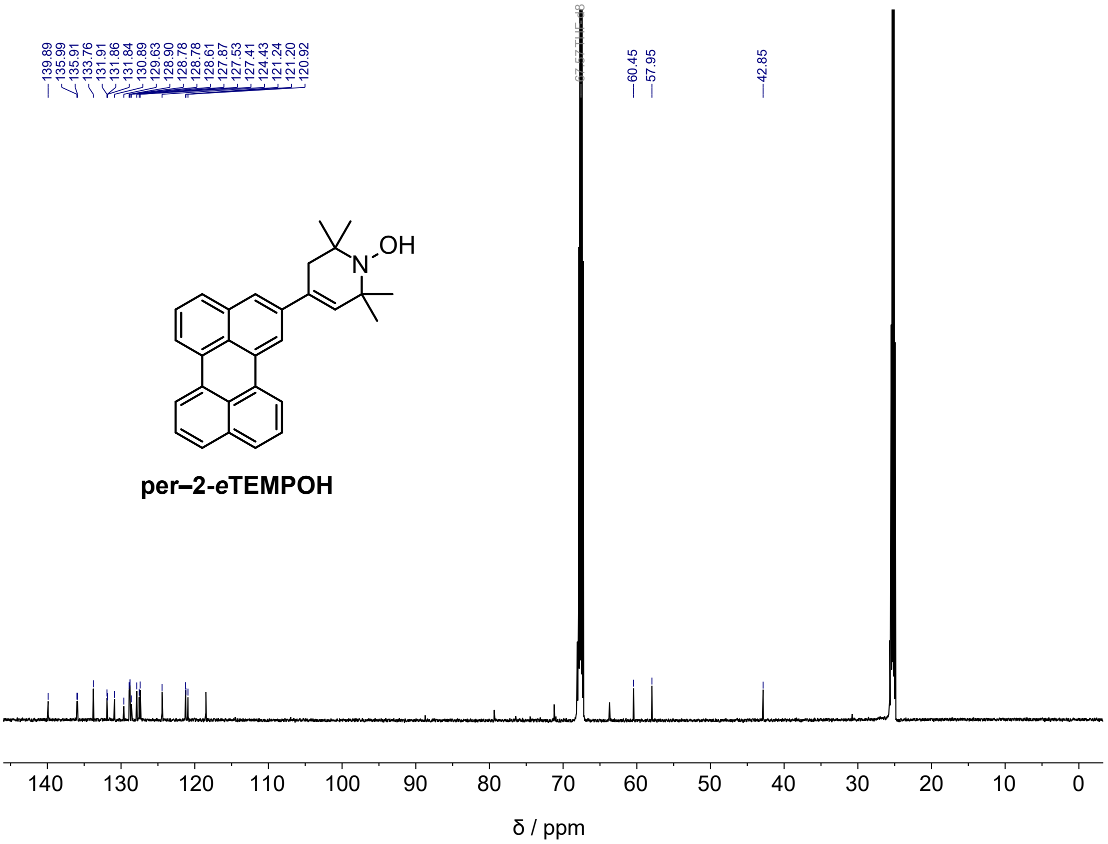

Figure S50: ^{13}C NMR of per–2-*e*TEMPOH (151 MHz, 298 K, THF-d_8).

2D NMR spectra

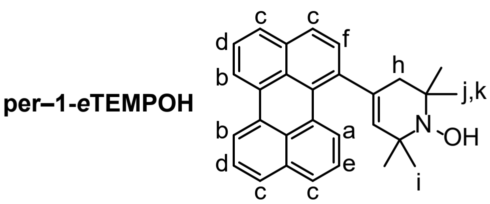

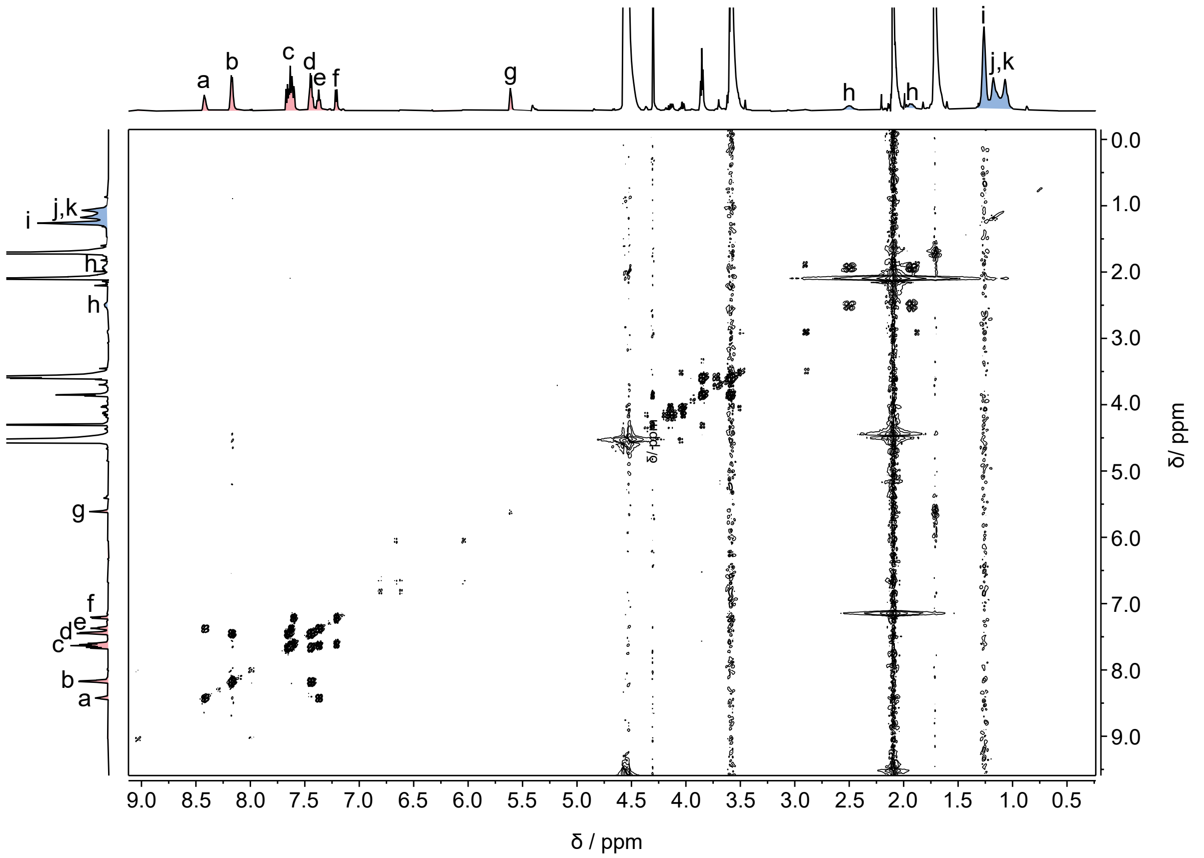

Figure S51: ^{1}H-^{1}H COSY of per–1-*e*TEMPOH (600 MHz, 298 K, THF-d_8).

per–1-eTEMPOH

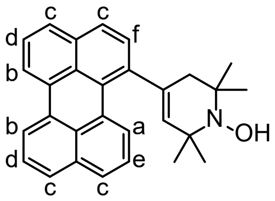

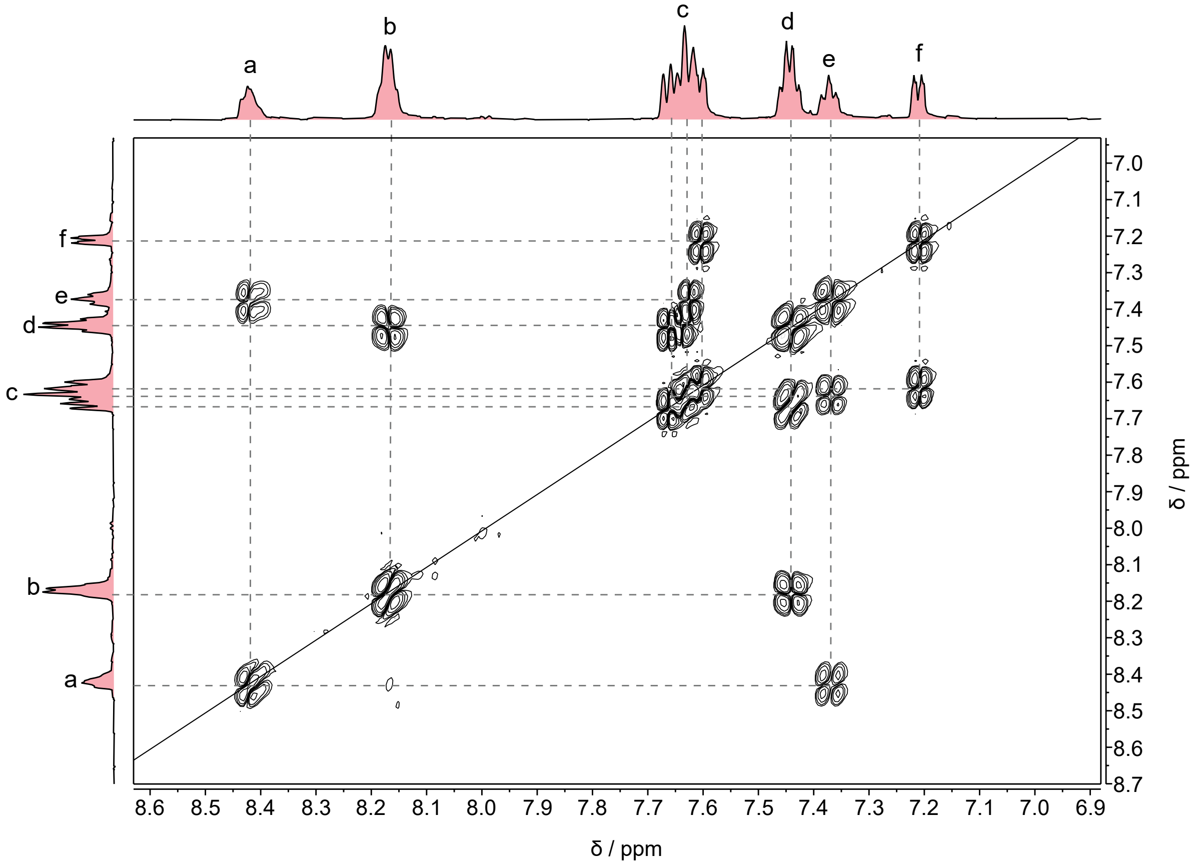

Figure S52: ^{1}H-^{1}H COSY of per–1-*e*TEMPOH (600 MHz, 298 K, THF-d_8, aromatic region).

per–1-*e*TEMPOH

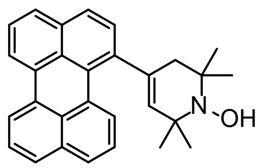

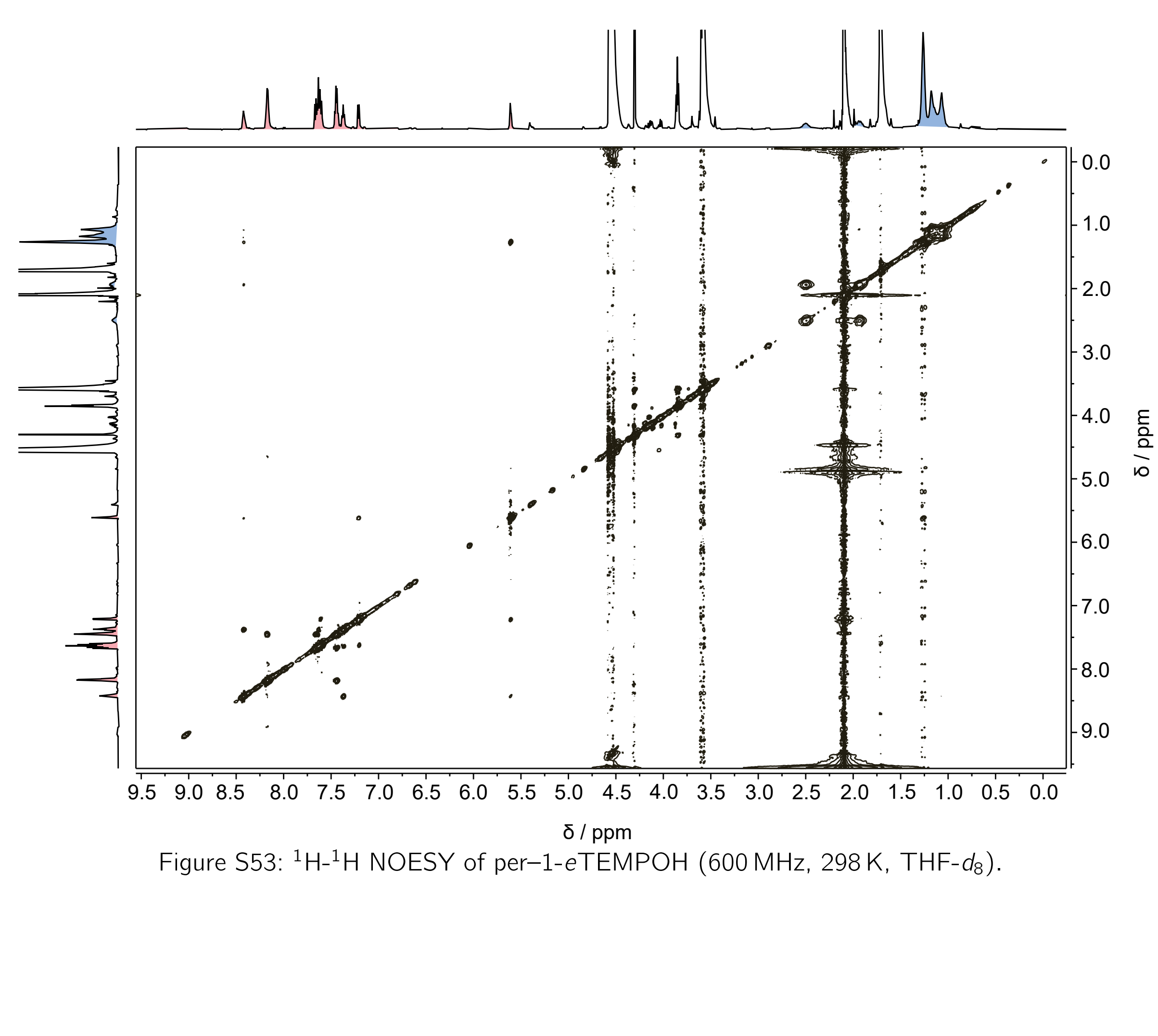

Figure S53: ^{1}H-^{1}H NOESY of per–1-*e*TEMPOH (600 MHz, 298 K, THF-d_8).

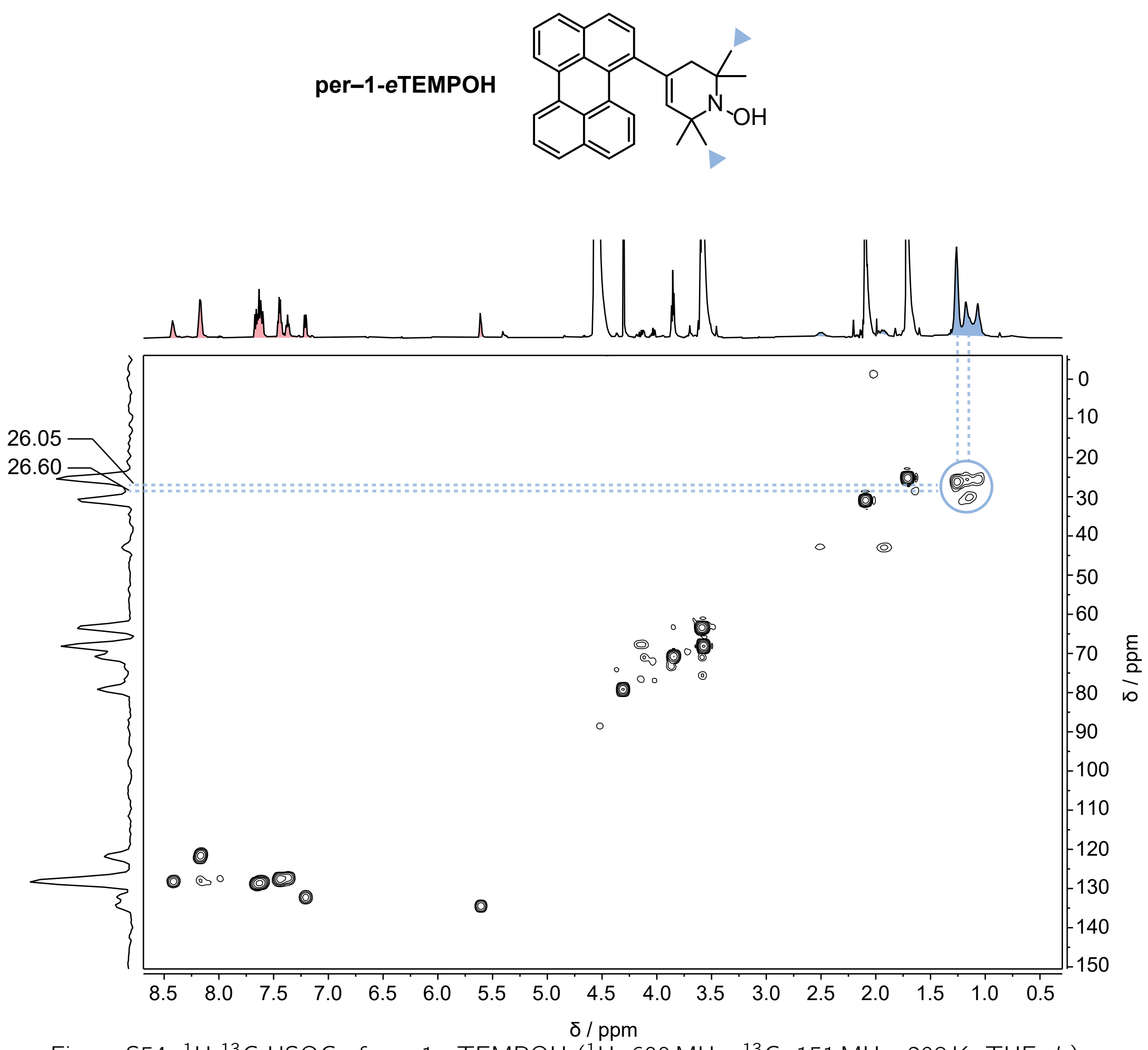

Figure S54: ^{1}H-^{13}C HSQC of per–1-*e*TEMPOH (^{1}H: 600 MHz; ^{13}C: 151 MHz, 298 K, THF-d_8).

per–1-*e*TEMPOH

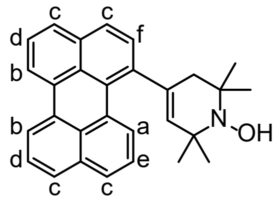

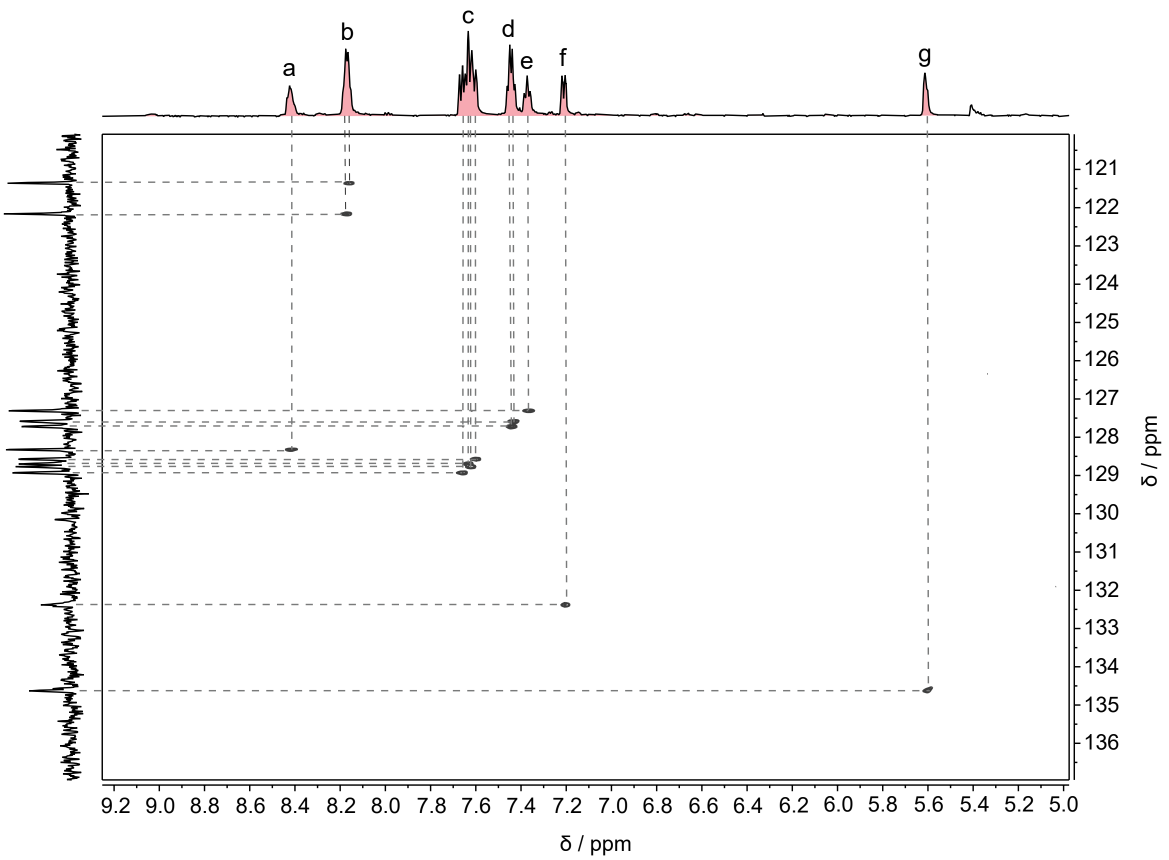

Figure S55: ^{1}H-^{13}C HSQC of per–1-*e*TEMPOH (^{1}H: 600 MHz; ^{13}C: 151 MHz, 298 K, THF-d_8, aromatic region).

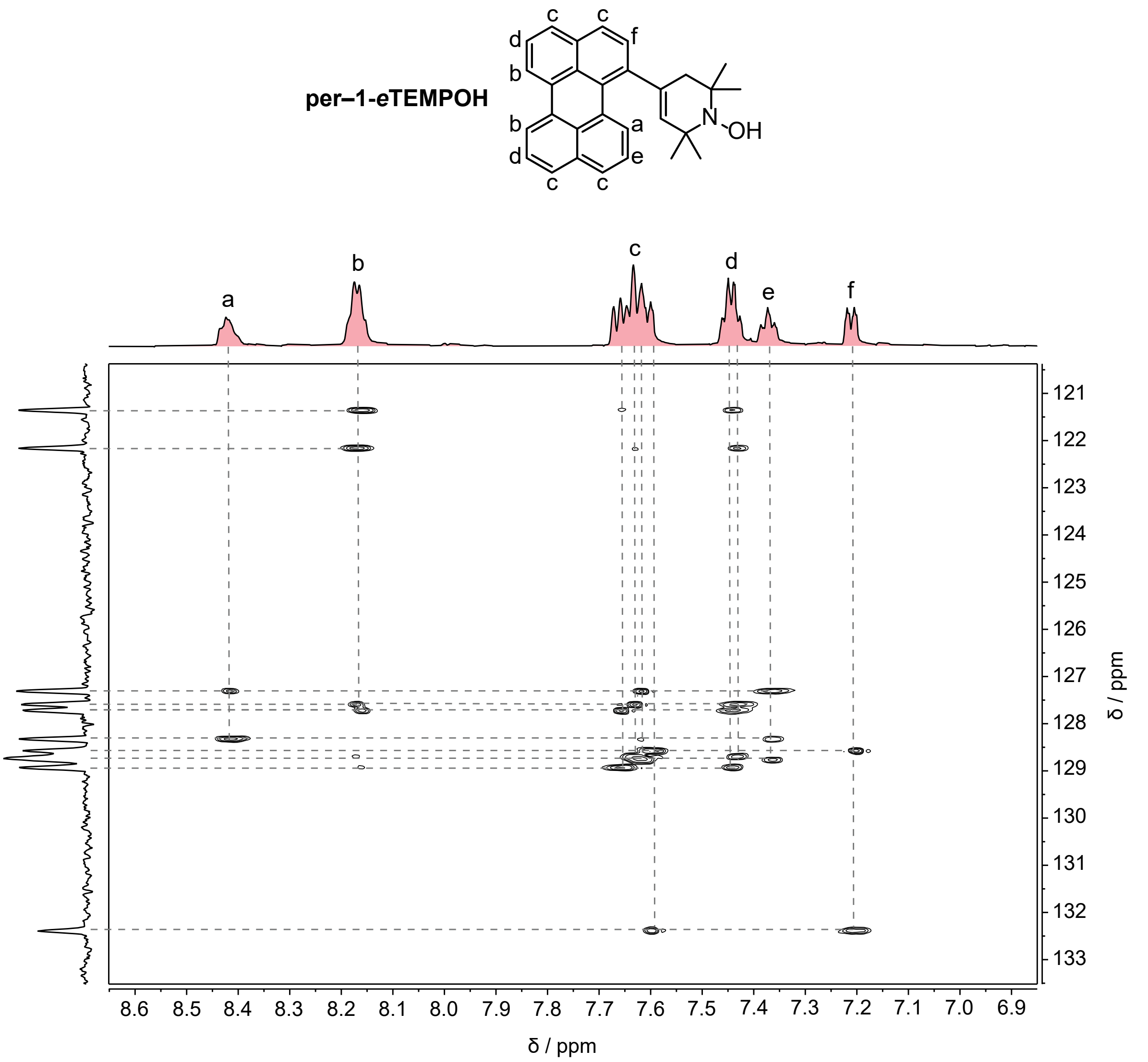

Figure S56: ^{1}H-^{13}C HSQC-TOCSY of per–1-*e*TEMPOH (^{1}H: 600 MHz; ^{13}C: 151 MHz, 298 K, THF-d_8, aromatic region).

per–1-*e*TEMPOH

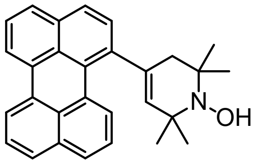

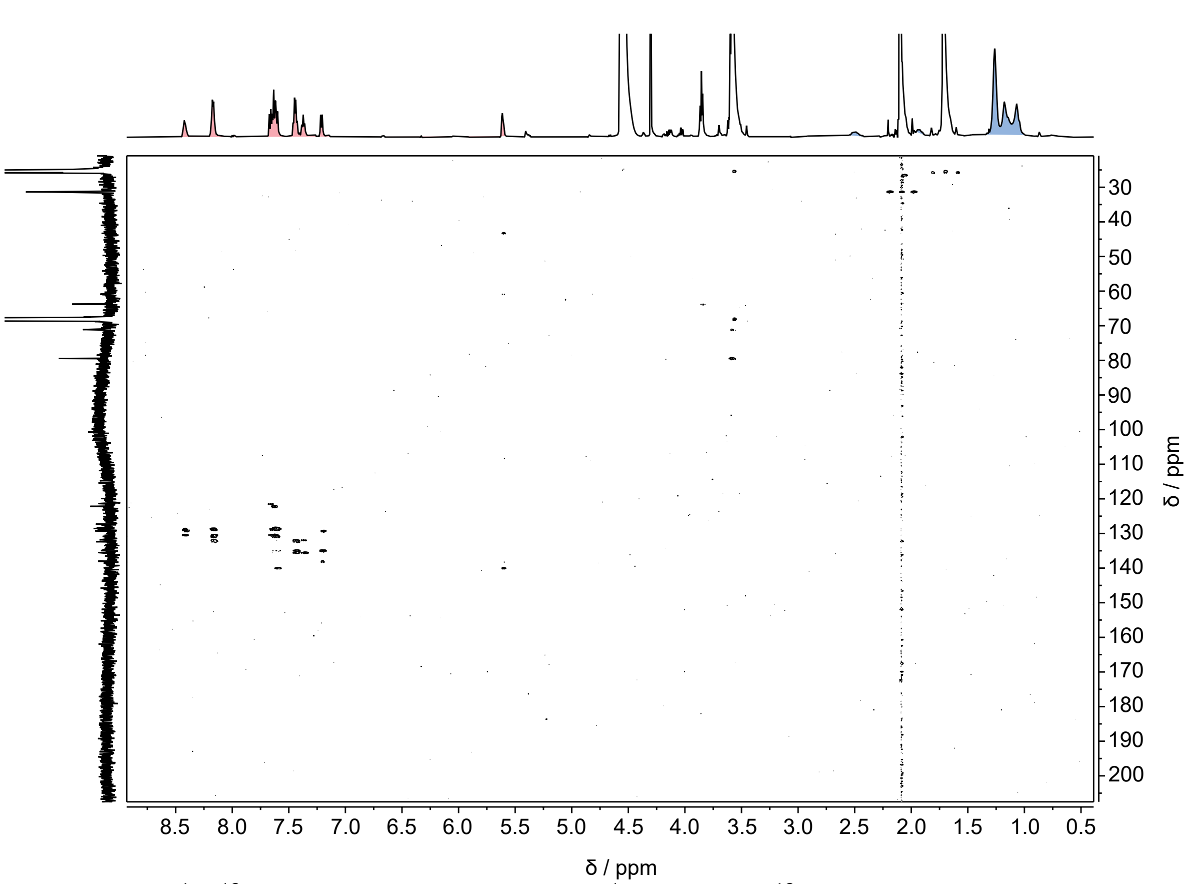

Figure S57: ^{1}H-^{13}C HMBC of per–1-*e*TEMPOH (^{1}H: 600 MHz; ^{13}C: 151 MHz, 298 K, THF-d_8).

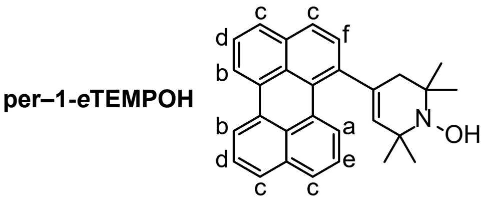

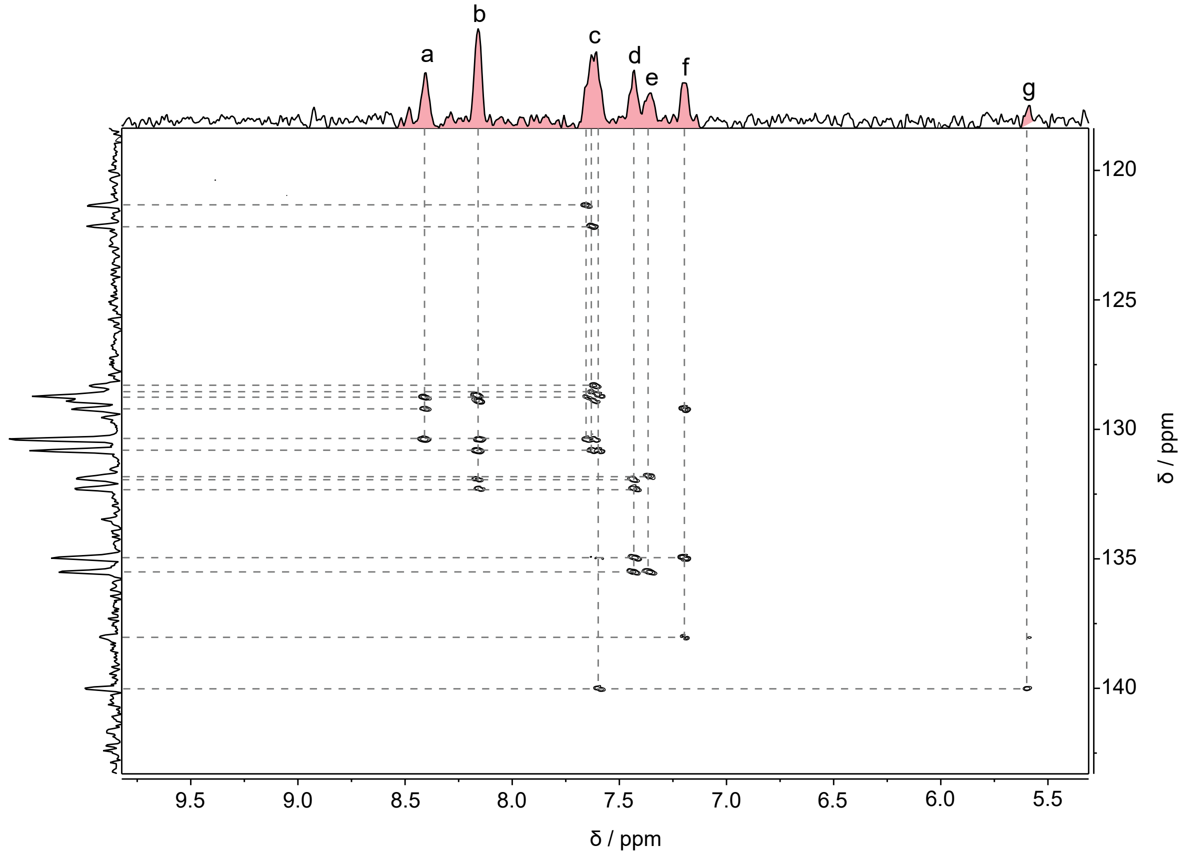

Figure S58: ^{1}H-^{13}C HMBC of per–1-*e*TEMPOH (^{1}H: 600 MHz; ^{13}C: 151 MHz, 298 K, THF-d_8, aromatic region).

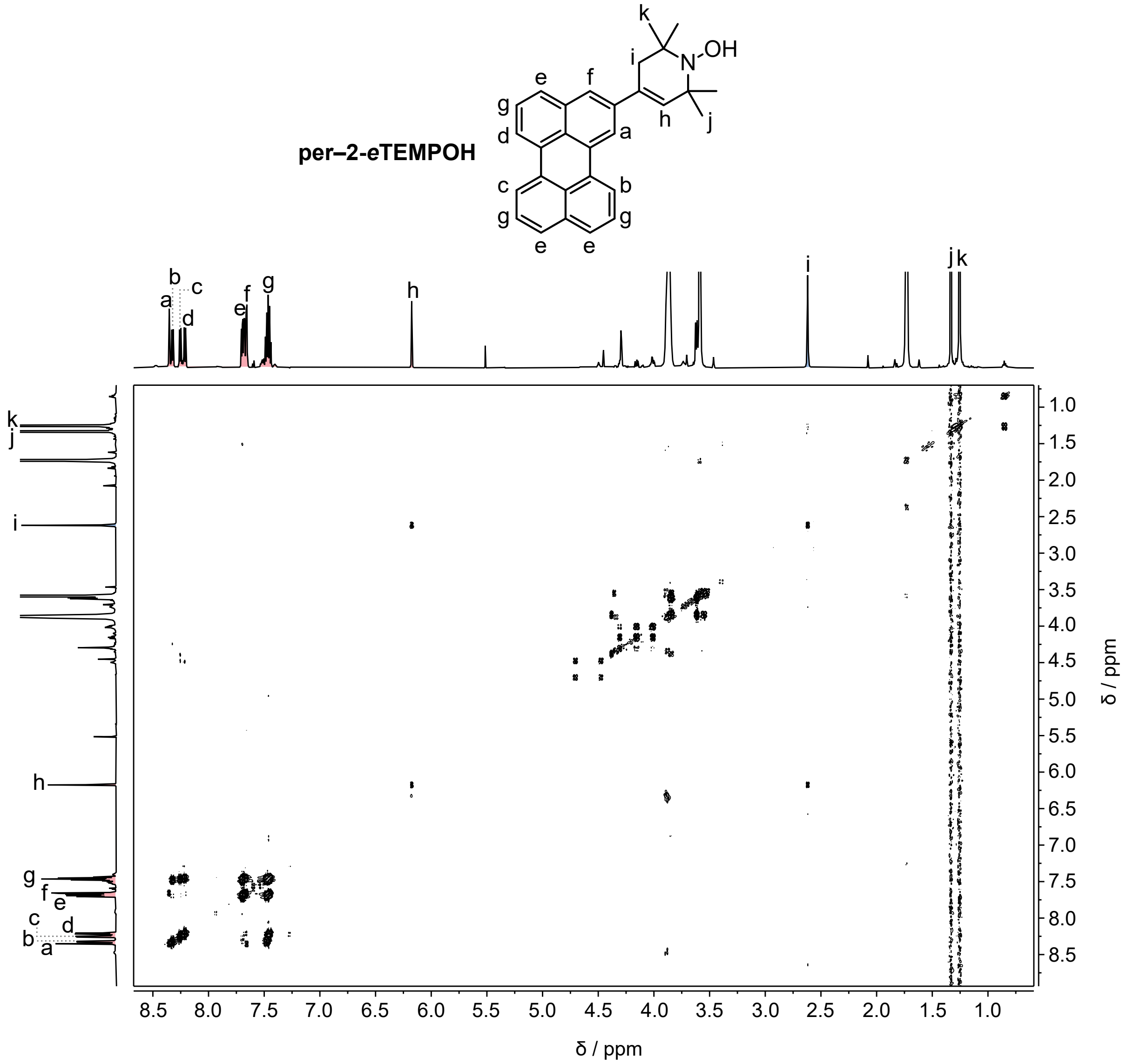

Figure S59: ^{1}H-^{1}H COSY of per–2-*e*TEMPOH (600 MHz, 298 K, THF-d_8).

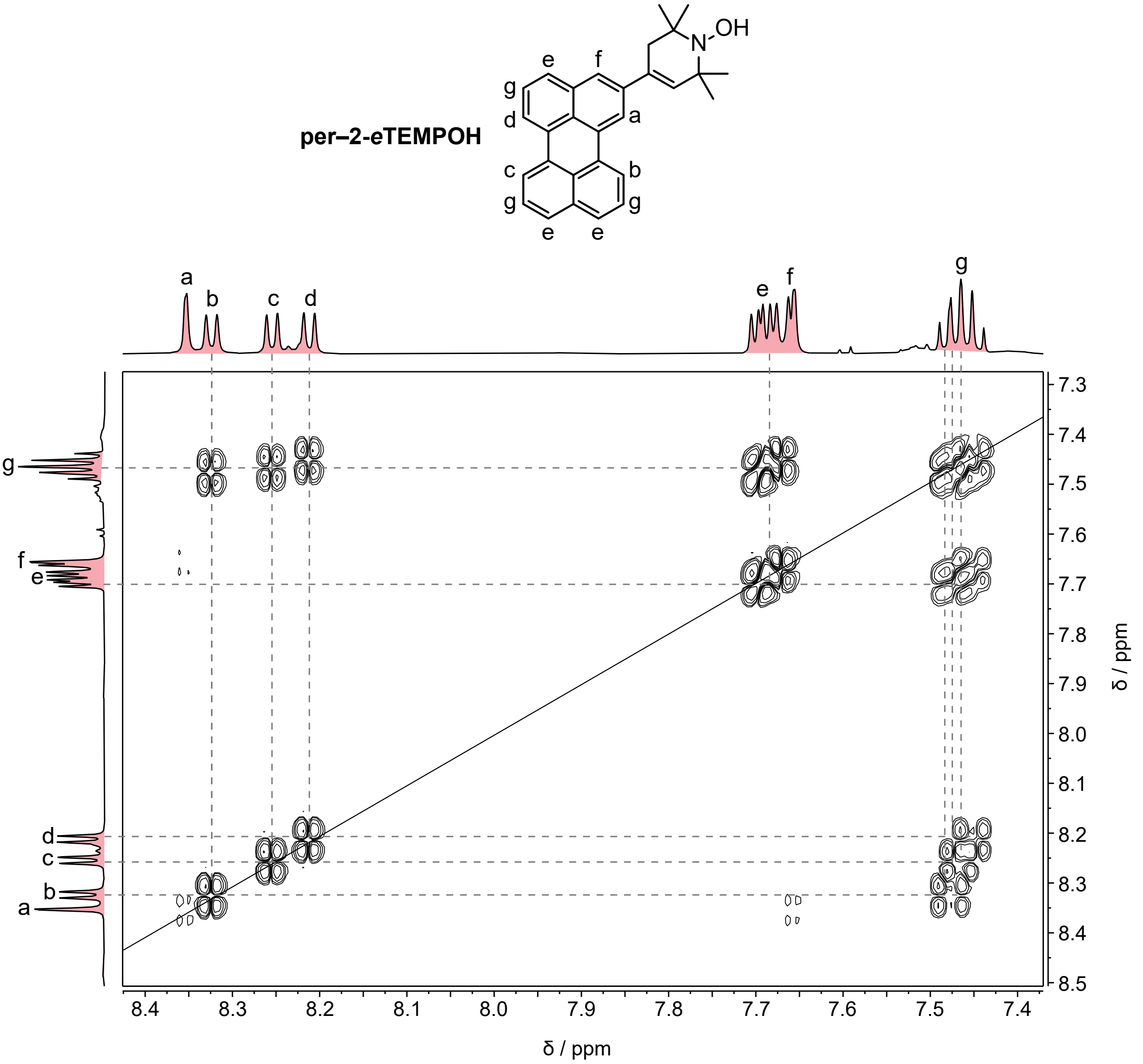

Figure S60: ^{1}H-^{1}H COSY of per–2-*e*TEMPOH (600 MHz, 298 K, THF-d_8, aromatic region).

per–2-eTEMPOH

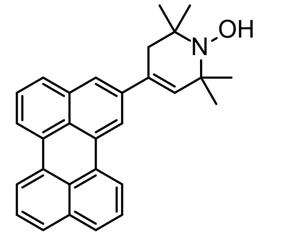

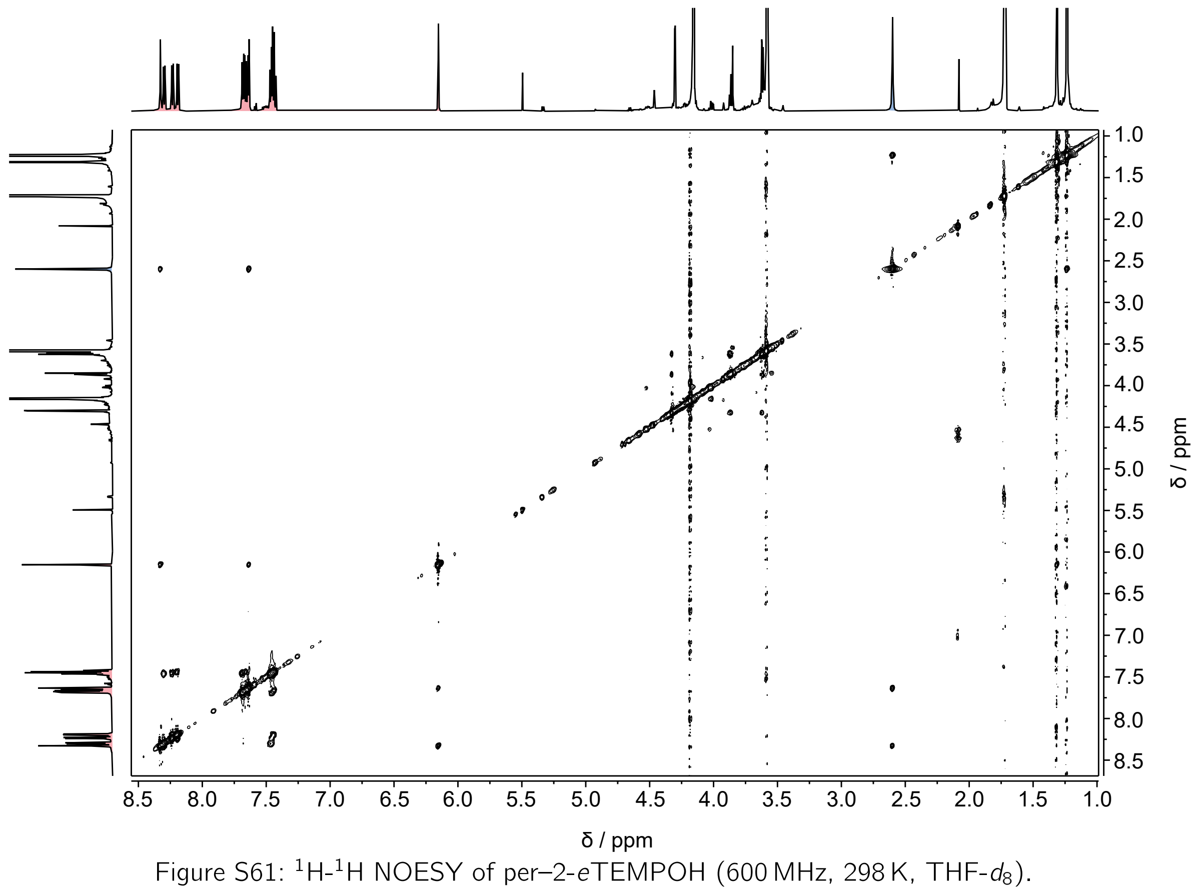

Figure S61: ^{1}H-^{1}H NOESY of per–2-*e*TEMPOH (600 MHz, 298 K, THF-d_8).

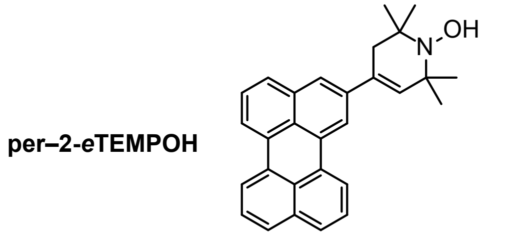

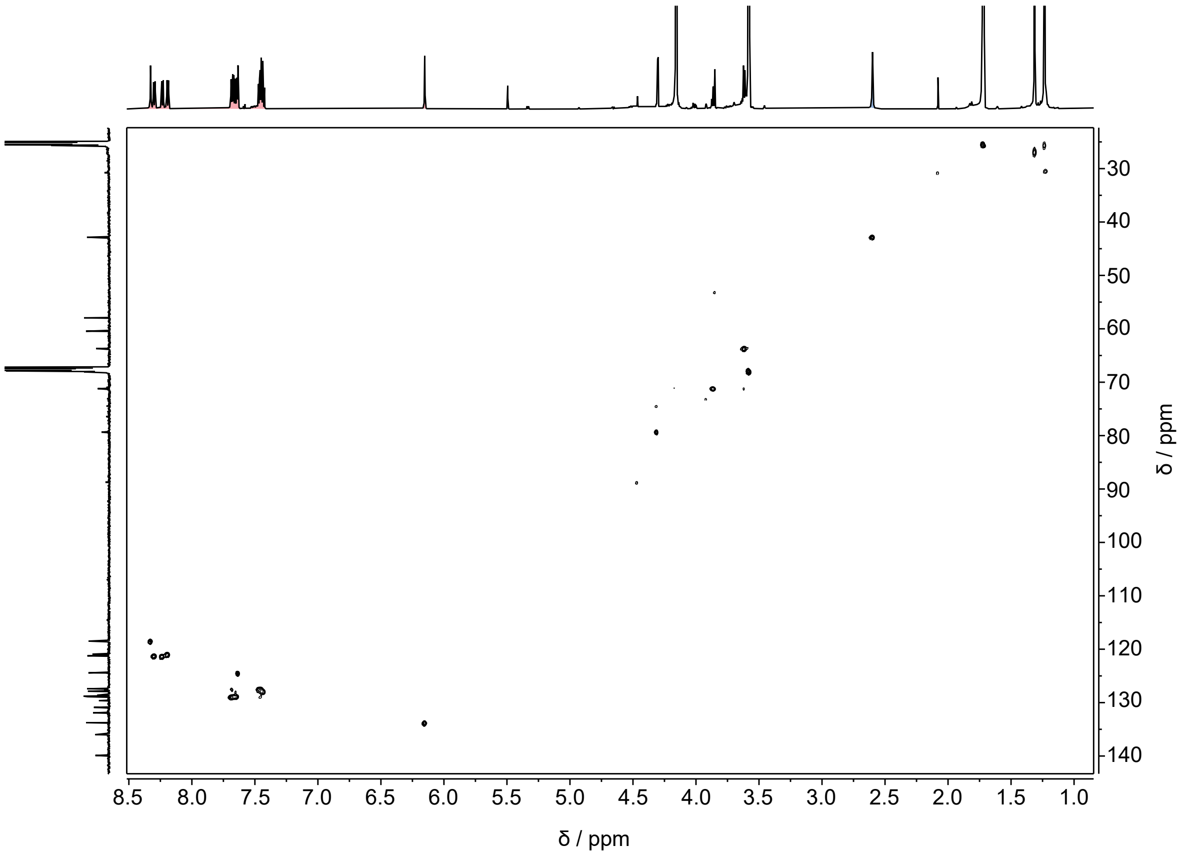

Figure S62: ^{1}H-^{13}C HSQC of per–2-*e*TEMPOH (600 MHz, 298 K, THF-d_8).

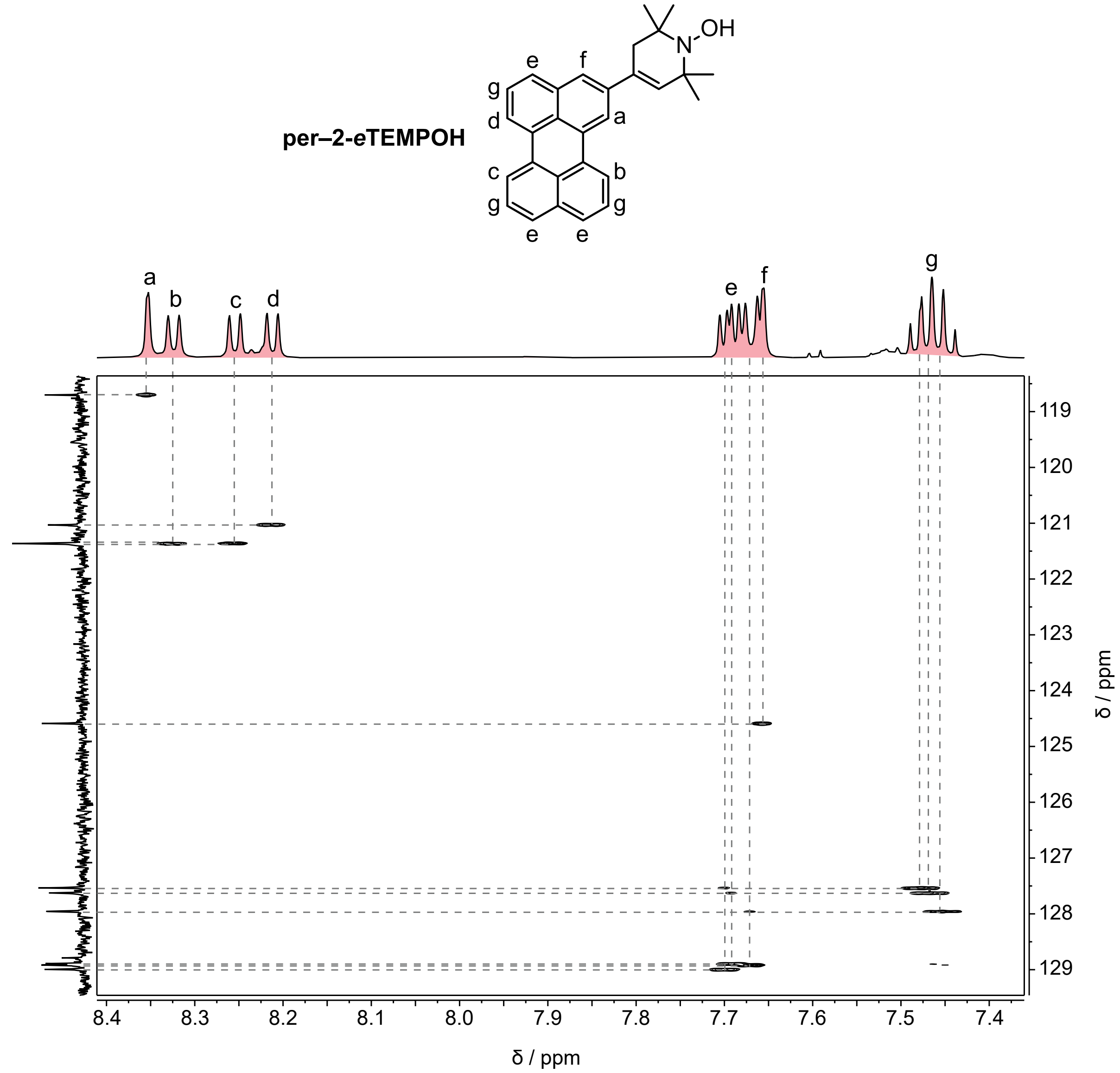

Figure S63: ^{1}H-^{13}C HSQC of per–2-*e*TEMPOH (600 MHz, 298 K, THF-d_8, aromatic region).

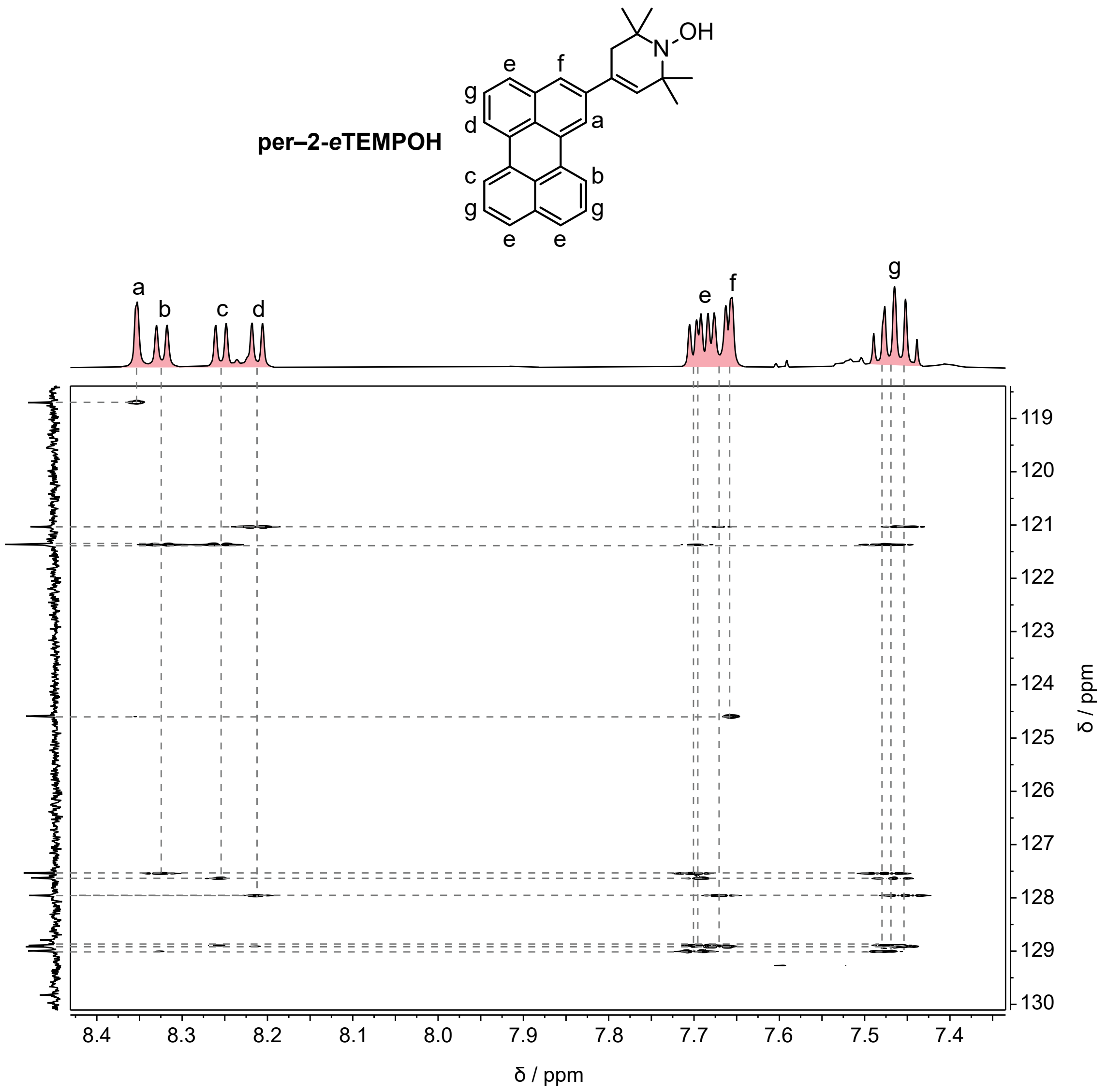

Figure S64: ^{1}H-^{13}C HSQC-TOCSY of per–2-*e*TEMPOH (600 MHz, 298 K, THF-d_8, aromatic region).

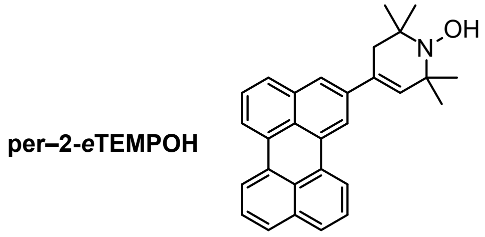

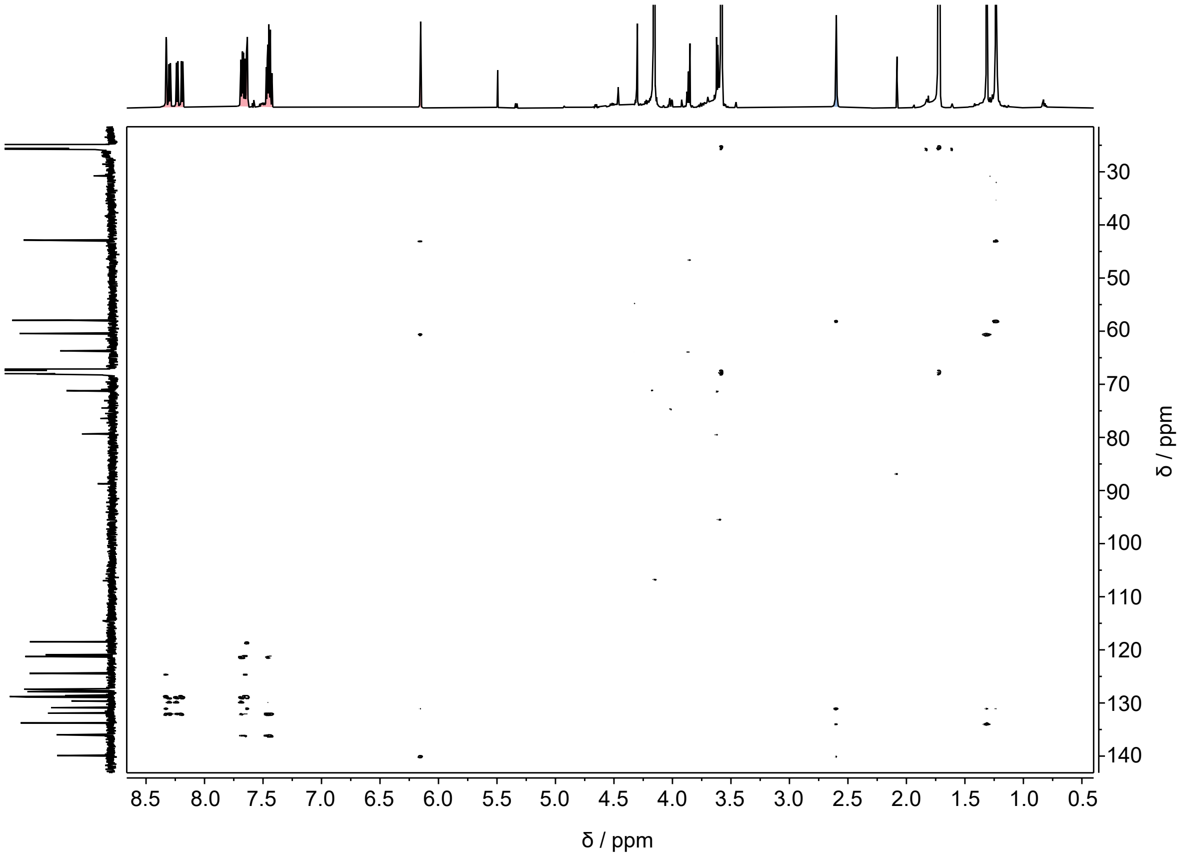

Figure S65: ^{1}H-^{13}C HMBC of per–2-*e*TEMPOH (600 MHz, 298 K, THF-d_8).

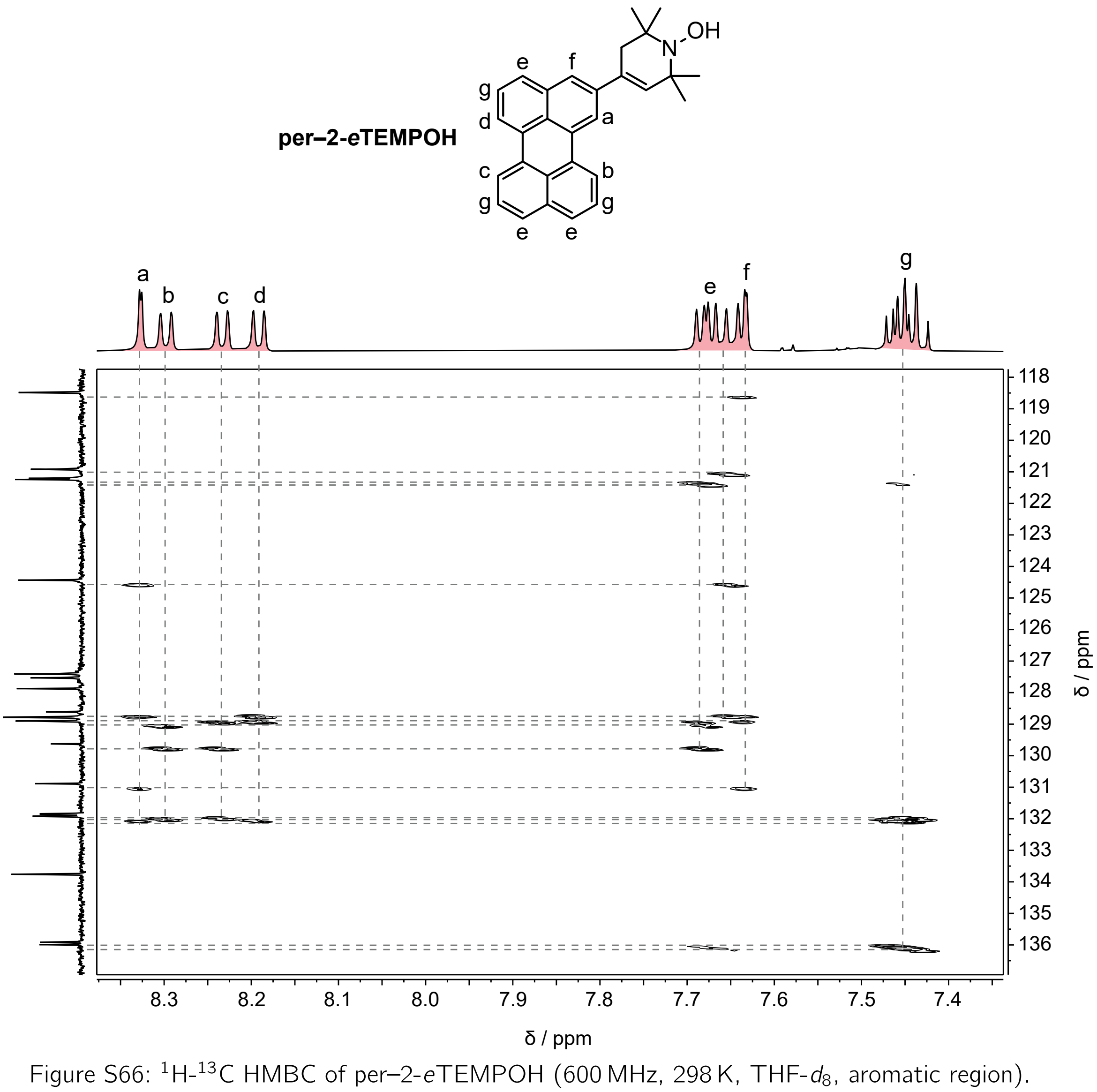

Figure S66: ^{1}H-^{13}C HMBC of per–2-*e*TEMPOH (600 MHz, 298 K, THF-d_8, aromatic region).

6 HRMS data

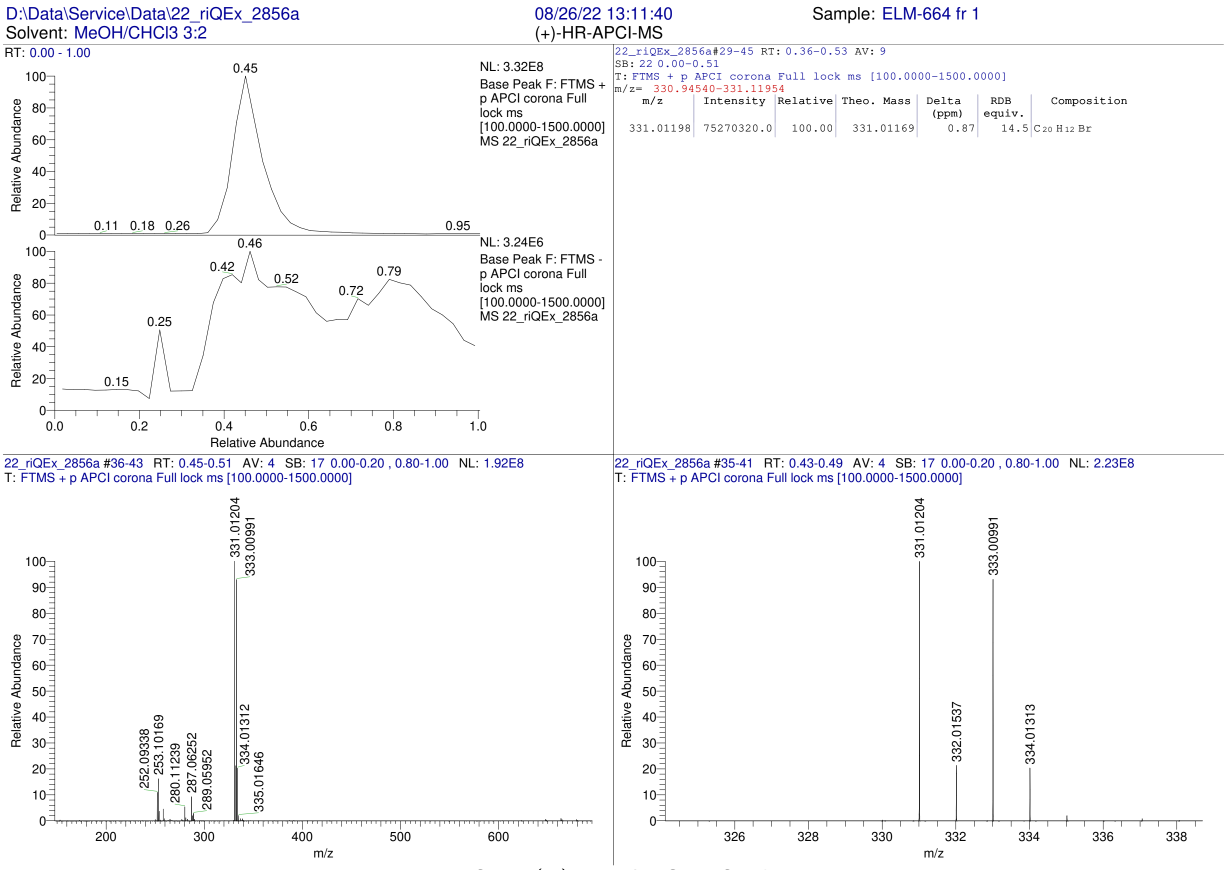

Figure S67: (+)-HR-APCI-MS of 1-Br.

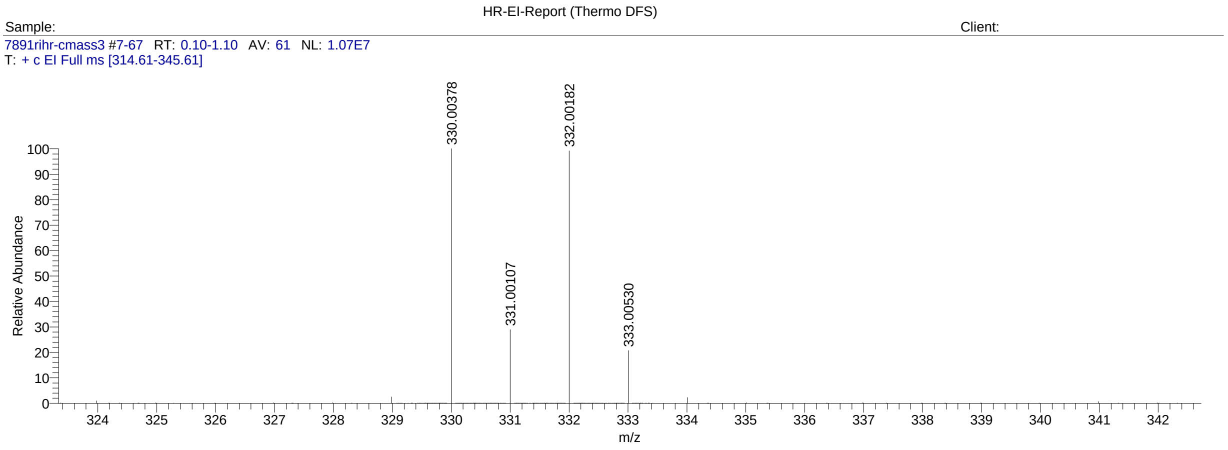

7891rihr-cmass3#7-67 RT: 0.10-1.10 AV: 61
T: + c EI Full ms [314.61-345.61]
m/z= 329.65360-330.32462

m/z	Intensity	Relative	Theo. Mass	Delta (ppm)	Composition
330.00378	10705605.2	100.00	330.00386	-0.26	$C_{20}H_{11}Br$

Figure S68: HR-EI-MS of 2-Br.

D:\Data\Service\Data\23_riQEx_0111 02/06/23 13:59:44 Sample: ELM-719
Client: (+)-HR-ESI-MS Solvent: MeOH/CHCl3 3:2

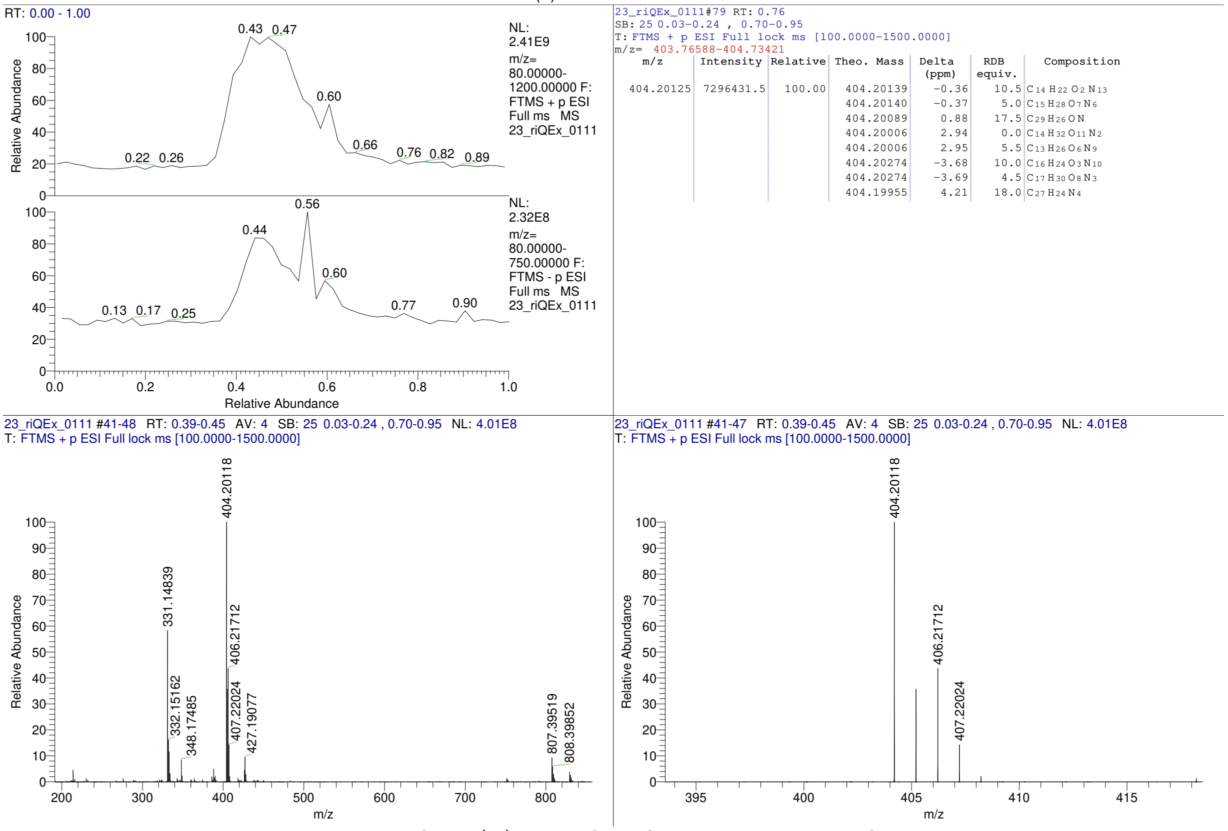

m/z	Intensity	Relative	Theo. Mass	Delta (ppm)	RDB equiv.	Composition
404.20125	7296431.5	100.00	404.20139	-0.36	10.5	$C_{14}H_{22}O_2N_{13}$
			404.20140	-0.37	5.0	$C_{15}H_{28}O_7N_6$
			404.20089	0.88	17.5	$C_{29}H_{26}ON$
			404.20006	2.94	0.0	$C_{14}H_{32}O_{11}N_2$
			404.20006	2.95	5.5	$C_{13}H_{26}O_6N_9$
			404.20274	-3.68	10.0	$C_{16}H_{24}O_3N_{10}$
			404.20274	-3.69	4.5	$C_{17}H_{30}O_8N_3$
			404.19955	4.21	18.0	$C_{27}H_{24}N_4$

Figure S69: (+)-HR-ESI-MS of per–1-*e*TEMPO.

D:\Data\Service\Data\22_riQEx_2842 08/22/22 12:28:42 Sample: ELM-655
Client: (+)-HR-ESI-MS Solvent: MeOH/CHCl3 3:2

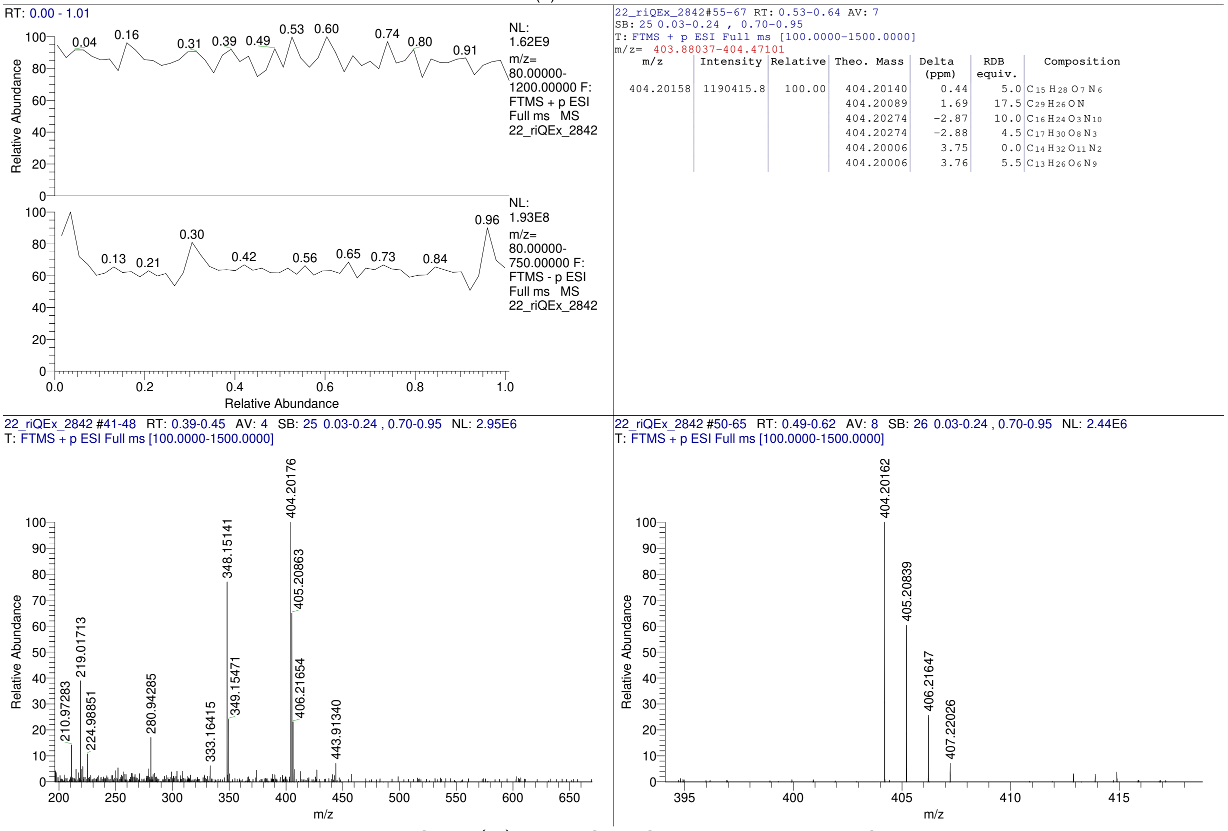

m/z	Intensity	Relative	Theo. Mass	Delta (ppm)	RDB equiv.	Composition
404.20158	1190415.8	100.00	404.20140	0.44	5.0	$C_{15}H_{28}O_7N_6$
			404.20089	1.69	17.5	$C_{29}H_{26}ON$
			404.20274	-2.87	10.0	$C_{16}H_{24}O_3N_{10}$
			404.20274	-2.88	4.5	$C_{17}H_{30}O_8N_3$
			404.20006	3.75	0.0	$C_{14}H_{32}O_{11}N_2$
			404.20006	3.76	5.5	$C_{13}H_{26}O_6N_9$

Figure S70: (+)-HR-ESI-MS of per–2-*e*TEMPO.

HR-ESI Report

Analysis Info

Analysis Name	D:\Data\UZH_Data\Data_2022\Service\Data\22_rihres_0171.d	Acquisition Date	8/11/2022 1:32:30 PM
Method	Service_Syringe_Pump_High_Mass_Range_pos.m	Operator	Demo User
Sample Name	ELM-648	Instrument	timsTOF Pro 1854399.00195
Comment	Solvent: MeCN<br>Client: El Bitar Nehme		

Acquisition Parameter

Source Type	ESI	Ion Polarity	Positive	Set Nebulizer	0.4 Bar
Focus	Not active			Set Dry Heater	200 °C
Scan Begin	50 m/z	Set Capillary	4500 V	Set Dry Gas	3.5 l/min
Scan End	3000 m/z	Set End Plate Offset	-500 V	Set Divert Valve	Source

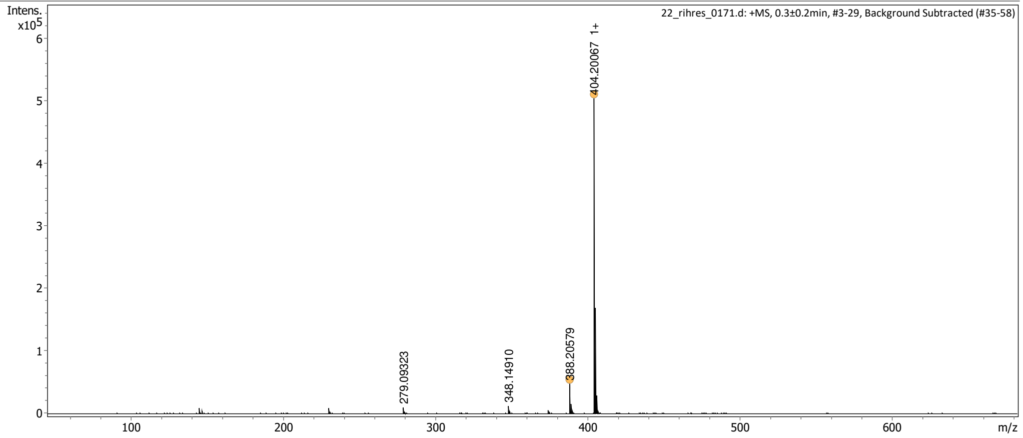

Bruker Compass DataAnalysis 5.3 printed: 8/11/2022 1:46:30 PM by: demo 1 of 2

HR-ESI Report

Meas. m/z	#	Ion Formula	Score	m/z	err [mDa]	err [ppm]	mSigma	rdb	e⁻ Conf
388.20579	1	C29H26N	100.00	388.20598	0.19	0.48	28.6	18.0	even
	2	C14H22N13O	23.20	388.20648	0.69	1.78	69.2	11.0	even
	3	C13H26N9O5	13.86	388.20514	-0.65	-1.67	82.9	6.0	even
	4	H34N7O16	0.17	388.20565	-0.13	-0.35	165.1	-12.0	even
404.20067	1	C29H26NO	100.00	404.20089	0.22	0.55	9.3	18.0	even
	2	C14H22N13O2	10.28	404.20139	0.72	1.79	79.6	11.0	even
	3	C13H26N9O6	6.31	404.20006	-0.61	-1.52	92.4	6.0	even
	4	H34N7O17	0.06	404.20057	-0.10	-0.25	173.7	-12.0	even

Internal calibration

Date:	8/11/2022 1:39:18 PM
Polarity:	Positive
Calibration spectrum:	+MS, 1.3±0.2min, #64-85: Scan
Reference mass list:	ESI: Tuning Mix ES-TOF (ESI)
Calibration mode:	HPC Calibration
Standard deviation:	0.246 ppm

Reference m/z	Resulting m/z	Intensity	Error [ppm]
118.08625	118.08629	72122	0.339
322.04812	322.04809	27213	-0.088
622.02896	622.02906	63782	0.156
922.00980	922.00980	55586	0.002
1221.99064	1221.99062	54921	-0.012
1521.97148	1521.97141	31016	-0.044
1821.95231	1821.95251	13396	0.107
2121.93315	2121.93293	9143	-0.102
2421.91399	2421.91404	4053	0.022
2721.89483	2721.89477	2213	-0.022

Bruker Compass DataAnalysis 5.3 printed: 8/11/2022 1:46:30 PM by: demo 2 of 2

Figure S71: HR-ESI-MS of per–3-*e*TEMPO.

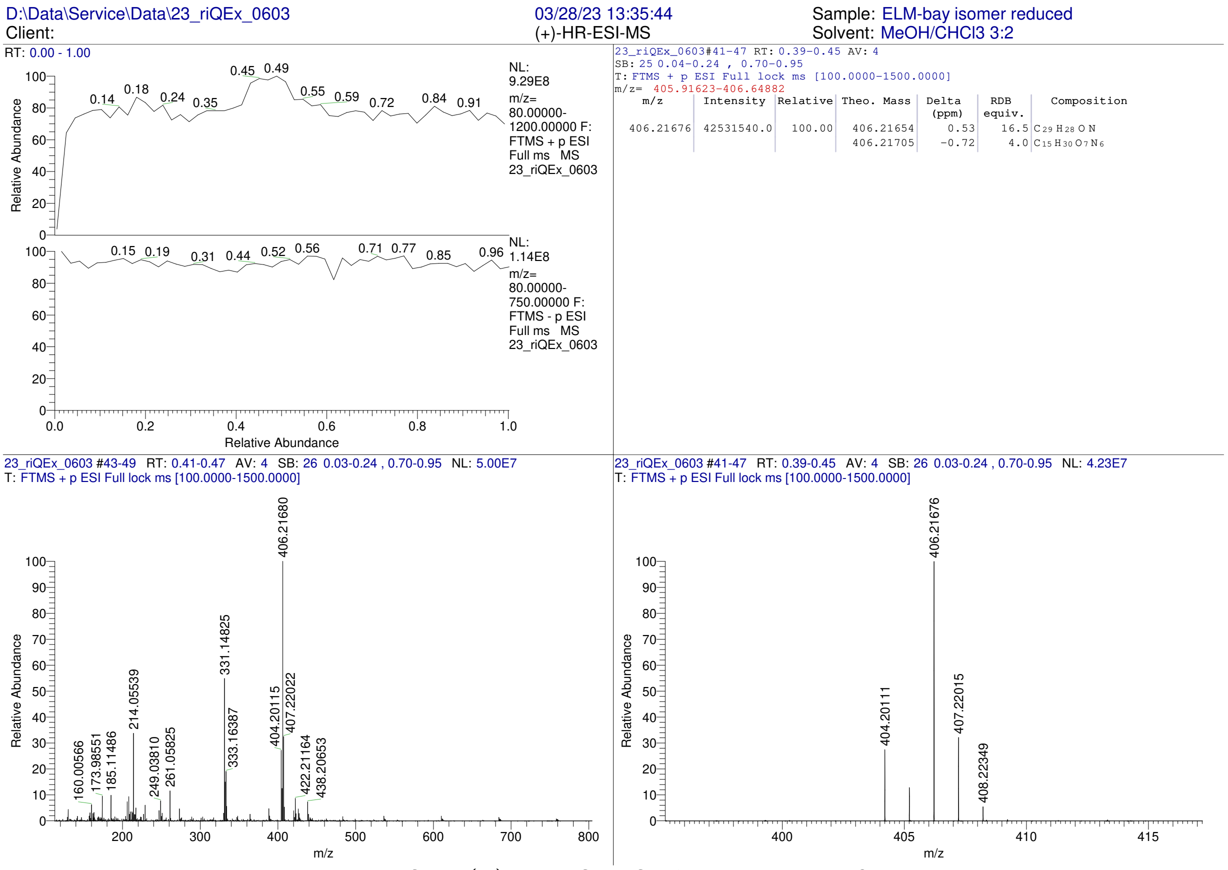

Figure S72: (+)-HR-ESI-MS of per–1-*e*TEMPOH.

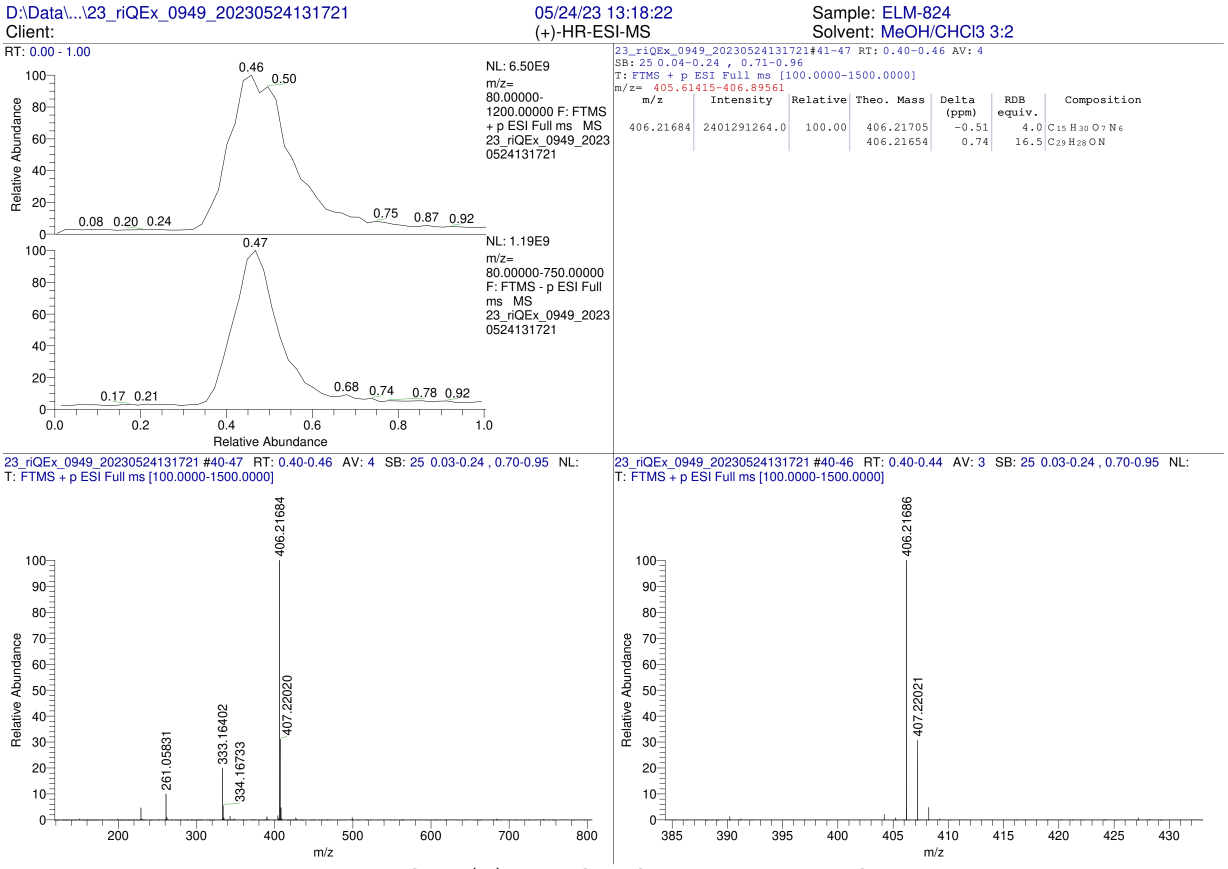

Figure S73: (+)-HR-ESI-MS of per–2-*e*TEMPOH.

References

[1] Carrod, A. J.; Cravcenco, A.; Ye, C.; Börjesson, K. Modulating TTA efficiency through control of high energy triplet states. *J. Mater. Chem. C.* **2022**, *10*, 4923–4928.

[2] Imran, M.; El-Zohry, A. M.; Matt, C.; Taddei, M.; Doria, S.; Bussotti, L.; Foggi, P.; Zhao, J.; Di Donato, M.; Mohammed, O. F., et al. Intersystem crossing via charge recombination in a perylene–naphthalimide compact electron donor/acceptor dyad. *J. Mater. Chem. C.* **2020**, *8*, 8305–8319.

[3] Merz, J.; Dietrich, L.; Nitsch, J.; Krummenacher, I.; Braunschweig, H.; Moos, M.; Mims, D.; Lambert, C.; Marder, T. B. Synthesis, Photophysical and Electronic Properties of Mono-, Di-, and Tri-Amino-Substituted Ortho-Perylenes, and Comparison to the Tetra-Substituted Derivative. *Chem. Eur. J.* **2020**, *26*, 12050–12059.

[4] Hu, M.; Sukhanov, A. A.; Zhang, X.; Elmali, A.; Zhao, J.; Ji, S.; Karatay, A.; Voronkova, V. K. Spiro rhodamine-perylene compact electron donor–acceptor dyads: Conformation restriction, charge separation, and spin–orbit charge transfer intersystem crossing. *J. Phys. Chem. B.* **2021**, *125*, 4187–4203.

[5] Leas, D. A.; Dong, Y.; Vennerstrom, J. L.; Stack, D. E. One-pot, metal-free conversion of anilines to aryl bromides and iodides. *Org. Lett.* **2017**, *19*, 2518–2521.

[6] Henry-Riyad, H.; Tidwell, T. T. Thermolysis of N-tetramethylpiperidinyl triphenylacetate: homolytic fragmentation of a TEMPO ester. *J. Phys. Org. Chem.* **2003**, *16*, 559–563.

[7] Van Geet, A. L. Calibration of methanol nuclear magnetic resonance thermometer at low temperature. *Anal. Chem.* **1970**, *42*, 679–680.

[8] Braun, S.; Kalinowski, H.-O.; Berger, S. *100 and more basic NMR experiments: A practical course*; VCH Weinheim, 1996.

[9] Rickhaus, M.; Jundt, L.; Mayor, M. Determining inversion barriers in atrop-isomers–A tutorial for organic chemists. *Chimia* **2016**, *70*, 192–192.

[10] Berlman, I. B. *Handbook of Fluorescence Spectra of Aromatic Molecules*; Academic Press: New York, 1971.

[11] Strickler, S. J.; Berg, R. A. Relationship between absorption intensity and fluorescence lifetime of molecules. *J. Chem. Phys.* **1962**, *37*, 814–822.

[12] Birks, J. B.; Dyson, D. J.; Flowers, B. H. The relations between the fluorescence and absorption properties of organic molecules. *Proc. R. Soc. Lond. A Math. Phys. Sci.* **1963**, *275*, 135–148.

[13] Lakowicz, J. R. *Principles of fluorescence spectroscopy*; Springer: New York, 2006.

[14] Mayländer, M.; Quintes, T.; Franz, M.; Allonas, X.; Vargas Jentzsch, A.; Richert, S. Distance dependence of enhanced intersystem crossing in BODIPY−nitroxide dyads. *Chem. Sci.* **2023**, *14*, 5361–5368.

[15] Laimgruber, S.; Schachenmayr, H.; Schmidt, B.; Zinth, W.; Gilch, P. A femtosecond stimulated raman spectrograph for the near ultraviolet. *Appl. Phys. B* **2006**, *85*, 557–564.

[16] Laimgruber, S.; Schmierer, T.; Gilch, P.; Kiewisch, K.; Neugebauer, J. The ketene intermediate in the photochemistry of *ortho*-nitrobenzaldehyde. *Phys. Chem. Chem. Phys.* **2008**, *10*, 3872–3882.

[17] Fröbel, S.; Buschhaus, L.; Villnow, T.; Weingart, O.; Gilch, P. The photoformation of a phthalide: a ketene intermediate traced by FSRS. *Phys. Chem. Chem. Phys.* **2015**, *17*, 376–386.

[18] Reiffers, A.; Torres Ziegenbein, C.; Schubert, L.; Diekmann, J.; Thom, K. A.; Kühnemuth, R.; Griesbeck, A.; Weingart, O.; Gilch, P. On the large apparent Stokes shift of phthalimides. *Phys. Chem. Chem. Phys.* **2019**, *21*, 4839–4853.

[19] Lorenc, M.; Ziolek, M.; Naskrecki, R.; Karolczak, J.; Kubicki, J.; Maciejewski, A. Artifacts in femtosecond transient absorption spectroscopy. *Appl. Phys. B* **2002**, *74*, 19–27.

[20] Mayländer, M.; Nolden, O.; Franz, M.; Chen, S.; Bancroft, L.; Qiu, Y.; Wasielewski, M. R.; Gilch, P.; Richert, S. Accessing the triplet state of perylenediimide by radical-enhanced intersystem crossing. *Chem. Sci.* **2022**, *13*, 6732–6743.

[21] van Stokkum, I. H. M.; Larsen, D. S. L.; van Grondelle, R. Global and target analysis of time-resolved spectra. *Biochim. Biophys. Acta Bioenerg.* **2004**, *1657*, 82–104.

[22] Herb, K.; Tschaggelar, R.; Denninger, G.; Jeschke, G. Double resonance calibration of g factor standards: carbon fibers as a high precision standard. *J. Magn. Reson.* **2018**, *289*, 100–106.

[23] Stoll, S.; Schweiger, A. EasySpin, a comprehensive software package for spectral simulation and analysis in EPR. *J. Magn. Reson.* **2006**, *178*, 42–55.

[24] Tait, C. E.; Krzyaniak, M. D.; Stoll, S. Computational tools for the simulation and analysis of spin-polarized EPR spectra. *J. Magn. Reson.* **2023**, *349*, 107410.

[25] Neese, F.; Wennmohs, F.; Becker, U.; Riplinger, C. The ORCA quantum chemistry program package. *J. Chem. Phys.* **2020**, *152*, 224108.

[26] Weigend, F.; Ahlrichs, R. Balanced basis sets of split valence, triple zeta valence and quadruple zeta valence quality for H to Rn: Design and assessment of accuracy. *Phys. Chem. Chem. Phys.* **2005**, *7*, 3297.

[27] Neese, F.; Wennmohs, F.; Hansen, A.; Becker, U. Efficient, approximate and parallel Hartree–Fock and hybrid DFT calculations. A 'chain-of-spheres' algorithm for the Hartree–Fock exchange. *Chem. Phys.* **2009**, *356*, 98–109.

[28] Frisch, M. J. et al. Gaussian 16 Revision C.01. 2016; Gaussian Inc. Wallingford CT.

[29] Chai, J. D.; Head-Gordon, M. Long-range corrected hybrid density functionals with damped atom-atom dispersion corrections. *Physical Chemistry Chemical Physics* **2008**, *10*, 6615–6620.

[30] McLean, A. D.; Chandler, G. S. Contracted Gaussian basis sets for molecular calculations. I. Second row atoms, Z =11–18. *The Journal of Chemical Physics* **1980**, *72*, 5639–5648.

[31] Krishnan, R.; Binkley, J. S.; Seeger, R.; Pople, J. A. Self-consistent molecular orbital methods. XX. A basis set for correlated wave functions. *The Journal of Chemical Physics* **1980**, *72*, 650–654.

[32] Tomasi, J.; Mennucci, B.; Cammi, R. Quantum mechanical continuum solvation models. *Chemical Reviews* **2005**, *105*, 2999–3094.
